# Toward a standardized framework for pangenome graph evaluation: assessing crop plant pangenome variation graph construction from multiple assemblies

**DOI:** 10.1093/gigascience/giaf121

**Published:** 2025-12-04

**Authors:** Venkataramana Kopalli, Kübra Arslan, Noemia Morales-Díaz, Silvia F Zanini, Agnieszka A Golicz

**Affiliations:** Department of Agrobioinformatics, IFZ Research Centre for Biosystems, Land Use and Nutrition, Justus Liebig University Gießen, 35392 Gießen, Germany; Department of Agrobioinformatics, IFZ Research Centre for Biosystems, Land Use and Nutrition, Justus Liebig University Gießen, 35392 Gießen, Germany; Centre for Research in Agricultural Genomics, CRAG (CSIC-IRTA-UAB-UB) Campus UAB, Cerdanyola del Vallès, 08193 Barcelona, Spain; Department of Agrobioinformatics, IFZ Research Centre for Biosystems, Land Use and Nutrition, Justus Liebig University Gießen, 35392 Gießen, Germany; Department of Agrobioinformatics, IFZ Research Centre for Biosystems, Land Use and Nutrition, Justus Liebig University Gießen, 35392 Gießen, Germany

**Keywords:** pangenome graph, structural variation, benchmarking, crop genomics

## Abstract

**Background:**

Pangenomes are crucial for understanding species-wide genetic diversity, delineating core and variable genes. This study compares 3 key pangenome graph assembly pipelines: Minigraph, PGGB, and Minigraph-Cactus, using publicly available Sorghum data. We introduce tailored metrics for comprehensive pangenome graph evaluation, including completeness, duplication levels, and fidelity of structural variants.

**Results:**

By assessing the tools on Sorghum datasets, we gauge their efficacy in handling diverse genomic features. The analysis provides detailed insights into the strengths and limitations of Minigraph, PGGB, and Minigraph-Cactus, aiding researchers in informed tool selection. The metrics developed contribute to standardizing pangenome graph assessments, enabling robust and objective tool comparisons. We further demonstrate the utility of the metrics by applying them to pangenome graphs of 3 crops: soybean, barley, and oilseed rape.

**Conclusions:**

This benchmarking study advances our understanding of pangenome assembly tools and establishes a foundation for standardized evaluation metrics. We plan to further use these insights to optimize tool selection for specific applications, such as genome-wide association studies, improving the accuracy of downstream analyses.

## Introduction

Pangenomics, a field focused on capturing the complete genetic diversity within a species, has gained significant attention in genomics, agriculture, and microbial studies [1]. Pangenomes offer a more comprehensive view of genetic variation within populations, species, or genera by moving beyond the limitations of a single reference genome, such as its failure to represent the full genetic diversity, introduce reference bias, and overlook structural variants [2]. Pangenomes are structured collections of genomic data that enable the study of genetic variant relationships while preserving the continuity of sequences and structural variations across individuals, allowing for the identification of complex DNA polymorphisms like structural variations (SVs), copy number variations (CNVs), and presence/absence variants (PAVs) [3, 4].

Pangenomes have been applied in plant research to understand genetic mechanisms underlying trait variation, accelerate breeding processes, and improve crop performance [5]. Pangenomes allow for the capture of genetic diversity essential for enhancing agronomical traits by incorporating wild species and multiple varieties and have been instrumental in studying transposable elements and accessory genomes, shedding light on their impact on genetic variation [6]. Pangenome analysis has been particularly valuable in crops like rice (*Oryza sativa*), maize (*Zea mays*), and rapeseed (*Brassica napus*), where it has revealed previously unknown genetic variations, aiding in accelerated genetic improvement [7–9].

Sorghum’s significance for pangenome research lies in its genomic characteristics in addition to its applications in food, feed, or biofuel. Sorghum has a relatively small (~730 Mb) diploid genome (2n = 20), which simplifies genome assembly. Repeat content constitutes approximately 61% of its genome, significantly influencing its SV [10]. Studies have identified extensive single-nucleotide polymorphisms (SNPs), insertions/deletions (indels), and large-size PAVs that contribute to sorghum’s genetic diversity, an important factor for breeding [11]. Additionally, the pangenome approach in sorghum has revealed crucial structural variations, especially in genes related to stress responses, resistance to diseases, and adaptation to various environments. Known structural variants include numerous large-size PAVs and mobile elements, which further diversify sorghum’s genome. This genetic variability is valuable for understanding the evolutionary dynamics and guiding crop improvement strategies [12, 13].

Several tools and pipelines enable the creation of pangenome variation graphs, which are a graph-based representation of multiple genomes. These graphs model genetic diversity by encoding genomic sequences as nodes and their relationships, such as continuity or variation (e.g., SNPs, insertions, deletions, and structural variations), as edges. This allows for the efficient comparison, alignment, and analysis of genetic variation within a population. Minigraph, PanGenome Graph Builder (PGGB), and Minigraph-Cactus are among these tools and pipelines used for these purposes. Minigraph, PGGB, and Minigraph-Cactus were selected as they are among the most widely used and well-regarded tools for constructing pangenome graphs, offering robust and complementary capabilities. Minigraph is a versatile tool designed to construct and manipulate sequence graphs, is particularly effective in handling large SVs, and is optimized for speed and scalability. It constructs pangenomes by aligning multiple genome assemblies against a reference, facilitating the detection of large insertions, deletions, and complex rearrangements [14], and was used to build the pangenome graph database representing presence/absence variation across 16 bread wheat genomes [15]. PGGB is an innovative, reference-free pipeline designed to construct unbiased pangenome graphs. By utilizing all-versus-all whole-genome alignments and advanced graph embeddings, PGGB builds and continuously refines a model that allows for the identification of genetic variation, assessment of conservation, detection of recombination events, and inference of phylogenetic relationships [16]. It was utilized to build a pangenome graph for a comprehensive genetic variation analysis of *Neisseria meningitidis* [17]. Minigraph-Cactus pipeline combines 2 methodologies: Minigraph and Cactus. Minigraph excels in building compact and accurate graphs from multiple genomes by aligning reads to a reference graph, while Cactus enhances this by integrating these graphs into a comprehensive pangenome structure. This combination facilitates both visualization and exploration of genetic variation, structural differences, and evolutionary relationships across diverse genomes, offering valuable insights into genomic diversity and complexity [18]. This pipeline was successfully utilized to build a pangenome graph and reveal extensive effector copy-number variation in spinach downy mildew [19].

Unlike linear pangenomes, which aim to encode all variation in a linear data structure built around a reference genome [1, 20], pangenome variation graphs encode multiple haplotypes, alleles, and structural variations directly into the graph topology [15, 21, 22]. As a result, these graph-based models preserve complex variant relationships and allow for improved alignment and variant discovery across divergent genotypes. This is particularly advantageous for crops like sorghum, where large structural variations, presence/absence variants, and repetitive content are prevalent. Graph-based approaches can reduce false negatives in SV detection, improve read mapping accuracy in structurally complex regions, and enable downstream analyses such as graph-based genotyping and structural variant and haplotype-aware association studies [23–25] As such, pangenome graphs offer a powerful alternative to linear [22] approaches for capturing true biological diversity in plant genomes [26, 27].

SV detection and comparative genomics have traditionally relied on linear reference-based approaches to identify genomic differences between individual assemblies. To fully understand the benefits of a pangenome-based approach, we compared Minigraph, PGGB, and Minigraph-Cactus with 2 linear SV callers, SVIM-asm and SyRI. SVIM-asm is a linear variant caller designed specifically for long-read assemblies, enabling the accurate detection of complex structural variants, such as insertions, deletions, duplications, and translocations, by leveraging the detailed structural information encoded in such assemblies [28]. SyRI is a comprehensive tool for predicting genomic differences between related genomes using whole-genome assemblies (WGAs). The assemblies are aligned using whole-genome alignment tools, and these alignments are then used as input to SyRI. SyRI identifies the syntenic path (longest set of colinear regions), structural rearrangements (inversions, translocations, and duplications), local variations (SNPs, indels, CNVs, etc.) within syntenic and structural rearrangements, and unaligned regions [29]. In this article, we report extensive comparisons of the 3 leading pangenome variation graph construction pipelines using simulated and real-world data representing a sorghum diversity set. We assess the properties of the resulting pangenome graphs in terms of their completeness and encoded variation, but also suitability for downstream applications, including read mapping. Our work expands the knowledge base and toolbox necessary to build high-quality pangenome graphs in plants and especially crops, which often boast genomes affected by high levels of duplication, sequence diversity, and transposable element activity [30, 31].

## Methods and Tools

**Figure 1:**
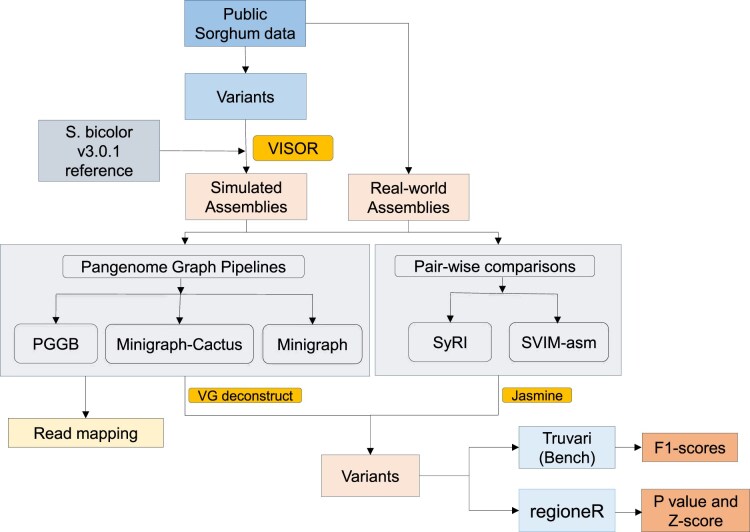
This flowchart illustrates the workflow used in our study to evaluate pangenome graph-building pipelines. Public sorghum data were used to generate simulated assemblies, which, along with real-world assemblies, were analyzed using pangenome graph-building pipelines and pairwise comparison tools. The resulting outputs were then assessed to evaluate their performance and accuracy.

This study employed a comprehensive workflow encompassing genome assembly, chromosome splitting, simulated genome generation, pangenome construction, variant calling, and downstream analyses. The workflow, as shown in Fig. 1, provides an overview of the key steps, tools, and methods used across the study, linking each stage of the analysis pipeline.

### Genome assembly and chromosome splitting

Six assemblies were chosen based on the phylogenetic relationships published in a study by Tao et al. [32]. We chose the accessions to be as diverse as possible to capture extensive variation. All 6 sorghum assemblies used were generated using a hybrid sequencing approach and exhibit high contiguity (contig N50 up to 3.5 Mb) and completeness (>95% BUSCO). We included 2 *Sorghum bicolor* (IS8525 and Rio), 1 *Sorghum bicolor verticilliforum* (AusTRCF317961), 1 *Sorghum bicolor drummondii* (PI532566), 1 *Sorghum bicolor margaritiferum* (IS19953), and an outlier *Sorghum propinquum* (S369-1). Publicly available sorghum genome assemblies were downloaded. [32]. Similarly, publicly available assemblies (Supplementary Table S7) for rapeseed [9], barley [33], and soybean [24] included in this study were obtained. The reference genomes used were Darmor v10 [34] for rapeseed, MorexV3 [35] for barley, and *Glycine max* v4.0 [36] for soybean. The assemblies were then split into individual chromosome files using a shell script.

### Simulated genome assemblies

Simulated genomes for all 6 accessions were generated using VISOR (version 1.1.2.1) [37], with *S. bicolor* v3.0.1 as the reference backbone, incorporating real-world SVs identified in the 6 accessions. A filtering step was applied to retain only those SVs present in at least 2 of the 6 assemblies due to a large number of variants unique to 1 genotype prior to genome simulation. After filtering, 36,358 SVs remained, and all those exceeding 100 bp in size were used to simulate assemblies.

The headers in the FASTA files of the genome assemblies were modified to include the genome name as a suffix to the chromosome name. This adjustment simplified tracking individual assemblies and ensured efficient downstream analysis.

### Pangenome construction

Pangenomes were constructed per chromosome for the 10 chromosomes of *S. bicolor* using PGGB, Minigraph-Cactus, and Minigraph for real-world and simulated datasets separately. The methods of construction were the same for both datasets.

#### PGGB (version 0.5.4)

For each of the chromosomes, we created a FASTA file containing the corresponding 7 sequences, 6 from the selected accessions and 1 from the reference. We compressed the resulting FASTA and created an index (.fai) file using samtools (version 1.18) [38] faidx. FASTA and index files were used as input for graph construction, running PGGB with default parameters (*k* = 19, *p* = 90, *s* = 5,000). The order of the input samples was deemed insignificant as PGGB performs an all-versus-all alignment.

#### Minigraph-cactus (version 2.7.2)

The Minigraph-Cactus tool was used with default parameters, using the *S. bicolor* v3.0.1 as the reference along with the 6 genome assemblies. The command was run with –gfa, –viz, –draw, –og, and –gbz parameters. Minigraph-Cactus calculates the mash distance, which is a metric used to estimate the genetic distance or similarity between 2 genome assemblies, and reorders the input assemblies by placing the reference assembly first, followed by the remaining assemblies in decreasing order of mash distance to the reference.

#### Minigraph (version 0.21)

The Minigraph pangenome was constructed for each chromosome of the assemblies with the *S. bicolor* v3.0.1 reference as a backbone. Default parameters were used. Since Minigraph does not provide instructions for determining the order of input samples, a random order was used, assuming that the order is insignificant.

#### BUSCO (version 6.0.0)

We assessed the completeness of the newly generated graphs with BUSCO, a bioinformatics tool that scores genome assemblies, gene sets, and transcriptomes by comparing them against a set of highly conserved orthologs. BUSCO evaluates the presence and quality of expected single-copy orthologs in the given dataset, providing insights into the completeness and potential gaps in the assembly [39].

We used the odgi flatten command from odgi tools (version 0.8.6–0-ge647844f) [40] to convert the OG files of pangenomes of each chromosome into FASTA format and merged all the resulting FASTA files to reconstitute full assemblies. We ran BUSCO with the poales_odb10 lineage dataset and genome mode to assess the completeness of the pangenome.

#### Panacus (version 0.2.3)

Panacus is a tool designed for quantifying the core size and estimating growth curves in pangenome graphs. It calculated several key statistics, including pangenome growth and core curves, by counting nodes, edges, and base pairs, as well as tracking how they were covered across paths. This tool enabled the grouping of paths, focusing on specific regions, and generated clear, interactive reports [41].

To analyze pangenome graphs, we first selected the paths corresponding to haplotypes by filtering and excluding specific references. We then used Panacus to calculate the coverage and pangenome growth for nodes with varying coverage and quorum thresholds (1/0, 2/0, 1/1, 1/0.5, and 1/0.1), using up to 4 threads. The resulting data were output as a TSV file. Finally, coverage histograms and pangenome growth curves were visualized, including estimated growth parameters (Supplementary Fig. S2).

### Variant calling and filtering

#### PGGB and Minigraph-Cactus

PGGB and Minigraph-Cactus variants were called with VG deconstruct (version 1.54.0) [42]. Variants were called with respect to the path of the reference genome, along with parameters to process all snarls (-a) and to only consider traversals that corresponded to paths in the graph (-e). The input file was the GFA file output of PGGB, and this process was repeated for each chromosome-graph. The variants were filtered to include only variants with alternative alleles, and the multiallelic variants were split using bcftools norm (version 1.16–23) [38] to facilitate downstream analysis.

#### Minigraph

We added path information (P-lines) to Minigraph’s GFA by manually curating the output of minigraph -call, which retraced the assembly’s path. We then called the variants with VG deconstruct.

#### SVIM-asm (version 1.0.3)

SVIM-asm is a structural variant caller for haploid or diploid genome–genome alignments. We ran SVIM-asm separately for each assembly against the *S. bicolor* v3.0.1 reference as it calls SVs from pairwise assemblies aligned with Minimap2 (version 2.26-r1175) [43]. SAM outputs were converted to BAM using samtools, and then BAM files were sorted and indexed to be used as inputs to SVIM-asm in haploid mode to call the variants.

#### SyRI (version 1.6.3)

We used SyRI to compare chromosome-level assemblies and identify structural rearrangements and synteny. The same pairwise alignments generated with Minimap2 for SVIM-asm were used as input for SyRI variant calling. The initial VCF file produced by SyRI was missing genotype information and flagged “N”s as highly diverged regions (HDRs). We resolved this by filtering out the “N”s, manually adding FORMAT and SAMPLE columns, and updating the VCF header for compatibility with bcftools. After merging the individual VCF files for each method with Jasmine (v1.1.5) [44], we refined the results by sorting, using bcftools sort and splitting multiallelic variants using bcftools norm.

#### Truvari Bench (version 4.1.0)

To assess the accuracy of both variant callers, we calculated and compared the precision, recall, and F1 scores of called variants from the pangenomes against the true set of variants from Tao et al. [32], which was used to simulate the genome assemblies. Truvari bench was run with default parameters [32, 45].

#### regioneR (version 1.36.0)

We used permutation testing with the regioneR package to evaluate the performance of PGGB and Minigraph-Cactus variant calling with real-world assemblies [46]. It enabled the assessment of overlaps between genomic regions by comparing observed data against a distribution generated from randomized genomic regions to determine statistical significance.

We filtered out pangenome variants longer than the longest variant in the true set, as their inclusion disproportionately increased the expected overlap during the regioneR permutation analysis. Longer variants had a higher likelihood of overlapping by chance due to their size, which affected the randomization process and inflated the baseline expected overlap. The permutation test was conducted using the permTest function, with 100 permutations of genomic regions. The randomizeRegions function was employed to generate these randomized regions, and the numOverlaps function was used to evaluate the overlap between the variant sets and the true variants. We set count.once=TRUE to ensure that each true-set variant was counted only once, avoiding overestimation from multiple overlaps with the same pangenome variant.

#### Surpyvor (version 0.5)

Surpyvor is a Python package designed for analyzing SVs, enabling the visualization and quantification of SV relationships within genomic data [47]. We utilized the venn function for creating Venn diagrams and the upset function for generating UpSet plots. For large variant comparisons, we used the default parameters; however, for small variant comparisons, we used the -snv option to override the default SURVIVOR method and opted for bcftools instead, as the SURVIVOR method was primarily tailored for large SV analyses.

### Repeat content analysis

To assess repeat content in the pangenome variants, we first generated a transposable element (TE) library using the EDTA (Extensive de novo TE Annotator) pipeline (version 2.1.1) [48]. EDTA was applied to generate a nonredundant high-quality TE library. This library was then formatted as a BLAST database with makeblastdb to allow for efficient sequence comparison. Variant sequences from the VCF file were extracted and formatted in FASTA, with each sequence labeled by its genomic coordinates for unique identification. Sequence headers were standardized to remove any noncompatible characters to facilitate downstream analysis. We then used BLASTN (version 2.12.0) [49] to align these variant sequences against the TE database, retrieving only the top hit for each variant. The results were sorted by query coverage, and only the best alignment for each variant was retained. We further filtered the results to include only those matches with a query coverage of 80% or higher to ensure high confidence in the repeat classification. This approach enabled us to reliably identify and characterize repeat-associated variants within the pangenome.

### Missing genes and assembly coverage statistics

To detect missing genes across assemblies in the pangenome graph, we first generated FASTA sequences for each assembly’s path in the graph using odgi paths. Gene annotations from the reference genome were mapped to each pangenome assembly FASTA with Liftoff (version 1.6.3) [50], identifying unmapped genes as potentially missing. Each assembly was also aligned to its path in the pangenome graph using Minimap2. The alignments were processed with samtools to generate coverage statistics.

### Read simulation and mapping

We evaluated the performance of linear and graph mapping by using simulated reads, which were generated using the code developed by Rice et al. [51]. This process involved 3 main steps: first, simulating reads from the graph; second, aligning those reads to both the graph using VG Giraffe (version 1.58.0) [52] and to the *S. bicolor* v3.0.1 linear reference using Minimap2; and, finally, comparing the results from both mapping methods based on the alignment quality and correctness rates.

Individual OG files generated for each chromosome were merged with odgi squeeze. The merged OG file was converted back to GFA format with odgi view.

Read simulation: We simulated 1 million reads from the corresponding pangenome graphs (which were then used as reference for read mapping) using VG sim [53]. The length of each simulated read was set to 150 bps, the error rate was set at 0.24%, the indel rate was set to 0.029%, the mean insert size was set to 570 bps, and the standard deviation of the insert size was set to 165. The resultant GAM file was converted to FASTQ format with VG view.

Mapping to VG Giraffe: The simulated reads were mapped back to the graph using VG Giraffe. To enable VG Giraffe to map reads to the graph, indexes were generated using VG autoindex with the workflow parameter set to Giraffe, which produced the necessary files for mapping (.gbz, .min, and .dist). We annotated the GAM file and compared it to the simulated reads using VG gamcompare, generating cumulative alignment and correctness rates.

Mapping to linear reference: We extracted reference paths from the graph using VG paths, mapped the simulated reads with Minimap2, and injected the alignment back into the graph. We annotated the GAM file and compared it to the simulated reads using VG gamcompare, generating cumulative alignment and correctness rates.

Real-world reads: We downloaded paired-end reads datasets of sorghum [32] to test mapping efficiency in a real-world scenario. Reads were mapped to the graph using VG Giraffe and to the linear reference using Minimap2, with the same parameters as the simulated reads.

### Additional species benchmarking

To evaluate the generalizability of our benchmarking results and minimize species-specific bias, we extended our pangenome construction and evaluation pipeline to 3 additional plant species: *Glycine max* (soybean), *Brassica napus* (rapeseed), and *Hordeum vulgare* (barley). These species were selected to represent a diversity of genome sizes, ploidy levels, and assembly qualities. For each species, we applied the same pangenome construction methods (PGGB, Minigraph-Cactus, and Minigraph) using publicly available real-world genome assemblies. All steps, including chromosome-wise graph construction and downstream analysis, were performed as described for *S. bicolor*, but using only real-world assemblies.

### *K*-mer–based analysis of sequence duplication in graphs

Along with evaluating BUSCO scores for these crops, we also implemented an additional method to estimate duplication levels in the pangenomes, which was designed to overcome the limitation of fragmentation of BUSCO genes by the graph structure. For each crop, we first performed BUSCO analysis on the reference genomes (Supplementary Table S3) and extracted the coding sequences (CDSs) corresponding to “Complete” BUSCOs. We then generated 21-mers from these CDS regions and aligned them using bwa aln [54] to both the input assemblies used for pangenome construction. We retained only those *k*-mers that uniquely mapped to all input genomes by filtering the alignments for the “X0:i:1” tag in the SAM files. These *k*-mers were then mapped to the reference genome and the corresponding flattened graph FASTA. By analyzing the proportion of uniquely (X0:i:1) and multiply mapped *k*-mers in these alignments, we assessed the extent of duplication across the pangenome graphs.

## Results

### Simulated assemblies

#### Pangenome size and content

**Figure 2:**
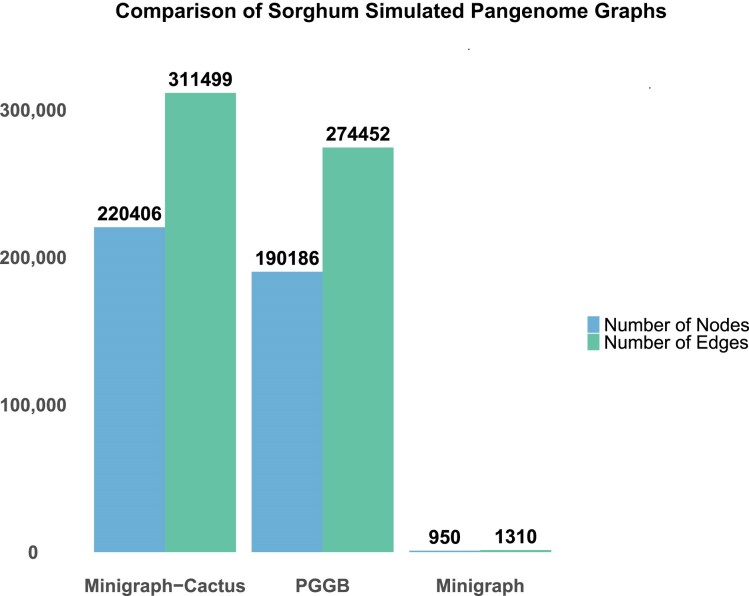
Comparison of the number of nodes and edges for Minigraph-Cactus, PGGB, and Minigraph pangenome graphs constructed from simulated assemblies.

The size of the pangenomes was similar between pipelines, while the number of nodes greatly varied across methodologies. The PGGB pangenome consisted of 851 Mb with ~190,000 nodes, the Minigraph-Cactus pangenome was 795 Mb with ~220,000 nodes, and the Minigraph pangenome was 878 Mb with only 950 nodes (Fig. 2, Supplementary Table S5b). The linear reference genome had a size of 688 Mb, demonstrating that all pangenomes exhibited an increase in total genomic content compared to the linear reference (Supplementary Table S5b). This highlighted the additional complexity and variation encompassed by the pangenome graph. Minigraph stood out in our simulation, as it failed to detect variants, which resulted in a smaller number of very long nodes. The average node length for Minigraph was 968,806 bp, which was very high compared to PGGB’s 4,517 bp and Minigraph-Cactus’s 3,714 bp. The mean BUSCO completeness for the 6 input simulated assemblies was 98.4%, with 1.6% duplication, comparable to BUSCO completeness of 98.4%, with 1.7% duplication seen in the 3 pangenomes (Supplementary Table S3b).

#### Variant calling

**Figure 3:**
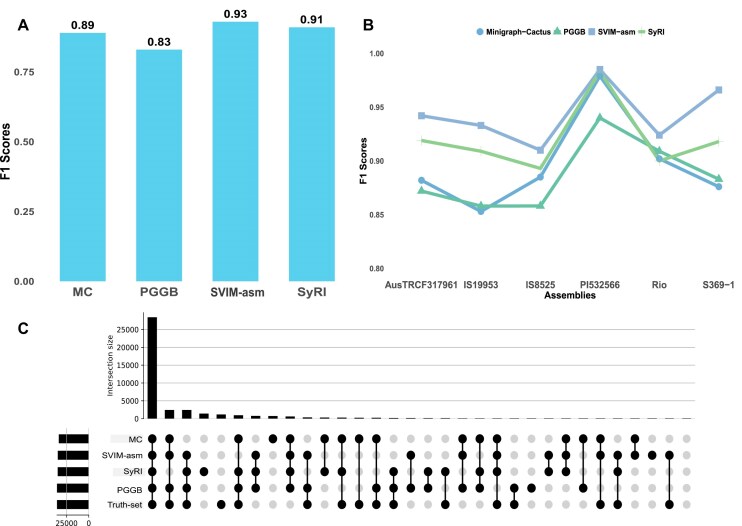
Benchmarking structural variant detection across tools using simulated data. (A) F1 score comparison for the nonredundant merged variant set across all assemblies shows SVIM-asm achieving the best performance (0.93), followed closely by SyRI (0.91) and Minigraph-Cactus (0.89), while PGGB has the lowest score (0.83). (B) Per-sample F1 score distribution reveals consistent performance trends across samples for all methods, with SVIM-asm and SyRI generally outperforming graph-based approaches. (C) UpSet plot showing overlap in SV calls among SyRI, SVIM-asm, PGGB, and Minigraph-Cactus with the ground truth. The largest shared intersection is among all 4 tools.

To assess the accuracy of variant calling, we calculated F1 scores for variants identified from pangenomes constructed with simulated assemblies of Minigraph, PGGB, and Minigraph-Cactus. This comparison was made against a set of true variants from a published dataset using the Truvari benchmarking tool. The average F1 score for PGGB was 0.83, while Minigraph-Cactus achieved a higher average F1 score of 0.89 (Fig. 3A). Minigraph detected a considerably low number of variants in a simulated scenario, an unexpected behavior also reported by other users [55, 56]. The results were consistent when cross-checking the Minigraph output with the Minigraph output from the Minigraph-Cactus pipeline. The limited number of variants detected by Minigraph prevented the calculation of a meaningful F1 score, and the output did not provide sufficient data for reliable evaluation.

The variants were also called using linear-based SVIM-asm and Syri (Fig. 3A) to compare the variant calling efficiency of graph-based PGGB, Minigraph-Cactus, and Minigraph. The F1 score comparisons across all assemblies suggested that pairwise comparison methods outperformed pangenome graph pipelines in the SV calling task. SVIM-asm achieved the highest overall F1 score of 0.93, followed by SyRI at 0.91, both outperforming Minigraph-Cactus (0.89) and PGGB (0.83) (Fig. 3A). SVIM-asm and Syri also exhibited higher consistency and F1 scores across multiple genome assemblies (Fig. 3B).

Pairwise variant callers offer slightly better variant detection accuracy and robustness across different genome assemblies compared to pangenome construction pipelines, as shown in Fig. 3B in a simulated scenario. Nevertheless, most SVs were detected across all methodologies (Fig. 3C), demonstrating the suitability of graph-based approaches while revealing differences in sensitivity and specificity across tools.

### Real-world assemblies

#### Pangenome size and content

**Figure 4:**
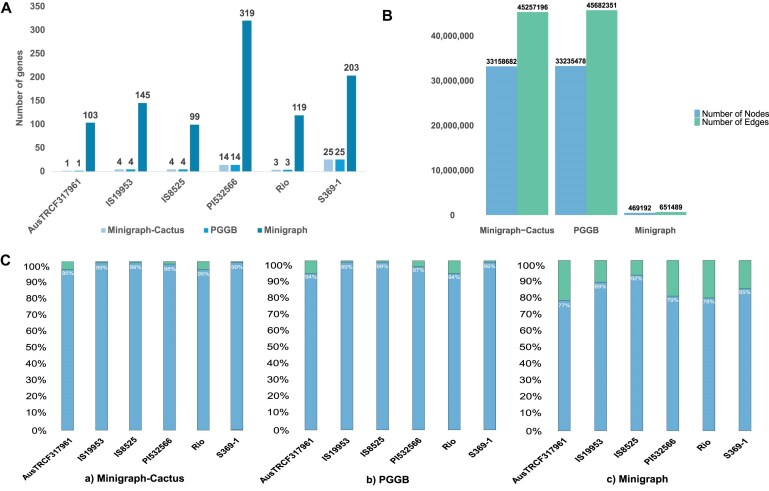
Comparison of key graph characteristics and gene representation across pangenome graphs constructed using Minigraph-Cactus, PGGB, and Minigraph for real-world sorghum assemblies. (A) Number of missing genes across assemblies in each pangenome graph. Minigraph consistently shows the highest number of missing genes, indicating reduced gene representation compared to PGGB and Minigraph-Cactus. (B) Comparison of the number of nodes and edges in the pangenome graphs. PGGB and Minigraph-Cactus have substantially more nodes and edges than Minigraph, reflecting finer-scale graph resolution. (C) Coverage of each input assembly within the pangenome graph. Light blue indicates the portion of the assembly included in the graph, while light green shows the excluded portion.

In addition to simulations, we also constructed pangenome graphs using 6 assemblies representing real-world data. Pangenome graph sizes varied between the 3 pipelines, with the PGGB pangenome reaching 4.7 Gb, the Minigraph-Cactus pangenomes at 4.2 Gb, and the Minigraph pangenomes being considerably smaller at 920 Mb. For comparison, the size of the linear reference genome was 688 Mb, indicating that all pangenomes represented an increase in total genomic content relative to the linear reference, reflecting the added complexity and variation captured by the pangenome graph. The number of nodes and edges in the graph corresponds to its size, as shown in Fig. 4B (Supplementary Table S5a). Size differences between Minigraph and the other 2 graphs are due to the inclusion of only variants >50 bp, while PGGB and Minigraph-Cactus do not have a size filter. When compared to the graph sizes of simulated assemblies, the size and content of real-world pangenomes increase for PGGB and Minigraph-Cactus, consistent with the presence of many additional short variants (SNPs and indels), which were not part of simulations.

The input real-world assemblies had a mean BUSCO completeness of 95.4% with 2% duplication. Pangenomes built with Minigraph-Cactus, PGGB, and Minigraph had BUSCO completeness of 94.5% with 16.5% duplication, 98.7% with 16% duplication, and 99.4% with 2.7% duplication, respectively (Supplementary Table S3a). The reduction in completeness scores is expected, as in some cases, genes will be broken up across multiple nodes. The duplication levels point to a successful genomic data compression, which is one of the key tasks of pangenome graph construction. To address the issue of genes broken up across nodes, which became more pronounced as other species were included in the analysis, we devised a new, *k*-mer–based metric, where *k*-mers derived from BUSCO genes identified in the reference genome were subsequently mapped to the flattened graph FASTA to assess the completeness and duplication levels. The *k*-mer–based analysis painted a similar picture of slightly elevated duplication rates in Minigraph-Cactus and PGGB graphs (Supplementary Table S4).

We tracked the growth and coverage statistics of graphs using Panacus. Growth helps track the accumulation of genes and genomic features, while coverage statistics assess the phylogenetic diversity represented. Overall, all 3 pangenome pipelines show similar graph expansion dynamics, with less nodes added with each new sample (Supplementary Figs. S2–S5).

Pangenome construction can lead to data loss when sequences present in the input assemblies are not included in the pangenome graph. We evaluated the representation of each input assembly within the pangenome graph across all 3 pangenome pipelines. We observed that all assemblies were represented with a coverage above 94% in both the Minigraph-Cactus (Fig. 4A, C) and PGGB (Fig. 4B, C) pangenomes. When assessing gene presence in regions present in the assembly but missing from the graph for each assembly, we found that 1 gene from assembly AusTRCF317961, 4 genes each in IS19953 and IS8525, 14 genes in PI532566, 3 genes in Rio, and 25 genes in S369-1 were not found in the assemblies recovered from the graph (Fig. 4A). These numbers were similar in both Minigraph-Cactus and PGGB pangenomes, but Minigraph showed a comparatively lower proportion (averaging 83%) of assemblies included in the graph (Fig. 4C) and consequently a higher number of missing sequence and genes (Fig. 4A, Supplementary Table S6).

**Figure 5:**
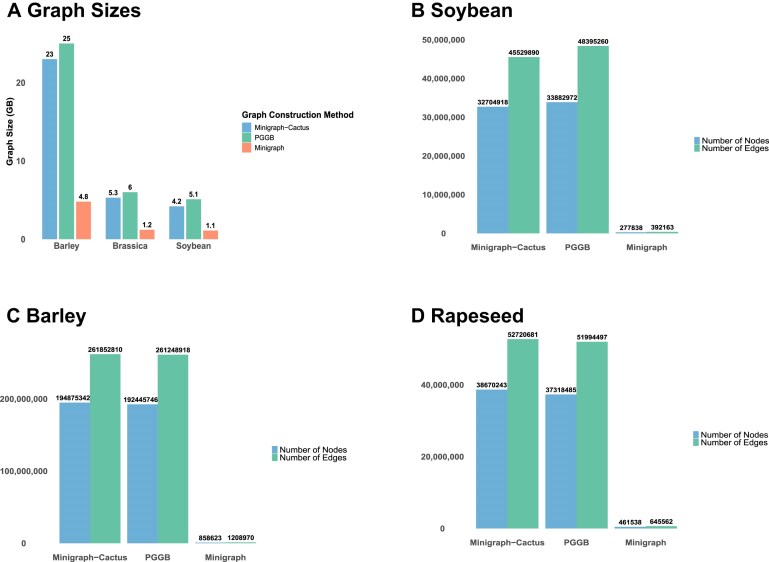
Comparison of (A) graph file sizes and number of nodes and edges for Minigraph-Cactus, PGGB, and Minigraph pangenome graphs constructed from (B) soybean, (C) barley, and (D) rapeseed.

A similar trend was observed in the additional species included in this study. For soybean, rapeseed, and barley, pangenome graphs constructed using PGGB and Minigraph-Cactus were substantially larger than those built with Minigraph, consistent with patterns observed in sorghum. For instance, in soybean, graph sizes were approximately 5.1 Gb (PGGB), 4.2 Gb (Minigraph-Cactus), and 1.1 Gb (Minigraph), with a similar pattern observed for rapeseed and barley (Fig. 5A, Supplementary Table S5). Node count distributions mirrored these differences across species (Fig. 5B–D). Minigraph graph had the highest proportion of sequence and genes missing (Supplementary Table S6). The BUSCO *k*-mer duplication analysis using 21-mers derived from reference BUSCO genes revealed similar patterns as observed in sorghum. The Minigraph-Cactus and PGGB had a higher duplication rate than the Minigraph graph but, overall, showed good ability to reduce the level of sequence redundancy (Supplementary Table S4).

#### Variant calling

We performed a permutation test for variants called from pangenomes (Minigraph-Cactus, PGGB, and Minigraph) constructed with real-world assemblies and also pairwise variant callers (SVIM-asm and SyRI) to compare them with previously reported SVs in sorghum [32]. This test, conducted using the regioneR package, involved generating 100 randomizations of genomic regions to assess the significance of the observed overlaps between pangenome variant sets and the previously reported variant set. All 3 pipelines, Minigraph-Cactus, PGGB, and Minigraph, demonstrated statistically significant results with a permutation count of 100 iterations and randomization using randomizeRegions, yielding a *P* value of 0.0099 and a high *z*-score (Supplementary Fig. S1a–c). These findings indicate that for real-world data, the SVs found using a pangenomic approach overlap previously reported variants much more than would be expected by chance alone. Pairwise variant callers, SVIM-asm and SyRI, also show significant overlap, although to a lesser degree than the pangenome pipelines (Supplementary Fig. S1d, e). Together, with the results from simulations, this suggests that building a pangenome graph using Minigraph-Cactus and PGGB is a viable approach for variant calling.

**Figure 6:**
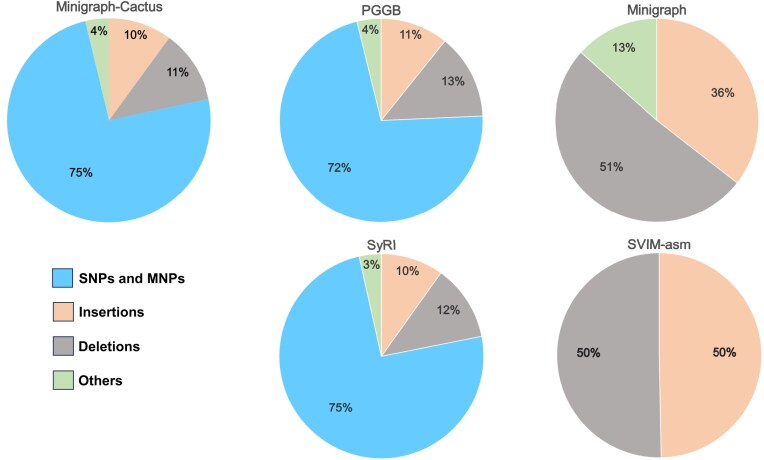
Pie charts comparing the proportion of different variant types called by Minigraph-Cactus, PGGB, Minigraph, SVIM-asm, and SyRI. Minigraph and SVIM-asm focus on structural variants and do not call SNPs or MNPs. The “Others” category includes complex variant types like duplications, inversions, and other structural rearrangements.

We analyzed the proportion of variant types identified by each tool and observed that Minigraph-Cactus, PGGB, and SyRI identify variants of all types and have similar proportions of variant distribution. They also include an “others” category for complex or less common variant types, including duplications, inversions, and other structural rearrangements (Fig. 6).

**Figure 7:**
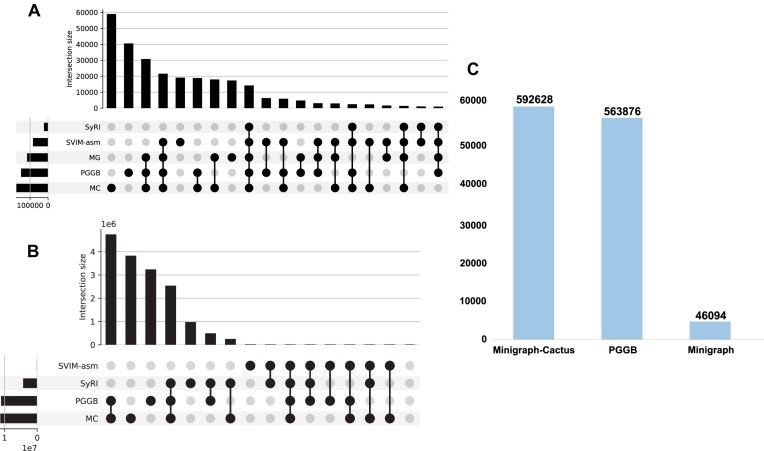
Overlap of variants in real-world assemblies. (A) An UpSet plot showing the overlap of large variants (>50 bp) between different methods; the bars represent the number of shared variants between dataset combinations, with the matrix indicating which datasets are included. (B) An UpSet plot showing the overlap of small variants (<50 bp). (C) Number of multiallelic sites detected by each of the 3 pipelines.

While these results confirm the quality of variants identified by pangenome methods, we also evaluated the overlap of variants between pangenome pipelines and pairwise variant callers (Fig. 7A). Notably, PGGB and Minigraph-Cactus exhibit higher overlap with each other compared to pairwise callers SVIM-asm and Syri. We assessed the performance of all tools in detecting small variants in real-world assemblies, focusing on indels under 50 bp, including multiple-nucleotide polymorphisms (MNPs) and SNPs (Fig. 7B). Minigraph-Cactus detected approximately 7.7 million SNPs and 2.3 million small indels, while PGGB identified around 7.6 million SNPs and 2.6 million small indels. Pairwise variant caller SyRI detected 3.2 million SNPs and 0.93 million small indels, while SVIM-asm detected no SNPs and 11,000 small indels, which is relatively small, resulting in a reasonable overlap between PGGB, Minigraph-Cactus, and SyRI but negligible overlap of variants across all 4 methods (Fig. 7B, Supplementary Table S2). Minigraph-Cactus and PGGB identified 0.59 million and 0.56 million multiallelic sites, respectively, while Minigraph found only 46,000 multiallelic sites (Fig. 7C).

**Figure 8:**
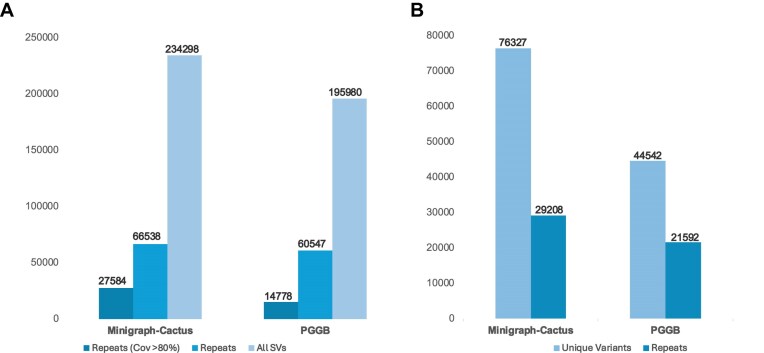
Comparison of SVs with similarity to repeats for Minigraph-Cactus and PGGB. (A) Total counts of structural variations (All SVs) and SVs with similarity to repeats detected by each method, including the count of SVs with similarity to repeats after applying an 80% coverage filter. (B) Count of unique variants identified by Minigraph-Cactus and PGGB and the count of SV with similarity to repeats in those unique variants.

We also analyzed the similarity of variants called by PGGB and Minigraph-Cactus to repeats by comparing them to the *S. bicolor* repeat library. We found that 28% of variants in both PGGB and Minigraph-Cactus matched repeats. When only matches with over 80% coverage were considered, these results were reduced to 7.5% for PGGB variants and 11% match for Minigraph-Cactus variants (Fig. 8A). Both Minigraph-Cactus and PGGB called a high number of variants unique to each of the pipelines (Fig. 8B). We also analyzed repeat similarity of those unique variants and found that 48% of PGGB unique variants and 38% of Minigraph-Cactus unique variants matched with repeat locations (Fig. 8B).

For the additional species, soybean, rapeseed, and barley, we performed variant calling for the same pangenome pipelines (Minigraph-Cactus, PGGB, and Minigraph) on real-world assemblies, along with SVIM-asm and SyRI. We quantified the total number and types of variants identified by each pipeline to provide a comparative overview of variant detection across species (Supplementary Table S2, Supplementary Figs. S6–S8). Variant counts ranged, with a similar distribution of SNPs, small indels, and multiallelic sites observed as in *S. bicolor* (Supplementary Table S2).

#### Read mapping

One of the key objectives of a pangenome graph is to act as a reference for read mapping. We therefore assessed mapping outcomes for graphs produced with different pipelines. We were unable to generate mapping indexes for the PGGB pangenome graph due to heavy computational and memory requirements and hence were unable to test it for read mapping. This issue has been previously reported during chicken pangenome analysis [51]. Following the approach of Rice et al. [51], we evaluated the performance of linear and graph-based mapping by simulating reads from the entire pangenome graph, which includes all 7 assemblies (6 input assemblies and the *S. bicolor* v3.0.1 reference), rather than just from a single reference genome. The simulated reads were then mapped to both the graph using VG Giraffe and the *S. bicolor* v3.0.1 linear reference using Minimap2. This allowed us to assess the mapping performance across the genetic diversity captured in the pangenome. For Minigraph-Cactus, the Giraffe results show better mapping compared to the corresponding Minimap2 alignments (Supplementary Table S1a). Specifically, at a high minimum mapping quality (min_mapQ) threshold of 60, although Giraffe (Minigraph-Cactus) only maps 38.27%, it achieves a correct read mapping rate of 99.68%, substantially better than Minimap2’s, which maps 48.67% reads with only 58.18% reads mapped correctly (Supplementary Table S1a). Correctly mapped reads are simulated reads that align back to their original genomic position from where they were simulated. Giraffe (Minigraph-Cactus) maintains a high rate (over 98%) of correctly mapped reads, whereas Minimap2 has a lower proportion of correctly mapped reads at similar thresholds (Supplementary Table S1a). At min_mapQ 1, the percentage of total reads aligned correctly is 79.60%, when compared to Minimap2’s 35.49% (Supplementary Table S1a). This demonstrates that Giraffe using the Minigraph-Cactus pangenome offers substantial improvements in both mapping accuracy and total alignment correctness compared to linear-based approaches. It is notable that despite simulating the reads from the same pangenome graph, some reads did not align to the graph at mapQ 1, which likely reflects alignment ambiguity in highly repetitive regions as the mapQ 1 threshold may exclude low-confidence alignments, contributing to the reduced alignment rate.

Giraffe performs well also with the Minigraph pangenome. At a min_mapQ threshold of 60, Giraffe (Minigraph) maps 74.62% of reads with a 95.08% correct read mapping rate, which is better than the corresponding Minimap2’s mapping of 58.93% reads with a 92.78% correct read mapping rate. The correctness rate for Giraffe (Minigraph) remains high at around 98%, similar to the results with Minigraph-Cactus. Furthermore, the percentage of total reads aligned correctly with Giraffe (Minigraph) reached 91.91% at min_mapQ 1, when compared to Minimap2’s 65.74% (Supplementary Table S1a), highlighting its superior mapping ability compared to both Minigraph-Cactus and the linear reference. The comparatively good read mapping performance of the Minigraph-only graph can be attributed to much lower graph complexity due to the absence of small variants, which makes read simulation and the mapping task much more straightforward. Minigraph-Cactus builds more complex graphs, capturing a wider range of genetic variations. While this detailed approach provides a richer genomic view, it also makes the read mapping process slower and more complex.

**Figure 9:**
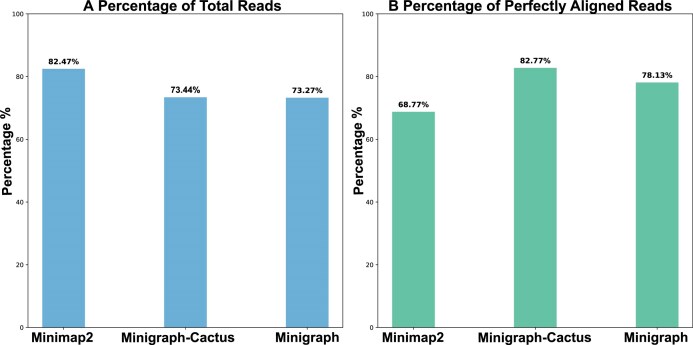
Comparison of read alignment metrics for Minigraph-Cactus, Minigraph, and Minimap2 for real-world reads. (A) The percentage of total reads successfully aligned by each tool: Minimap2 achieved the highest alignment rate at 82.47%, followed by Minigraph-Cactus with 73.44% and Minigraph with 73.27%. (B) The percentage of perfectly aligned reads, where Minigraph-Cactus outperformed the others with an alignment rate of 82.77%, Minigraph had 78.13%, and Minimap2 only achieved 68.77%, highlighting the lower quality of read alignments to the linear reference (Minimap2) in (A) when compared to pangenome graph references (Minigraph-Cactus and Minigraph).

We tested the read mapping ability of both pangenomes and a linear reference in the case of real-world reads as well, and the results showed the excellent ability of pangenomes to map real-world reads [32]. Minigraph-Cactus aligns 73.44% of total reads (Fig. 9A), out of which 82.77% of reads are aligned perfectly (Fig. 9B). On the other hand, Minigraph aligns 73.27% of total reads (Fig. 9A), out of which 78.13% are aligned perfectly (Fig. 9B). In contrast, Minimap2 aligns 82.47% of total reads (Fig. 9A), out of which only 68.77% are aligned perfectly (Fig. 9B).

When comparing mapping ability of pangenomes to linear references, it is evident that pangenomes consistently offer superior alignment performance. Giraffe’s alignment to both the Minigraph-Cactus and Minigraph pangenomes resulted in higher percentages of correctly mapped reads and total correct alignments compared to Minimap2’s alignment to linear references.

We applied the same read simulation and mapping evaluation strategy to additional crop species, soybean, rapeseed, and barley, and their pangenome graphs constructed with Minigraph-Cactus and Minigraph. As observed in sorghum, mapping with VG Giraffe to the pangenome graphs consistently outperformed alignment with Minimap2 to the linear reference genomes, in terms of both total mapped reads and correctly mapped reads across all species (Supplementary Table S1a). In addition to simulated reads, we also mapped publicly available real-world reads. Across all datasets, graph-based alignment consistently yielded a higher proportion of perfectly mapped reads than linear reference alignment (Supplementary Table S1b).

## Discussion

Previous benchmarking efforts focused mainly on animal genomes [42, 51, 57], which, compared to plants, can have very different properties, including much higher levels of intra- and interspecies collinearity [58]. Assessing the methods’ performance with crop data is therefore of high importance. This study offers a comparison of 3 key pangenome assembly pipelines (Minigraph-Cactus, PGGB, and Minigraph) using sorghum as a model, highlighting how each approach handles the complexity of pangenome construction, content, and performance in variant calling and mapping. One of the key tasks of pangenome graph construction pipelines is an effective identification of regions of sequence correspondence and variation from whole-genome alignments. We compared performance of the state-of-the-art pangenome pipelines (Minigraph-Cactus, PGGB, and Minigraph) with established pipelines for SV identification from pairwise genome alignments (SVIM-asm and SyRI). To thoroughly evaluate the performance of the SV calling pipelines, we used both simulated and real-world datasets. The simulated data, based on a reference backbone and containing known SVs, provided a controlled environment where we could assess variant calling accuracy with a known true set of SVs. In contrast, the real-world data, which include inherent biological complexities such as sequencing errors and structural variation, allowed us to test the pipelines under more challenging and realistic conditions. This dual approach ensured a comprehensive evaluation of each pipeline’s performance across both ideal and complex scenarios.

While the pairwise variant callers (SVIM-asm and SyRI) had slightly higher F1 scores in the simulations compared to the top-performing pangenome graph construction pipeline (Minigraph-Cactus), overall, pangenome graph-based methods showed strong performance in simulations and good alignment with previously reported variation in real-world datasets. We speculate that the performance of pairwise variant callers could be inflated in simulations due to oversimplified scenarios, without small or multiallelelic variants. While the complexity of Minigraph-Cactus– and PGGB-generated graphs leads to substantial disparity in the number of variants detected by the two pipelines, they both capture a large number of variants, which appear to have been missed by the pairwise variant callers in real-world scenarios. Pangenome graph approaches appear effective in identifying variants across multiple assemblies and capture both common and rare variants. Pangenome graph construction pipelines therefore emerge as a robust solution when dealing with complex, large datasets in a real-world scenario.

Graph-based pangenomes have been increasingly recognized as a superior approach compared to *de novo* or iterative methods, as they provide a unified framework to represent complex variation and multiple haplotypes simultaneously. Graph pangenomes have the capacity to improve variant discovery and read mapping accuracy by reducing reference bias and better capturing structural diversity [51, 59]. For example, Vaughn et al. [60] and Lemay et al. [61] demonstrated that graph-based approaches enable accurate genotyping of variants across large populations. By capturing complex allelic variation and sequence context often missed by linear reference–based methods, it allows for a more comprehensive representation of population diversity. Our results align with these findings, reinforcing the value of graph-based approaches for comprehensive and scalable pangenome construction in diverse plant species.

To evaluate whether the trends observed in *S. bicolor* are consistent across diverse plant genomes, we extended key analyses to 3 additional crops: *G. max* (soybean), *B. napus* (canola), and *H. vulgare* (barley). Using real-world assemblies, we applied the same pangenome construction and variant calling pipelines, focusing on graph sizes, completeness, duplication levels, and variant detection. Pangenome graph sizes and duplication rates followed similar patterns across methods, and the low duplication levels observed in *k*-mer–based analyses indicate good compressibility of the pangenomes, despite genome complexity. These cross-species analyses highlight the general applicability and robustness of the pipelines for plant pangenome construction and variant discovery, underscoring their potential utility across crops with varying genome architectures.

The study revealed that pangenome-based approaches generally offer better alignment accuracy compared to traditional linear references for mapping performance. Giraffe, used with both Minigraph-Cactus and Minigraph pangenomes, consistently outperformed Minimap2 in correctly mapping reads, underscoring the advantages of graph-based methods for managing complex genomic data. However, PGGB’s computational demands were a significant challenge, especially in generating mapping indexes, indicating the need for further optimization.

The identification of genetic variation through pangenome methods becomes particularly challenging when dealing with highly complex variations, such as nested or tandemly repeated transposable element insertions [62]. Recent analysis in rice demonstrates that the Minigraph-Cactus pipeline can accurately identify 5 different alleles of a complex locus consisting of nested and tandemly repeated transposon insertions within a pangenome of 20 different accessions of *O. sativa*. Compared to a linear pangenome analysis, the pangenome graph approach allowed for improved characterization of multiple related alleles. However, correct genotyping of multiple alleles in a large population using short reads remained challenging.

In this study, we constructed pangenomes for each chromosome separately, as this approach offers several advantages, including reduced processing time, simplified data management, and easier identification of variations within individual chromosomes. However, it does not account for the connections between chromosomes. To investigate complex chromosomal changes, such as Robertsonian translocations or centromeric homology [57], building a whole-genome pangenome may be essential.

Our findings emphasize the importance of selecting tools aligned with specific research objectives, whether prioritizing variant discovery, optimizing computational resources, or ensuring high mapping accuracy. PGGB is designed as a completely reference-free approach relying on all-versus-all alignments, but this appears to result in a potential trade-off in precision and recall of SV identification. Minigraph-Cactus progressively builds a graph and appears to offer a balanced integration of new sequences with robust performance. Minigraph, though efficient for identifying large structural variants, will miss finer variant details, particularly in highly diverse regions. Despite the advantages of pangenome-based approaches in capturing both common and rare variants across multiple assemblies, this study also highlights that these methods still require improvements. Additionally, refining pangenome assembly tools to address the unique complexities of plant genomes will yield more accurate and comprehensive genomic analyses.

## Availability of Source Code and Requirements

Project name: Benchmarking_graph_pipelines

Project homepage: https://github.com/KopalliV/Benchmarking_graph_pipelines

License: GPL-3.0 license

SciCrunch RRID:SCR_026567

bio.tools ID: benchmarking_graph_pipelines

System requirements

Operating system: Linux

Programming language: Bash, R, and Python

Package management: Conda/bioconda, pip

Hardware requirements: HPC environment with ≥32 CPU cores, ≥128 GB RAM, and ~2 TB storage

## Supplementary Material

giaf121_Supplemental_Files

giaf121_Authors_Response_To_Reviewer_Comments_Original_Submission

giaf121_GIGA-D-25-00090_Original_Submission

giaf121_GIGA-D-25-00090_Revision_1

giaf121_Reviewer_1_Report_Original_Submission Kapeel M. Chougule -- 4/14/2025

giaf121_Reviewer_1_Report_Revision_1 Kapeel M. Chougule -- 9/3/2025

giaf121_Reviewer_2_Report_Original_Submission Yongfeng Zhou -- 4/15/2025

giaf121_Reviewer_2_Report_Revision_1 Yongfeng Zhou -- 9/3/2025

## Data Availability

The sorghum genome assemblies used in this study were reused from the CNGB Nucleotide Sequence Archive project [63]. Other data further supporting this work are openly available in the *GigaScience* repository, GigaDB [64].

## Additional Files

**Supplementary Fig. S1**. Permutation test results for real-world assemblies using Minigraph-Cactus, PGGG, and Minigraph pipelines. This figure compares variants identified with previously reported variants. Using the regioneR package, 100 randomizations were performed. In all the plots, the observed overlap (red line) deviates significantly from expected (black line) with a *P* value of 0.0099 and very high *z*-score. (a) Minigraph-Cactus. (b) PGGB. (c) Minigraph. (d) SVIM-asm. (e) SyRI.

**Supplementary Fig. S2**. Comparison of genome graph construction methods, (a) Minigraph-Cactus, (b) PGGB, and (c) Minigraph, using Panacus. The left panel shows coverage histograms, showing the distribution of node coverage across different levels. The middle panel shows the growth of the total number of nodes as more samples are added, with different coverage and quorum thresholds represented by various colors. The right panel shows the Fnew plot, which tracks the number of new nodes introduced as sample size increases.

**Supplementary Fig. S3**. Comparison of genome graph construction methods of barley pangenomes, (a) Minigraph-Cactus, (b) PGGB, and (c) Minigraph, using Panacus. The left panel shows coverage histograms, showing the distribution of node coverage across different levels. The middle panel shows the growth of the total number of nodes as more samples are added, with different coverage and quorum thresholds represented by various colors. The right panel shows the Fnew plot, which tracks the number of new nodes introduced as sample size increases.

**Supplementary Fig. S4**. Comparison of genome graph construction methods of soybean pangenomes, (a) Minigraph-Cactus, (b) PGGB, and (c) Minigraph, using Panacus. The left panel shows coverage histograms, showing the distribution of node coverage across different levels. The middle panel shows the growth of the total number of nodes as more samples are added, with different coverage and quorum thresholds represented by various colors. The right panel shows the Fnew plot, which tracks the number of new nodes introduced as sample size increases.

**Supplementary Fig. S5**. Comparison of genome graph construction methods of rapeseed pangenomes, (a) Minigraph-Cactus, (b) PGGB, and (c) Minigraph, using Panacus. The left panel shows coverage histograms, showing the distribution of node coverage across different levels. The middle panel shows the growth of the total number of nodes as more samples are added, with different coverage and quorum thresholds represented by various colors. The right panel shows the Fnew plot, which tracks the number of new nodes introduced as sample size increases.

**Supplementary Fig. S6**. Overlap of variants in real-world assemblies of soybean. (a) An UpSet plot showing the overlap of large variants (>50 bp) between different methods; the bars represent the number of shared variants between dataset combinations, with the matrix indicating which datasets are included. (b) An UpSet plot showing the overlap of small variants (<50 bp).

**Supplementary Fig. S7**. Overlap of variants in real-world assemblies of rapeseed. (a) An UpSet plot showing the overlap of large variants (>50 bp) between different methods; the bars represent the number of shared variants between dataset combinations, with the matrix indicating which datasets are included. (b) An UpSet plot showing the overlap of small variants (<50 bp).

**Supplementary Fig. S8**. Overlap of variants in real-world assemblies of barley. (a) An UpSet plot showing the overlap of large variants (>50 bp) between different methods; the bars represent the number of shared variants between dataset combinations, with the matrix indicating which datasets are included. (b) An UpSet plot showing the overlap of small variants (<50 bp).

**Supplementary Table S1**. Read mapping statistics.

**Supplementary Table S2**. Variant statistics observed in all 4 crops across 3 pangenome graph pipelines and 2 pairwise variant callers.

**Supplementary Table S3**. BUSCO scores of flattened pangenome graphs built with real-world and simulated assemblies of sorghum.

**Supplementary Table S4**. The *k*-mer mapping statistics for the linear reference and flattened graph FASTAs.

**Supplementary Table S5**. Graph statistics and runtimes for all 4 crops across 3 pangenome graph construction pipelines.

**Supplementary Table S6**. The number of unmapped genes and genome coverage percentages per assembly path across 4 crop species and 3 pangenome construction methods.

**Supplementary Table S7**. Data sources and assemblies used in the study for rapeseed, soybean, and barley.

## Abbreviations

BUSCO: Benchmarking Universal Single-Copy Orthologs; CDS: coding sequence; CNV: copy number variation; HDR: highly diverged region; indel: insertion/deletion; MNP: multiple-nucleotide polymorphism; PAV: presence/absence variant; PGGB: PanGenome Graph Builder; SNP: single-nucleotide polymorphism; SV: structural variation; TE: transposable element; WGA: whole-genome assembly.
