# Supplementary material for: Toward a standardized framework for pangenome graph evaluation: assessing crop plant pangenome variation graph construction from multiple assemblies
Source: Gigascience. 2025 Dec 4;14:giaf121. doi: 10.1093/gigascience/giaf121 (PMC12676463; doi:10.1093/gigascience/giaf121)
Supplement: giaf121_Supplemental_Files [file giaf121_supplemental_files.zip › Supplementary_data_08082025.docx]

**c) Minigraph**


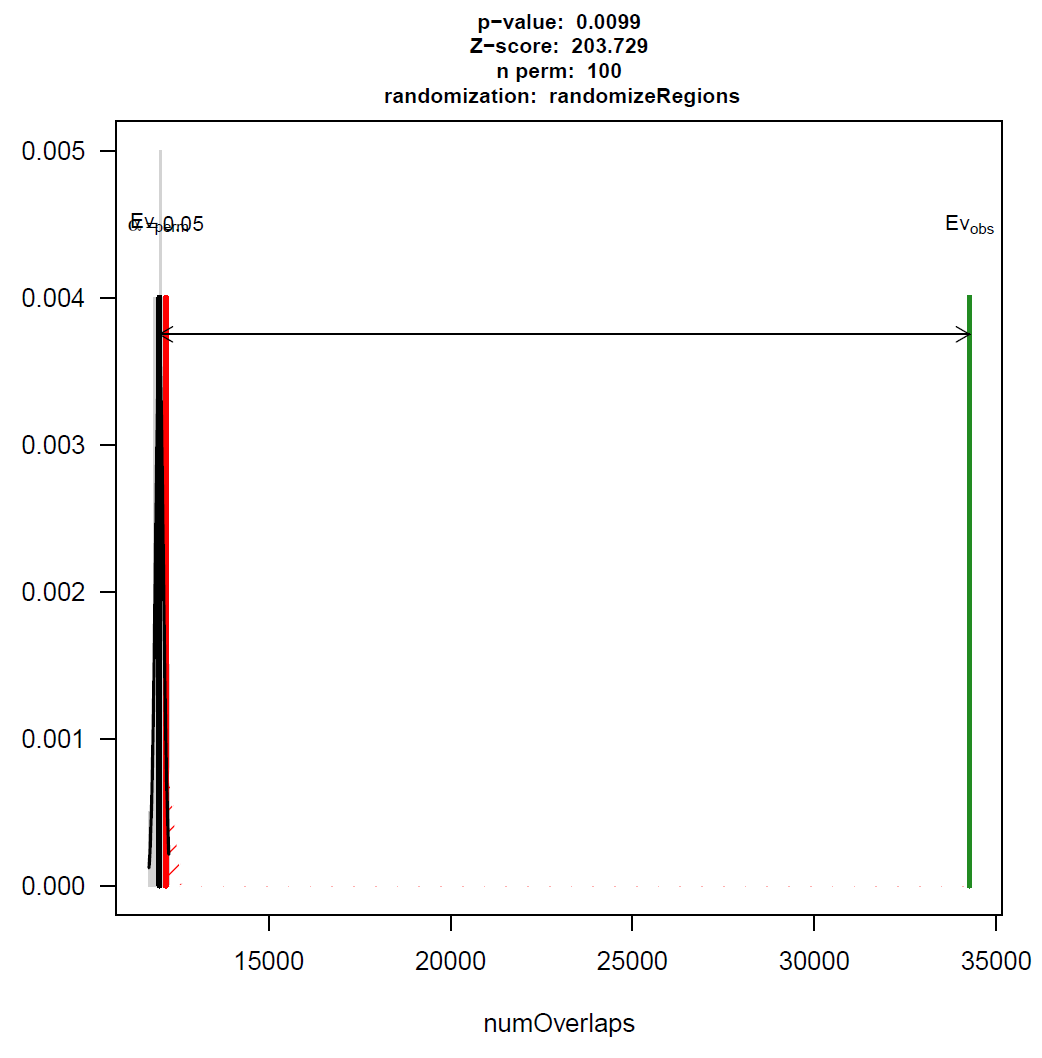

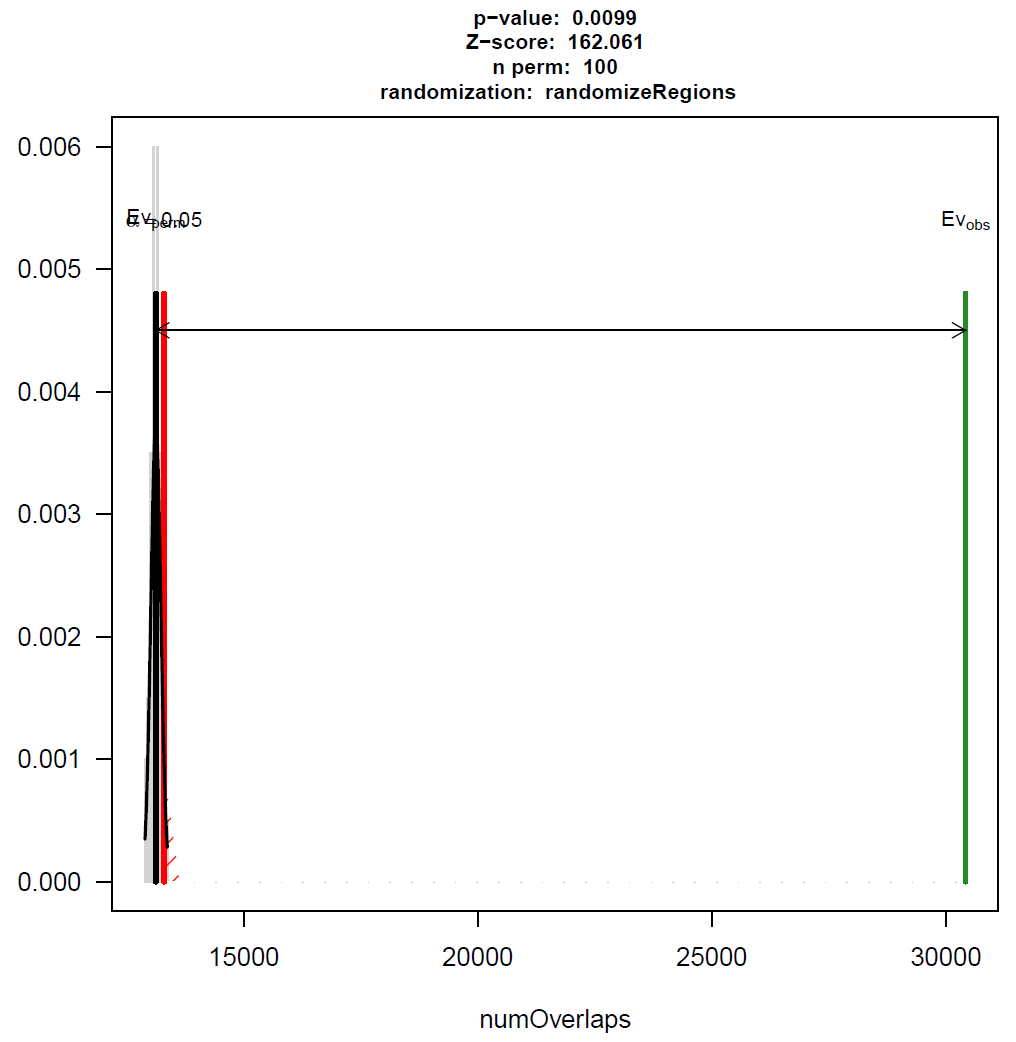

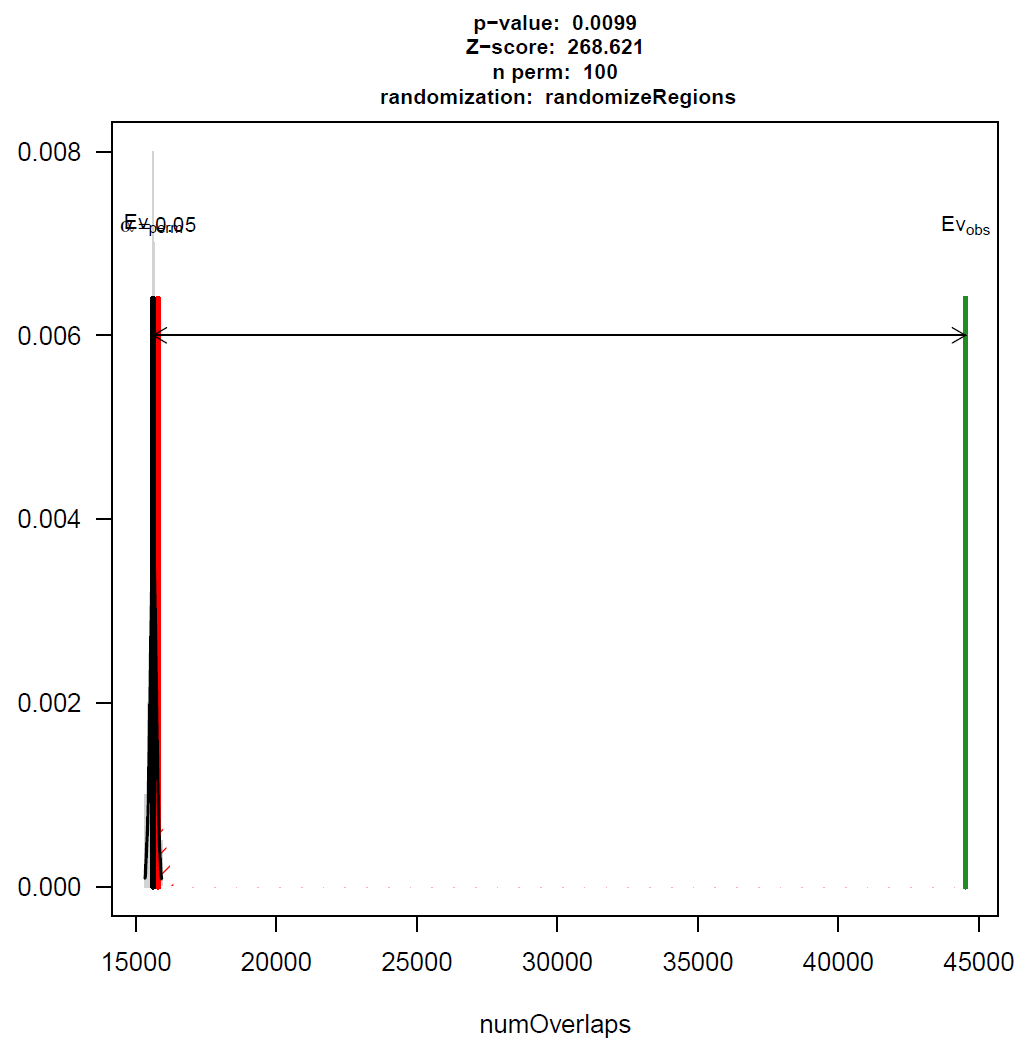


**b) PGGB**

**a) Minigraph-Cactus**

**e) SyRI**

**d) SVIM-asm**


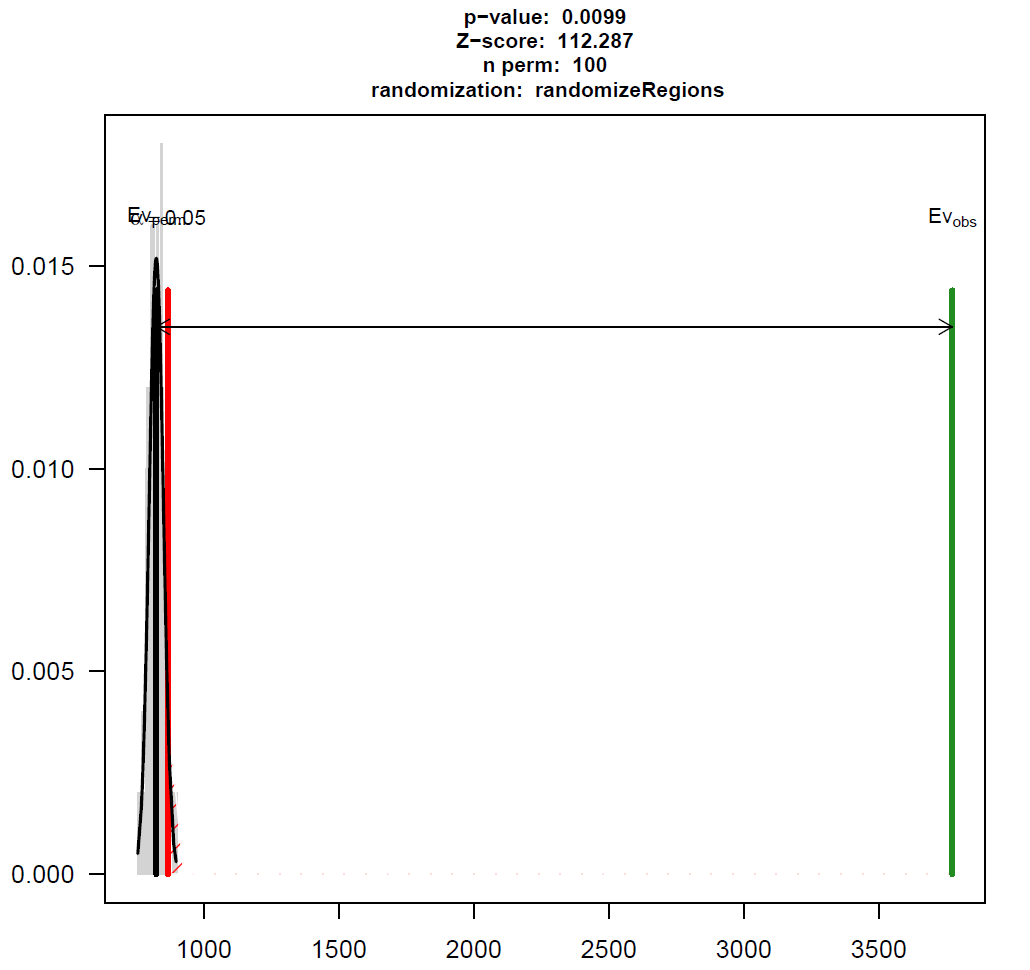

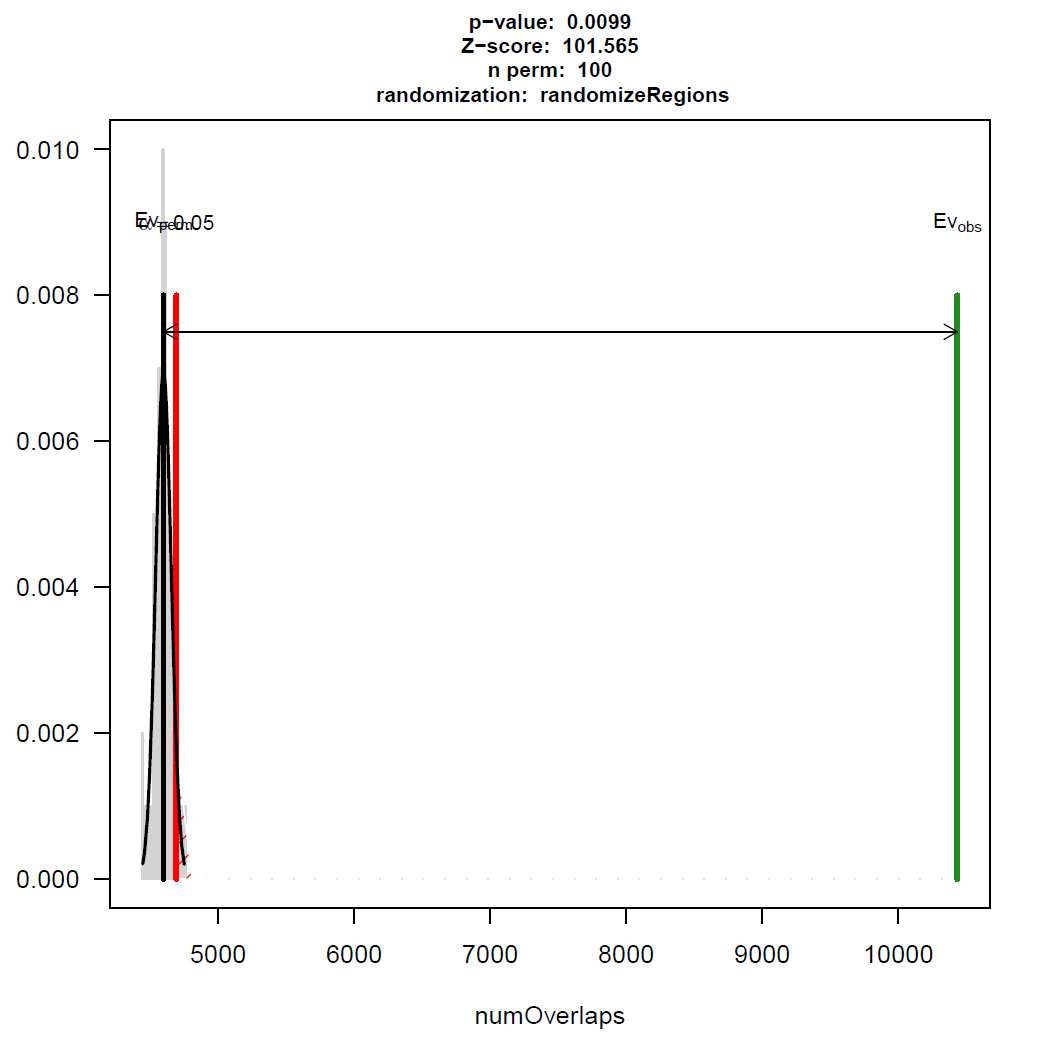


**Supplementary Figure 1:** Permutation test results for real-world assemblies using Minigraph-Cactus, PGGG and Minigraph pipelines*.* This figure compares variants identified agins previously reported varaints. Using the regioneR package, 100 randomizations were performed. In all the plots, the observed overlap (red line) deviates significantly from expected (black line) with a p-value of 0.0099 a very high Z-score. a) Minigraph-Cactus, b) PGGB, c) Minigraph, d) SVIM-asm, e) SyRI.


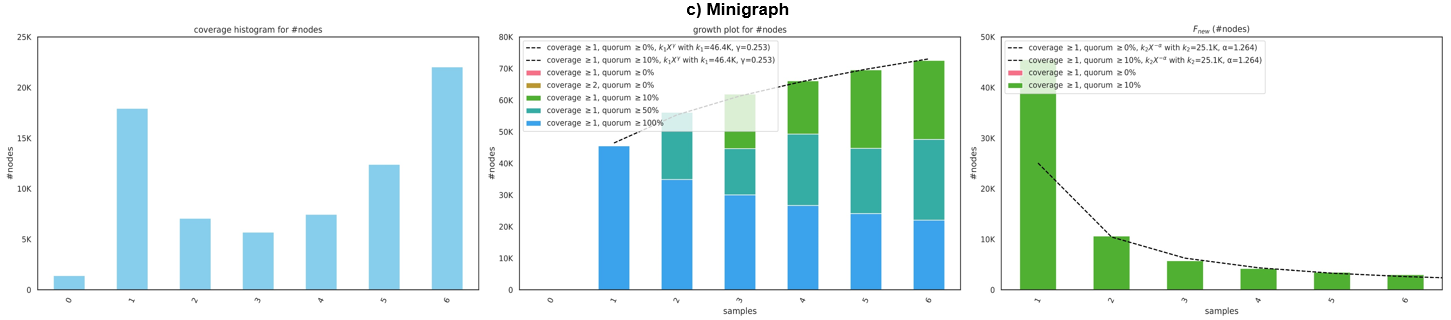

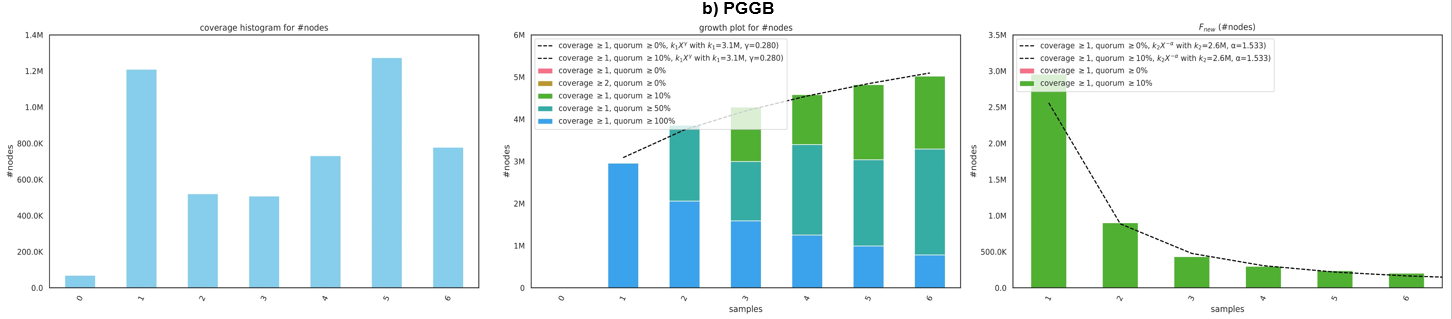

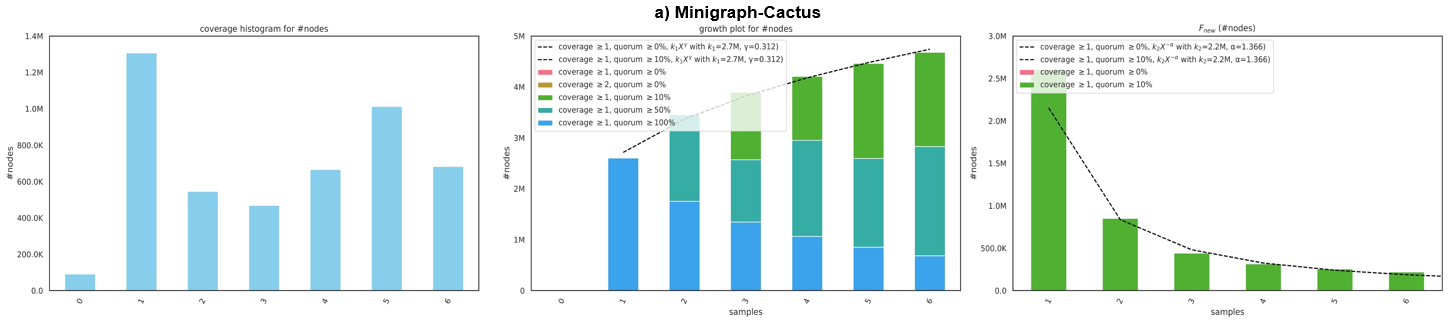


**Supplementary Figure 2:** Comparison of genome graph construction methods, (a) Minigraph-Cactus, (b) PGGB, and (c) Minigraph, using Panacus. The left panel shows coverage histograms, showing the distribution of node coverage across different levels. The middle panel shows the growth of the total number of nodes as more samples are added, with different coverage and quorum thresholds represented by various colours. The right panel shows the Fnew plot, which tracks the number of new nodes introduced as sample size increases.


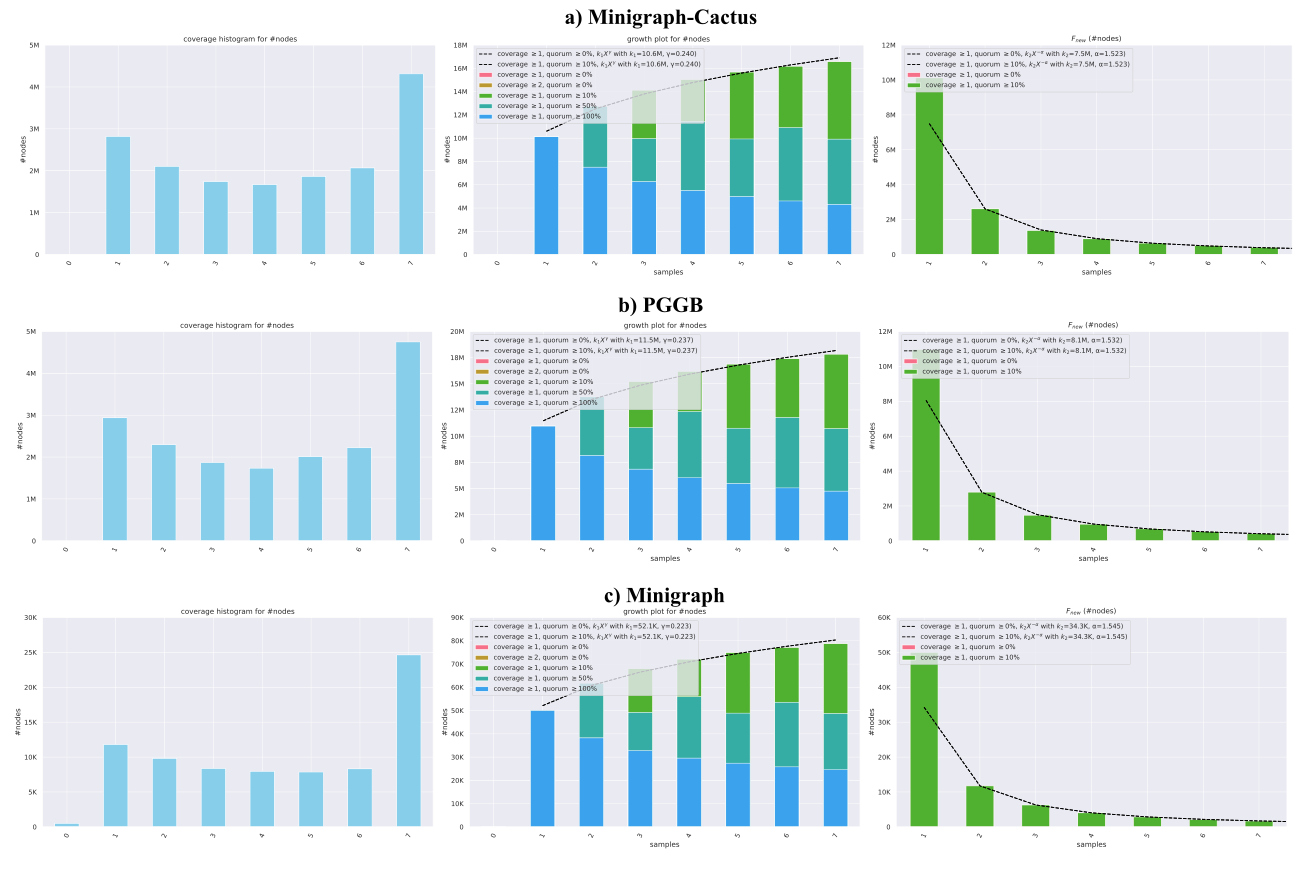


**Supplementary Figure 3**: Comparison of genome graph construction methods of Barley pangenomes, (a) Minigraph-Cactus, (b) PGGB, and (c) Minigraph, using Panacus. The left panel shows coverage histograms, showing the distribution of node coverage across different levels. The middle panel shows the growth of the total number of nodes as more samples are added, with different coverage and quorum thresholds represented by various colours. The right panel shows the Fnew plot, which tracks the number of new nodes introduced as sample size increases.


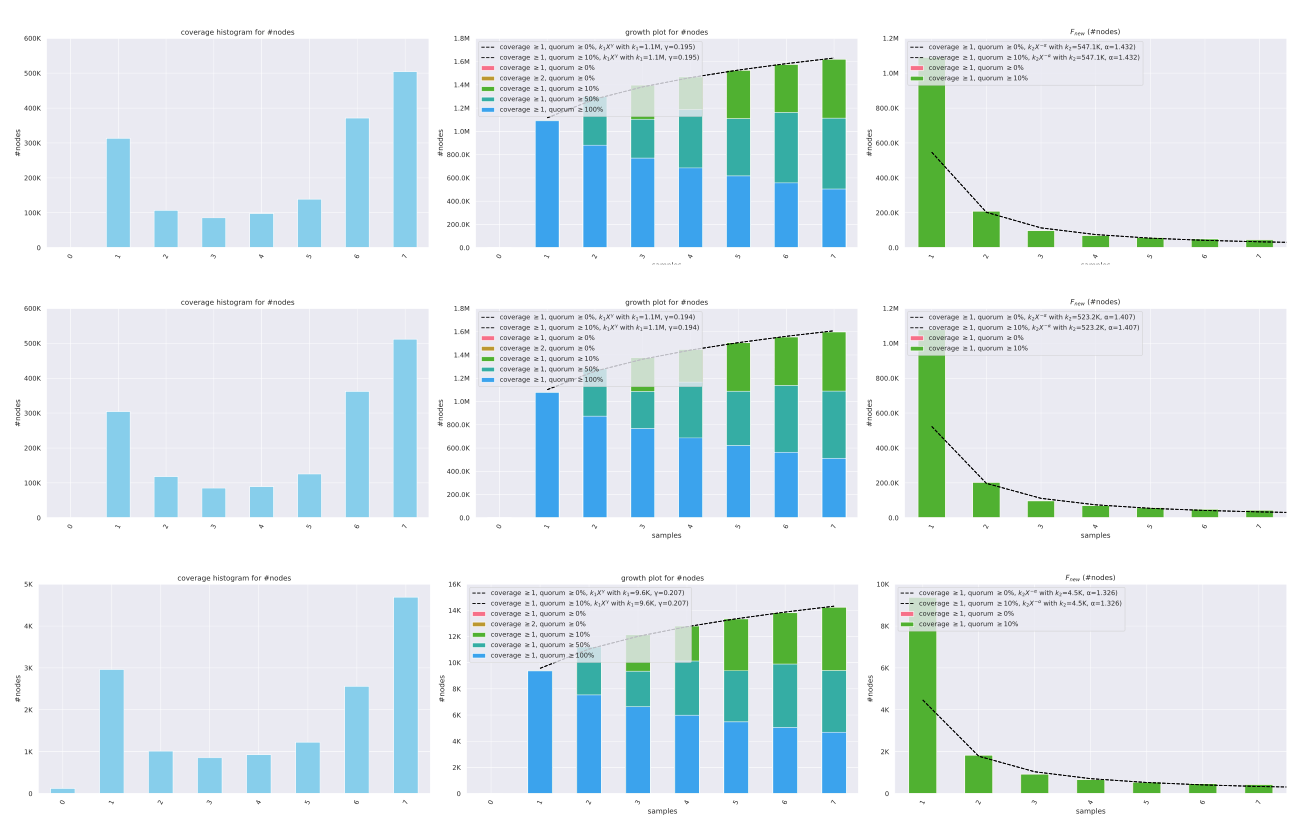


1. **Minigraph**
2. **PGGB**
3. **Minigraph-Cactus**

**Supplementary Figure 4**: Comparison of genome graph construction methods of Soybean pangenomes, (a) Minigraph-Cactus, (b) PGGB, and (c) Minigraph, using Panacus. The left panel shows coverage histograms, showing the distribution of node coverage across different levels. The middle panel shows the growth of the total number of nodes as more samples are added, with different coverage and quorum thresholds represented by various colours. The right panel shows the Fnew plot, which tracks the number of new nodes introduced as sample size increases.

1. **Minigraph**
2. **PGGB**
3. **Minigraph-Cactus**


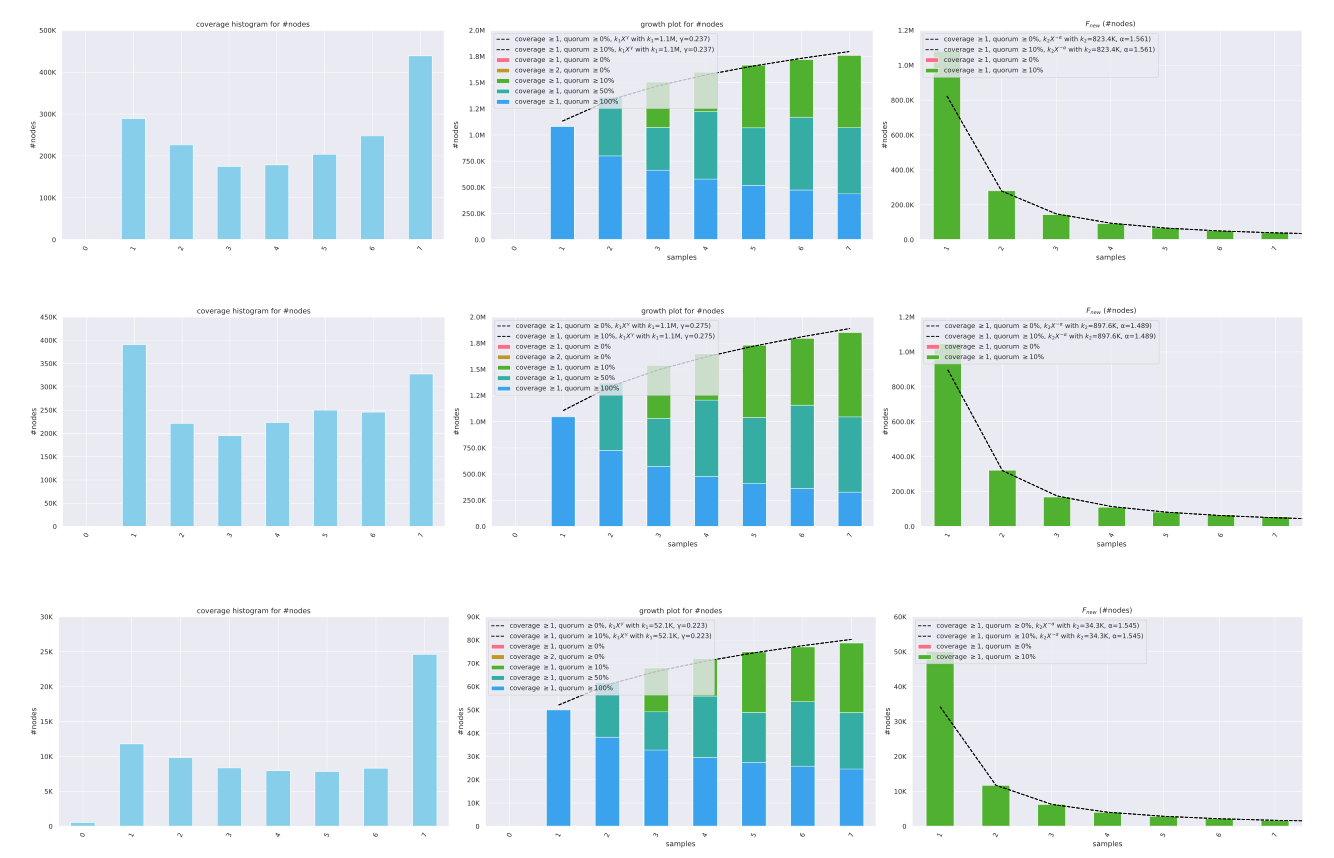


**Supplementary Figure 5**: Comparison of genome graph construction methods of Rapeseed pangenomes, (a) Minigraph-Cactus, (b) PGGB, and (c) Minigraph, using Panacus. The left panel shows coverage histograms, showing the distribution of node coverage across different levels. The middle panel shows the growth of the total number of nodes as more samples are added, with different coverage and quorum thresholds represented by various colours. The right panel shows the Fnew plot, which tracks the number of new nodes introduced as sample size increases.


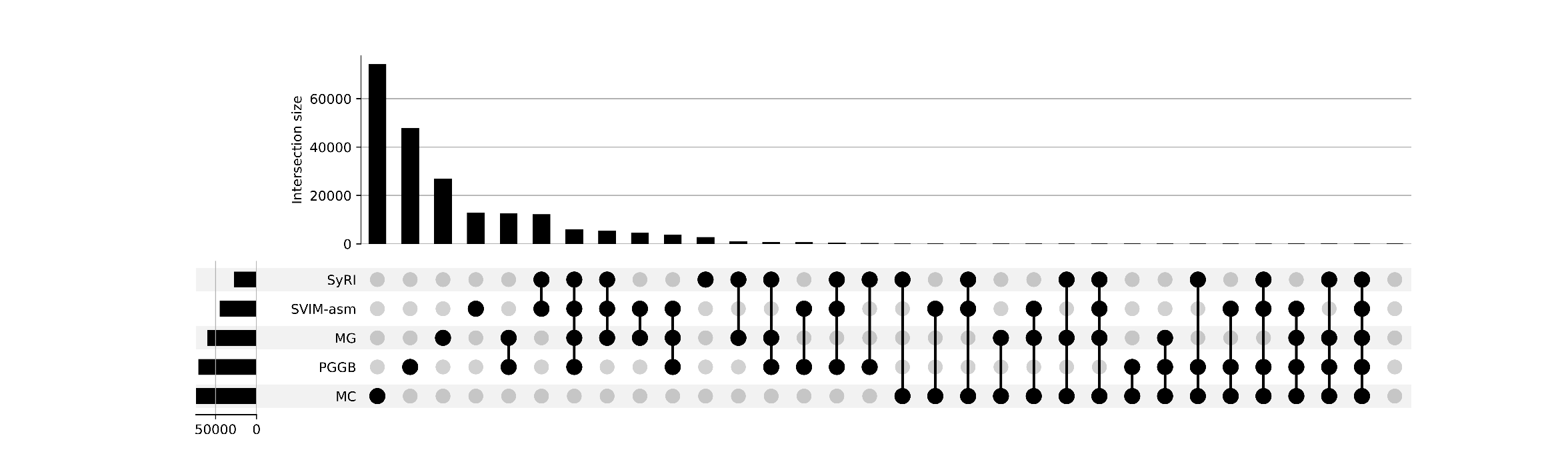


**a)**


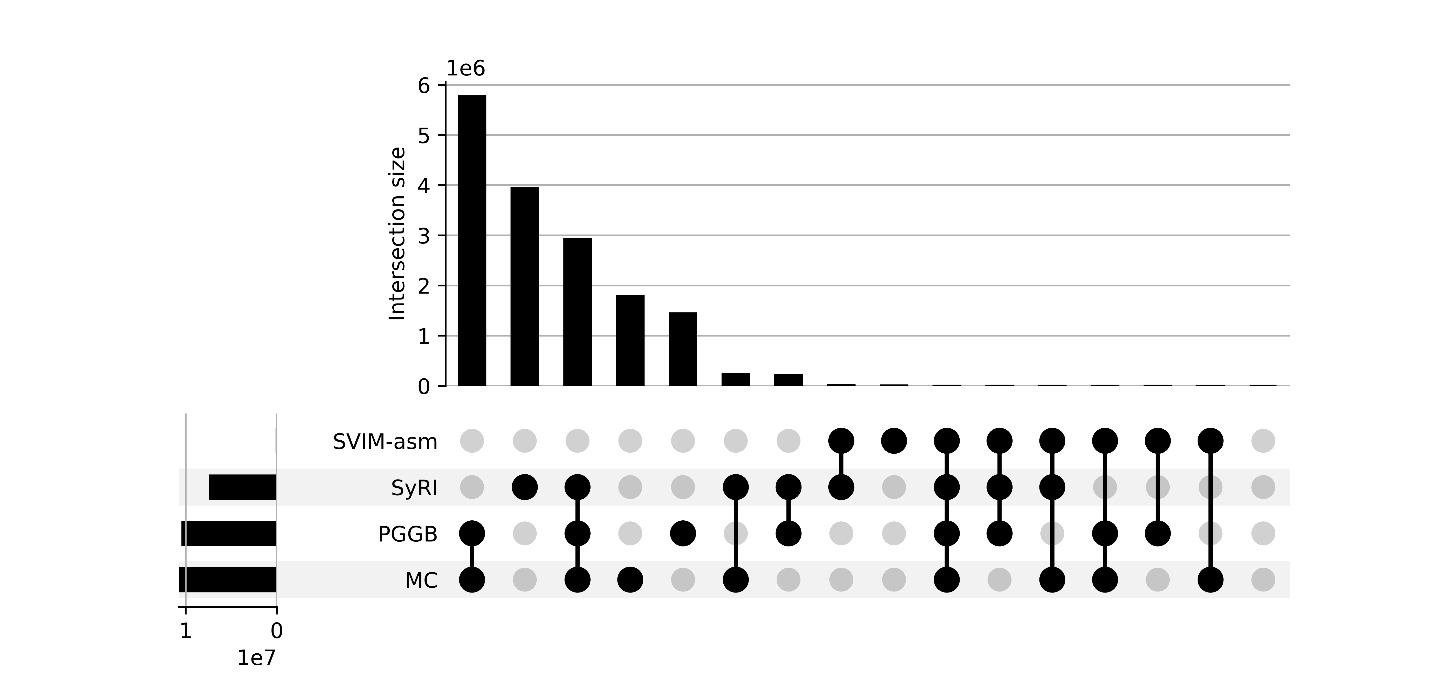


**b)**

**Supplementary Figure 6:** Overlap of variants in real-world assemblies of Soybean. a) An UpSet plot showing the overlap of large variants (>50bp) between different methods, the bars represent the number of shared variants between dataset combinations, with the matrix below indicating which datasets are included. b) An UpSet plot showing the overlap of small variants (<50bp).


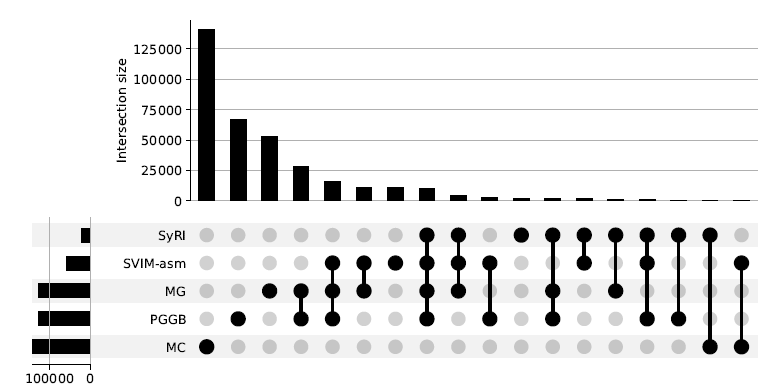


**a)**


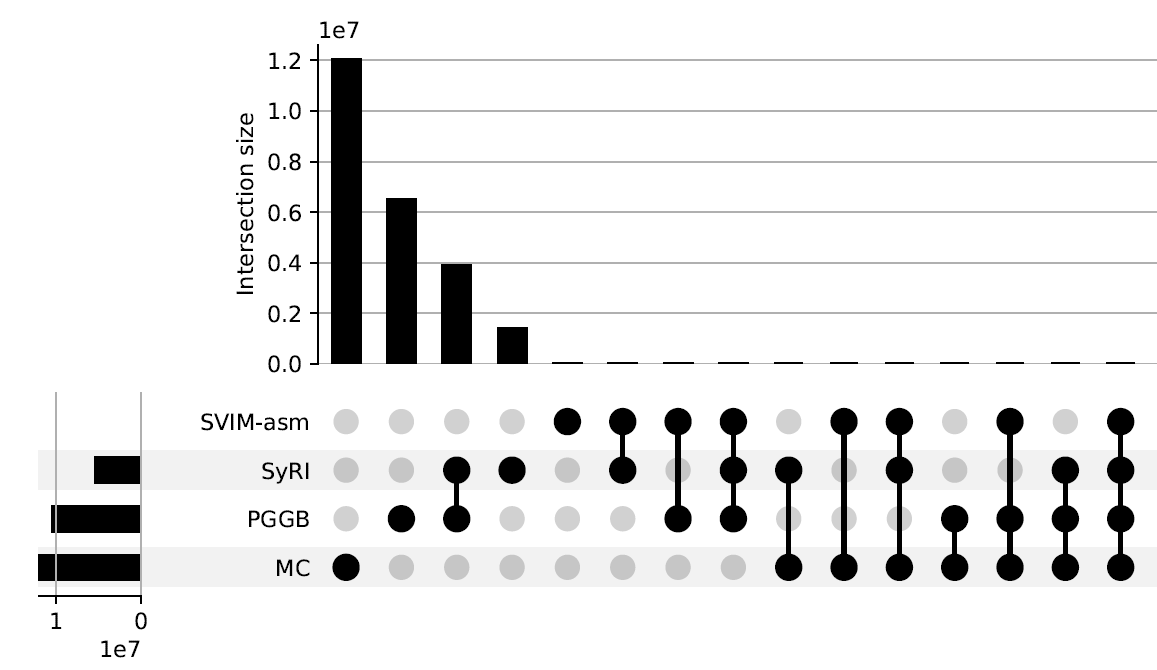


**b)**

**Supplementary Figure 7:** Overlap of variants in real-world assemblies of Rapeseed. a) An UpSet plot showing the overlap of large variants (>50bp) between different methods, the bars represent the number of shared variants between dataset combinations, with the matrix below indicating which datasets are included. b) An UpSet plot showing the overlap of small variants (<50bp).


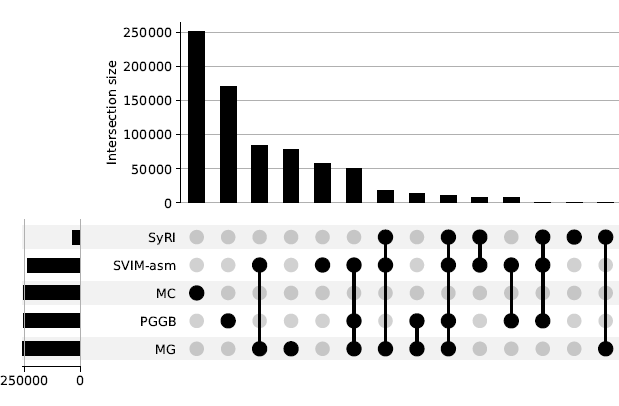


**a)**


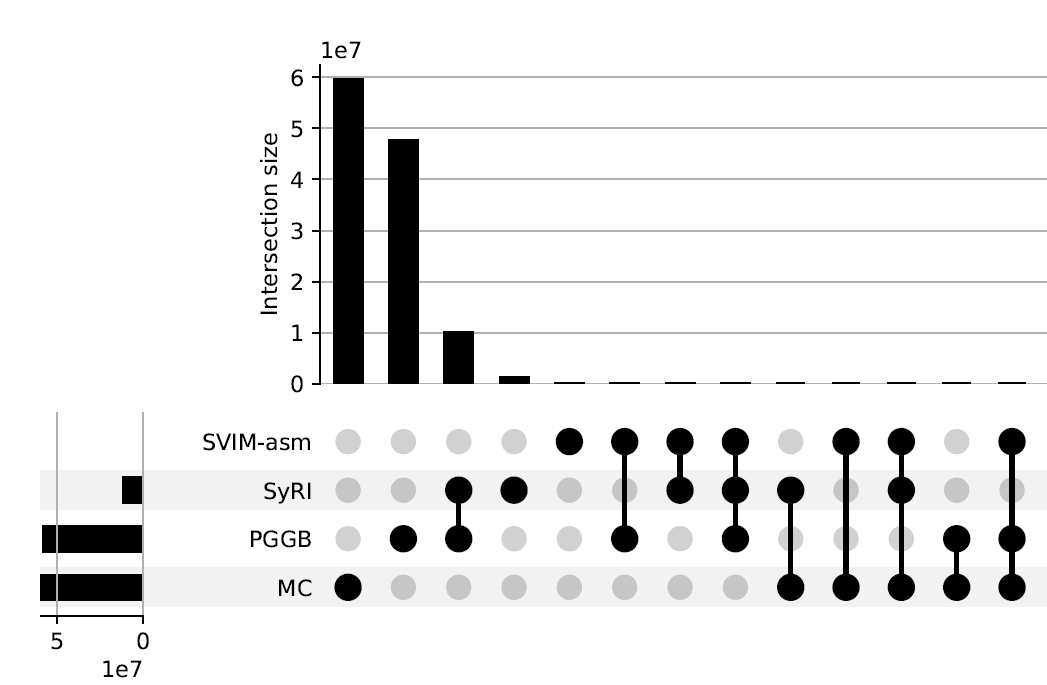


**b)**

**Supplementary Figure 8:** Overlap of variants in real-world assemblies of Barley. a) An UpSet plot showing the overlap of large variants (>50bp) between different methods, the bars represent the number of shared variants between dataset combinations, with the matrix below indicating which datasets are included. b) An UpSet plot showing the overlap of small variants (<50bp).
