# Supplementary material for: Toward a standardized framework for pangenome graph evaluation: assessing crop plant pangenome variation graph construction from multiple assemblies
Source: Gigascience. 2025 Dec 4;14:giaf121. doi: 10.1093/gigascience/giaf121 (PMC12676463; doi:10.1093/gigascience/giaf121)
Supplement: giaf121_GIGA-D-25-00090_Original_Submission [file giaf121_giga-d-25-00090_original_submission.pdf]

GigaScience

Towards a Standardized Framework for Pangenome Graph Evaluation: Assessing Sorghum Pangenome Variation Graph Construction from Multiple Assemblies

--Manuscript Draft--

**Manuscript Number:**	GIGA-D-25-00090
**Full Title:**	Towards a Standardized Framework for Pangenome Graph Evaluation: Assessing Sorghum Pangenome Variation Graph Construction from Multiple Assemblies
**Article Type:**	Research
**Funding Information:**	Deutsche Forschungsgemeinschaft (GRK 2843) — Dr. Agnieszka Golicz<br>Office of the Permanent Secretary, Ministry of Higher Education, Science, Research and Innovation, Thailand (LOEWE Start Professorship) — Dr. Agnieszka Golicz
**Abstract:**	Pangenomes are crucial for understanding species-wide genetic diversity, delineating core and variable genes. This study compares three key pangenome graph assembly pipelines: Minigraph, PGGB, and Minigraph-Cactus, using publicly available Sorghum data. We introduce tailored metrics for comprehensive pangenome graph evaluation, including completeness, duplication levels and fidelity of structural variants.<br>By assessing the tools on Sorghum datasets, we gauge their efficacy in handling diverse genomic features. The analysis provides detailed insights into the strengths and limitations of Minigraph, PGGB, and Minigraph-Cactus, aiding researchers in informed tool selection. The metrics developed contribute to standardizing pangenome graph assessments, enabling robust and objective tool comparisons.<br>In conclusion, this benchmarking study advances our understanding of pangenome assembly tools and establishes a foundation for standardized evaluation metrics. We plan to further use these insights to optimize tool selection for specific applications, such as genome wide association studies, improving the accuracy of downstream analyses.
**Corresponding Author:**	Agnieszka Golicz<br>Justus Liebig University Giessen: Justus-Liebig-Universitat Giessen<br>Giessen, Hesse GERMANY
**Corresponding Author Secondary Information:**	
**Corresponding Author's Institution:**	Justus Liebig University Giessen: Justus-Liebig-Universitat Giessen
**Corresponding Author's Secondary Institution:**	
**First Author:**	Agnieszka Golicz
**First Author Secondary Information:**	
**Order of Authors:**	Agnieszka Golicz<br>Venkataramana Kopalli<br>Kübra Arslan<br>Noemia Morales-Díaz<br>Silvia Zanini
**Order of Authors Secondary Information:**	
**Additional Information:**	
**Question**	**Response**
Are you submitting this manuscript to a special series or article collection?	No

Powered by Editorial Manager® and ProduXion Manager® from Aries Systems Corporation

**Experimental design and statistics**<br><br>Full details of the experimental design and statistical methods used should be given in the Methods section, as detailed in our Minimum Standards Reporting Checklist. Information essential to interpreting the data presented should be made available in the figure legends.<br><br>Have you included all the information requested in your manuscript?	Yes
**Resources**<br><br>A description of all resources used, including antibodies, cell lines, animals and software tools, with enough information to allow them to be uniquely identified, should be included in the Methods section. Authors are strongly encouraged to cite Research Resource Identifiers (RRIDs) for antibodies, model organisms and tools, where possible.<br><br>Have you included the information requested as detailed in our Minimum Standards Reporting Checklist?	Yes
**Availability of data and materials**<br><br>All datasets and code on which the conclusions of the paper rely must be either included in your submission or deposited in publicly available repositories (where available and ethically appropriate), referencing such data using a unique identifier in the references and in the "Availability of Data and Materials" section of your manuscript.<br><br>Have you have met the above requirement as detailed in our Minimum Standards Reporting Checklist?	Yes

GigaScience has policies and guidelines in place for the use of generative AI-writing tools such as ChatGPT. If you have used such writing tools to assist with writing the manuscript this must be declared and cited in the text. Authors should not list AI-writing tools and other AI-assisted technologies as an author or co-author and should acknowledge that they are fully responsible for text generated or refined by AI-writing tools.<p><br><br>A summary of use (particularly in the introduction or among methods) needs to be included at the end of the paper, and the outputs should also be included as a supplementary file hosted in GigaDB or other open repositories. Please read our guidelines for more information. <p><br><br>By submitting to GigaScience, you are aware of the journal's AI-writing tools policy, and if you have declared use of such tools below, you have acknowledged this where appropriate in your manuscript and have made a summary of use and outputs available. <p><br><b>AI-assisted writing tools have been used in the preparation of this manuscript?	No

Powered by Editorial Manager® and ProduXion Manager® from Aries Systems Corporation

Click here to access/download;Manuscript;Ram.paper_04.03.2025.docx

Towards a Standardized Framework for Pangenome Graph Evaluation: Assessing Sorghum Pangenome Variation Graph Construction from Multiple Assemblies

Venkataramana Kopalli[1], Kübra Arslan[1], Noemia Morales-Díaz[2], Silvia F. Zanini[1], Agnieszka A. Golicz[1]

1. Department of Agrobioinformatics, IFZ Research Centre for Biosystems, Land Use and Nutrition, Justus Liebig University Gießen, Germany
2. Centre for Research in Agricultural Genomics, CRAG (CSIC-IRTA-UAB-UB) Campus UAB, Cerdanyola del Vallès, Barcelona, Spain

Abstract

Pangenomes are crucial for understanding species-wide genetic diversity, delineating core and variable genes. This study compares three key pangenome graph assembly pipelines: Minigraph, PGGB, and Minigraph-Cactus, using publicly available Sorghum data. We introduce tailored metrics for comprehensive pangenome graph evaluation, including completeness, duplication levels and fidelity of structural variants.

By assessing the tools on Sorghum datasets, we gauge their efficacy in handling diverse genomic features. The analysis provides detailed insights into the strengths and limitations of Minigraph, PGGB, and Minigraph-Cactus, aiding researchers in informed tool selection. The metrics developed contribute to standardizing pangenome graph assessments, enabling robust and objective tool comparisons.

In conclusion, this benchmarking study advances our understanding of pangenome assembly tools and establishes a foundation for standardized evaluation metrics. We plan to further use these insights to optimize tool selection for specific applications, such as genome wide association studies, improving the accuracy of downstream analyses.

Introduction

Pangenomics, a field focused on capturing the complete genetic diversity within a species, has gained significant attention in genomics, agriculture, and microbial studies [1]. Pangenomes offer a more comprehensive view of genetic variation within populations, species, or genera

by moving beyond the limitations of a single reference genome, such as its failure to represent the full genetic diversity, introducing reference bias, and missing structural variants [2]. Pangenomes are structured collections of genomic data that enable the study of genetic variant relationships while preserving the continuity of sequences and structural variations across individuals, allowing for the identification of complex DNA polymorphisms like structural variations (SVs), copy number variations (CNVs), and presence/absence variants (PAVs) [3,4].

Pangenomes have been applied in plant research to understand genetic mechanisms underlying trait variation, accelerate breeding processes, and improve crop performance [5]. Pangenome allows for the capture of genetic diversity essential for enhancing agronomical traits by incorporating wild species and multiple varieties and have been instrumental in studying transposable elements and accessory genomes, shedding light on their impact on genetic variation [6]. Pangenome analysis has been particularly valuable in crops like rice (*Oryza sativa*), maize (*Zea mays*), and rapeseed (*Brassica napus*), where it has revealed previously unknown genetic variations, aiding in accelerated genetic improvement [7-9].

Sorghum's significance for pangenome research lies in its genomic characteristics in addition to its applications in food, feed, or biofuel. Sorghum has a relatively small (~730 Mb) diploid genome (2n=20) which simplifies genome assembly. Repeat content constitutes approximately 61% of its genome, significantly influencing its structural variation (SV) [10]. Studies have identified extensive single nucleotide polymorphisms (SNPs), indels, and large-size presence/absence variants (PAVs) that contribute to sorghum's genetic diversity, an important factor for breeding [11]. Additionally, the pangenome approach in sorghum has revealed crucial structural variations, especially in genes related to stress responses, resistance to diseases, and adaptation to various environments. Known structural variants include numerous large-size PAVs and mobile elements, which further diversify sorghum's genome. This genetic variability is valuable for understanding the evolutionary dynamics and guiding crop improvement strategies [12,13].

There are several tools and pipelines that enable the creation of pangenome variation graphs, which are a graph-based representation of multiple genomes. These graphs model genetic diversity by encoding genomic sequences as nodes and their relationships, such as continuity or variation (e.g., SNPs, insertions, deletions, and structural variations), as edges. This allows for the efficient comparison, alignment, and analysis of genetic variation within a population. Minigraph, PGGB and Minigraph-Cactus are among these tools and pipelines used for these

purposes. Minigraph, PGGB, and Minigraph-Cactus were selected as they are among the most widely used and well-regarded tools for constructing pangenome graphs, offering robust and complementary capabilities. Minigraph is a versatile tool designed to construct and manipulate sequence graphs, is particularly effective in handling large structural variations (SVs) and is optimized for speed and scalability. It constructs pangenomes by aligning multiple genome assemblies against a reference, facilitating the detection of large insertions, deletions, and complex rearrangements [14] and was used to build the pangenome graph database representing presence–absence variation across sixteen bread wheat genomes [15]. The PanGenome Graph Builder (PGGB) is an innovative, reference-free pipeline designed to construct unbiased pangenome graphs. By utilizing all-vs-all whole-genome alignments and advanced graph embeddings, PGGB builds and continuously refines a model that allows for the identification of genetic variation, assessment of conservation, detection of recombination events and inference of phylogenetic relationships [16]. It was utilized to build a pangenome graph for a comprehensive genetic variation analysis of *Neisseria meningitidis* [17]. Minigraph-Cactus pipeline combines two methodologies: Minigraph and Cactus. Minigraph excels in building compact and accurate graphs from multiple genomes by aligning reads to a reference graph, while Cactus enhances this by integrating these graphs into a comprehensive pangenome structure. This combination facilitates both visualization and exploration of genetic variation, structural differences, and evolutionary relationships across diverse genomes, offering valuable insights into genomic diversity and complexity [18]. This pipeline was successfully utilized to build a pangenome graph and reveal extensive effector copy-number variation in spinach downy mildew [19].

SV detection and comparative genomics have traditionally relied on linear reference-based approaches to identify genomic differences between individual assemblies. To fully understand the benefits of a pangenome based approach, we compared Minigraph, PGGB and Minigraph-Cactus with two linear SV callers, SVIM-asm and SyRI. SVIM-asm is a linear variant caller designed specifically for long-read assemblies, enabling the accurate detection of complex structural variants, such as insertions, deletions, duplications, and translocations, by leveraging the detailed structural information encoded in such assemblies [20]. SyRI is a comprehensive tool for predicting genomic differences between related genomes using whole-genome assemblies (WGA). The assemblies are aligned using whole-genome alignment tools, and these alignments are then used as input to SyRI. SyRI identifies syntenic path (longest set of co-linear regions), structural rearrangements (inversions, translocations, and duplications), local

variations (SNPs, indels, CNVs, etc) within syntenic and structural rearrangements, and un-aligned regions [21]. In this manuscript we report extensive comparisons of the three leading pangenome variation graph construction pipelines using simulated and real-world data representing a sorghum diversity set. We assess the properties of the resulting pangenome graphs in terms of their completeness, encoded variation, but also suitability for downstream applications including read mapping. Our work expands the knowledge base and toolbox necessary to build high quality pangenome graphs in plants and especially crops, which often boast genomes affected by high levels of duplication, sequence diversity and transposable element activity [22,23].

Methods and Tools

This study employed a comprehensive workflow encompassing genome assembly, chromosome splitting, simulated genome generation, pangenome construction, variant calling, and downstream analyses. The workflow, as shown in Figure 1, provides an overview of the key steps, tools, and methods used across the study, linking each stage of the analysis pipeline.

Figure 1

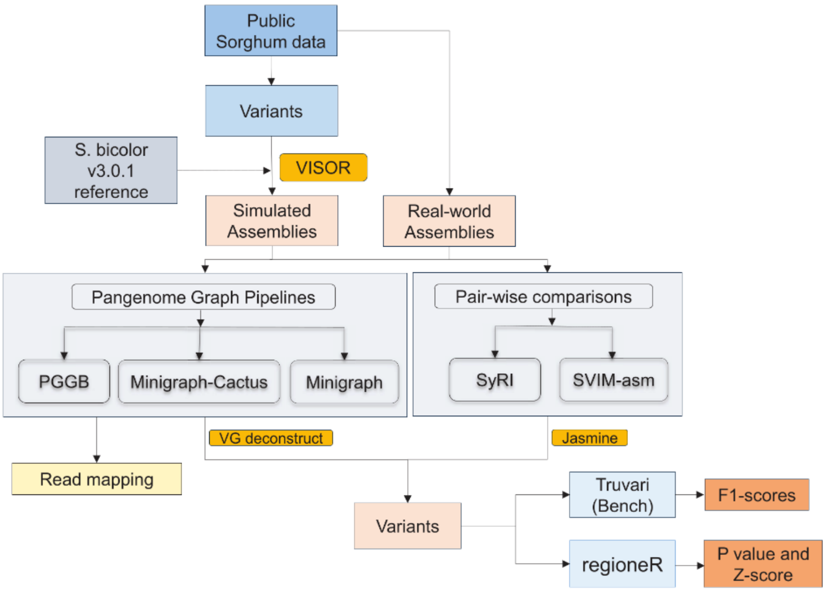

Figure 1: This flowchart illustrates the workflow used in our study to evaluate pangenome graph-building pipelines. Public sorghum data was used to generate simulated assemblies, which, along with real-world assemblies, were analyzed using pangenome graph-building pipelines and pairwise comparison tools. The resulting outputs were then assessed to evaluate their performance and accuracy.

Genome Assembly and Chromosome Splitting

Six assemblies were chosen based on the phylogenetic relationships published in a study by Tao et al [24]. We chose the accessions to be as diverse as possible to capture extensive

variation. We included two *S. bicolor* (IS8525 and Rio), one *S. bicolor verticilliforum* (AusTRCF317961), one *S. bicolor drummondii* (PI532566), one *S. bicolor margaritiferum* (IS19953), and an outlier *S. propinquum* (S369-1). Publicly available sorghum genome assemblies were downloaded [24]. The assemblies were then split into individual chromosome files using a shell script.

Simulated Genome Assemblies

Simulated genomes for all six accessions were generated using VISOR (Version 1.1.2.1) [25], with *Sorghum bicolor* v3.0.1 as the reference backbone, incorporating structural variants (SVs) identified in the six accessions. A filtering step was applied to retain only those SVs present in at least two of the six assemblies due to a large number of variants unique to one genotype prior to genome simulation. After filtering, 36,358 SVs remained, and all those exceeding 100 bp in size were used to simulate assemblies.

The headers in the FASTA files of the genome assemblies were modified to include the genome name as a suffix to the chromosome name. This adjustment simplified tracking individual assemblies and ensured efficient downstream analysis.

Pangenome Construction

Pangenomes were constructed per chromosome for the 10 chromosomes of *Sorghum bicolor* using PGGB, Minigraph-Cactus, and Minigraph for real-world and simulated datasets separately. The methods of construction were the same for both datasets.

PGGB (Version 0.5.4)

For each of the chromosomes, we created a FASTA file containing the corresponding seven sequences, six from the selected accessions and one from the reference. We compressed the resulting FASTA and created an index (.fai) file using samtools (Version 1.18) [26] faidx. FASTA and index files were used as input for graph construction, running PGGB with default parameters (k=19, p=90, s=5000). The order of the input samples was deemed insignificant as PGGB performs an all-vs-all alignment.

Minigraph-cactus (Version 2.7.2)

The Minigraph-Cactus tool was used with default parameters, using the *Sorghum bicolor* v3.0.1 as the reference along with the six genome assemblies. The command was run with –gfa, –viz, –draw, –og, and –gbz parameters. Minigraph-Cactus calculates the mash distance, which is a

metric used to estimate the genetic distance or similarity between two genome assemblies and reorders the input assemblies by placing the reference assembly first, followed by the remaining assemblies in decreasing order of mash distance to the reference.

Minigraph (Version 0.21)

The Minigraph pangenome was constructed for each chromosome of the assemblies with the *Sorghum bicolor* v3.0.1 reference as a backbone. Default parameters were used. Since Minigraph does not provide instructions for determining the order of input samples, a random order was used, assuming that the order is insignificant.

BUSCO (Version 5.2.2)

We assessed the completeness of the newly generated graphs with BUSCO, a bioinformatics tool that scores genome assemblies, gene sets, and transcriptomes by comparing them against a set of highly conserved orthologs. BUSCO evaluates the presence and quality of expected single-copy orthologs in the given dataset, providing insights into the completeness and potential gaps in the assembly [27].

We used the odgi flatten command from odgi tools (Version 0.8.6-0-ge647844f) [28] to convert the OG files of pangenomes of each chromosome into FASTA format and merged all the resulting FASTA files to reconstitute full assemblies. We ran BUSCO with the poales_odb10 lineage dataset and genome mode to assess the completeness of the pangenome.

Panacus (Version 0.2.3)

Panacus is a tool designed for quantifying the core size and estimating growth curves in pangenome graphs. It calculated several key statistics, including pangenome growth and core curves, by counting nodes, edges, and base pairs, and tracking how they were covered across paths. This tool enabled the grouping of paths, focusing on specific regions, and generated clear, interactive reports [29].

To analyze pangenome graphs, we first selected the paths corresponding to haplotypes by filtering and excluding specific references. We then used Panacus to calculate the coverage and pangenome growth for nodes with varying coverage and quorum thresholds (1/0, 2/0, 1/1, 1/0.5, and 1/0.1) using up to 4 threads. The resulting data was output as a TSV file. Finally, coverage histograms and pangenome growth curves were visualized, including estimated growth parameters (Supplementary Figure 1).

Variant Calling and filtering

PGGB and Minigraph-cactus

PGGB and Minigraph-Cactus variants were called with VG deconstruct (Version 1.54.0)[30]. Variants were called with respect to the path of the reference genome, along with parameters to process all snarls (-a) and to only consider traversals that corresponded to paths in the graph (-e). The input file was the GFA file output of PGGB, and this process was repeated for each chromosome-graph. The variants were filtered to include only variants with alternative alleles, and the multiallelic variants were split using bcftools norm (Version 1.16-23) [26] to facilitate downstream analysis.

Minigraph

We added path information (P-lines) to Minigraph's GFA by manually curating the output of minigraph -call, which retraced the assembly's path. We then called the variants with VG deconstruct.

SVIM-asm (Version 1.0.3)

SVIM-asm is a structural variant caller for haploid or diploid genome-genome alignments. We ran SVIM-asm separately for each assembly against the *Sorghum bicolor* v3.0.1 reference as it calls SVs from pairwise assemblies aligned with Minimap2 (Version 2.26-r1175) [31]. SAM outputs were converted to BAM using samtools, then BAM files were sorted and indexed to be used as inputs to SVIM-asm in haploid mode to call the variants.

SyRI (Version 1.6.3)

We used SyRI to compare chromosome-level assemblies and identify structural rearrangements and synteny. The same pairwise alignments generated with Minimap2 for SVIM-asm were used as input for SyRI variant calling. The initial VCF file produced by SyRI was missing genotype information and flagged 'N's as Highly Diverged Regions (HDRs). We resolved this by filtering out the 'N's, manually adding FORMAT and SAMPLE columns, and updating the VCF header for compatibility with bcftools. After merging the individual VCF files for each method with Jasmine (v1.1.5) [32], we refined the results by sorting using bcftools sort and splitting multiallelic variants using bcftools norm.

Truvari Bench (Version 4.1.0)

To assess the accuracy of both variant callers, we calculated and compared the precision, recall, and F1 scores of called variants from the pangenomes against the true set of variants from Tao et al., 2021 which was used to simulate the genome assemblies. Truvari bench was run with default parameters [24,33].

<u>regioneR</u> (Version 1.36.0)

We used permutation testing with the regioneR package to evaluate the performance of PGGB and Minigraph-Cactus variant calling with real-world assemblies [34]. It enabled for the assessment of overlaps between genomic regions by comparing observed data against a distribution generated from randomized genomic regions to determine statistical significance. We filtered out pangenome variants longer than the longest variant in the true set, as their inclusion disproportionately increased the expected overlap during the regioneR permutation analysis. Longer variants had a higher likelihood of overlapping by chance due to their size, which affected the randomization process and inflated the baseline expected overlap. The permutation test was conducted using the permTest function, with 100 permutations of genomic regions. The randomizeRegions function was employed to generate these randomized regions, and the numOverlaps function was used to evaluate the overlap between the variant sets and the true variants. We set count.once=TRUE to ensure that each true-set variant was counted only once, avoiding overestimation from multiple overlaps with the same pangenome variant.

<u>Surpyvor</u> (Version 0.5)

Surpyvor is a Python package designed for analyzing structural variants (SVs) enabling for the visualization and quantification of SV relationships within genomic data [35]. We utilized the venn function for creating Venn diagrams and the upset function for generating UpSet plots. For large variant comparisons, we used the default parameters; however, for small variant comparisons, we used the -snv option to override the default SURVIVOR method and opted for bcftools instead, as the SURVIVOR method was primarily tailored for large SV analyses.

<u>Repeat content Analysis</u>

To assess repeat content in the pangenome variants, we first generated a transposable element (TE) library using the EDTA (Extensive de novo TE Annotator) pipeline (Version 2.1.1) [36] EDTA was applied to generate a non-redundant high-quality TE library. This library was then formatted as a BLAST database with makeblastdb to allow for efficient sequence comparison. Variant sequences from the VCF file were extracted and formatted in FASTA, with each

sequence labelled by its genomic coordinates for unique identification. Sequence headers were standardized to remove any non-compatible characters to facilitate downstream analysis. We then used BLASTN (Version 2.12.0) [37] to align these variant sequences against the TE database, retrieving only the top hit for each variant. The results were sorted by query coverage, and only the best alignment for each variant was retained. We further filtered the results to include only those matches with a query coverage of 80% or higher to ensure high confidence in the repeat classification. This approach enabled us to reliably identify and characterize repeat-associated variants within the pangenome.

Missing genes and assembly coverage statistics

To detect missing genes across assemblies in the pangenome graph, we first generated FASTA sequences for each assembly's path in the graph using odgi paths. Gene annotations from the reference genome were mapped to each pangenome assembly FASTA with Liftoff (Version 1.6.3) [38], identifying unmapped genes as potentially missing. Each assembly was also aligned to its path in the pangenome graph using Minimap2. The alignments were processed with samtools to generate coverage statistics.

Read Simulation and Mapping

We evaluated the performance of linear and graph mapping by using simulated reads, which were generated using the code developed by Rice et al., 2023 [39]. This process involved three main steps: first, simulating reads from the graph; second, aligning those reads to both the graph using VG Giraffe (Version 1.58.0) [40] and to the *Sorghum bicolor* v3.0.1 linear reference using Minimap2, and finally, comparing the results from both mapping methods based on the alignment quality and correctness rates.

Individual OG files generated for each chromosome were merged with odgi squeeze. The merged OG file was converted back to GFA format with odgi view.

Read Simulation: We simulated one million reads from the corresponding pangenome graphs (which were then used as reference for read mapping) using VG sim [41]. The length of each simulated read was set to 150 bps, the error rate was set at 0.24%, the indel rate was set to 0.029%, the mean insert size was set to 570 bps, and the standard deviation of the insert size was set to 165. The resultant GAM file was converted to FASTQ format with VG view.

Mapping to VG Giraffe: The simulated reads were mapped back to the graph using VG Giraffe. To enable VG Giraffe to map reads to the graph, indexes were generated using VG autoindex

with the workflow parameter set to Giraffe, which produced the necessary files for mapping (.gbz, .min, and .dist). We annotated the GAM file and compared it to the simulated reads using VG gamcompare, generating cumulative alignment and correctness rates.

Mapping to Linear reference: We extracted reference paths from the graph using VG paths, mapped the simulated reads with Minimap2, and injected the alignment back into the graph. We annotated the GAM file and compared it to the simulated reads using VG gamcompare, generating cumulative alignment and correctness rates.

Real-world reads: We downloaded paired-end reads datasets of sorghum [24] to test mapping efficiency in a real-world scenario. Reads were mapped to the graph using VG Giraffe and to the linear reference using Minimap2, with the same parameters as the simulated reads.

Results

Simulated Assemblies

Pangenome size and content

The size of the pangenomes was similar between pipelines, while the number of nodes greatly varied across methodologies. The PGGB pangenome consisted of 851 Mb with ~190,000 nodes, the Minigraph-Cactus pangenome was 795 Mb with ~220.000 nodes, and the Minigraph pangenome was 878 Mb with only 950 nodes (Figure 2). The linear reference genome had a size of 688 Mb, demonstrating that all pangenomes exhibited an increase in total genomic content compared to the linear reference. This highlighted the additional complexity and variation encompassed by the pangenome graph. Minigraph stood out in our simulation, as it failed to detect variants which resulted in a smaller number of very long nodes. The average node length for Minigraph was 968,806 bp, which was very high compared to PGGB's 4,517 bp and Minigraph-Cactus' 3,714 bp. The mean BUSCO completeness for the six input simulated assemblies was 98.4% with 1.6% duplication (Supplementary Table 1a), comparable to BUSCO completeness of 98.4% with 1.7% duplication seen in the three pangenomes (Supplementary Table 2a).

Figure 2

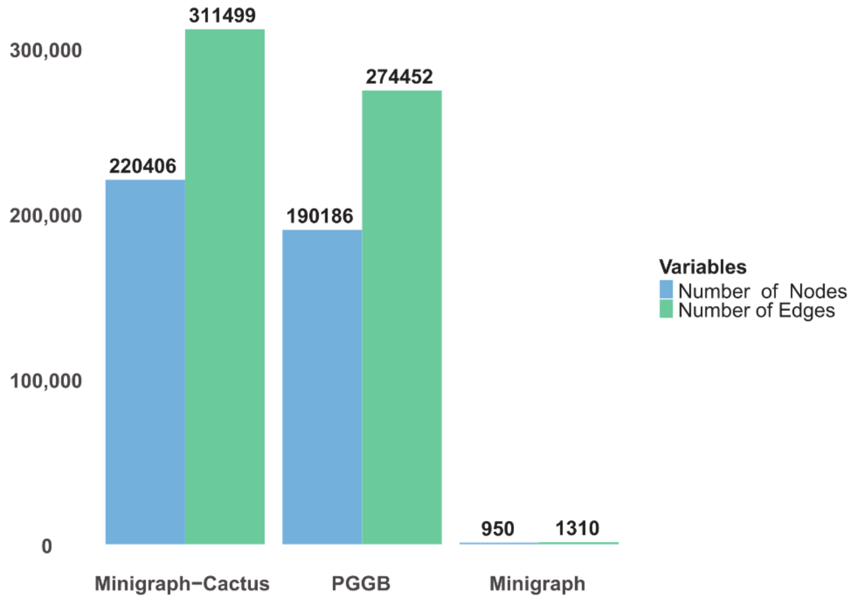

Figure 2: Comparison of the number of nodes and edges for Minigraph-Cactus, PGGB, and Minigraph pangenome graphs constructed from simulated assemblies.

Variant calling

To assess the accuracy of variant calling we calculated F1 scores for variants identified from pangenomes constructed with simulated assemblies of Minigraph, PGGB, and Minigraph-Cactus. This comparison was made against a set of true variants from a published dataset using the Truvari benchmarking tool. The average F1 score for PGGB was 0.83, while Minigraph-Cactus achieved a higher average F1 score of 0.89 (Figure 2). Minigraph detected a considerably low number of variants in a simulated scenario, an unexpected behaviour also reported by other users [42,43]. The results were consistent when cross-checking the Minigraph output with the Minigraph output from the Minigraph-Cactus pipeline. The limited number of variants detected by Minigraph prevented the calculation of a meaningful F1 score and the output did not provide sufficient data for reliable evaluation.

Figure 3

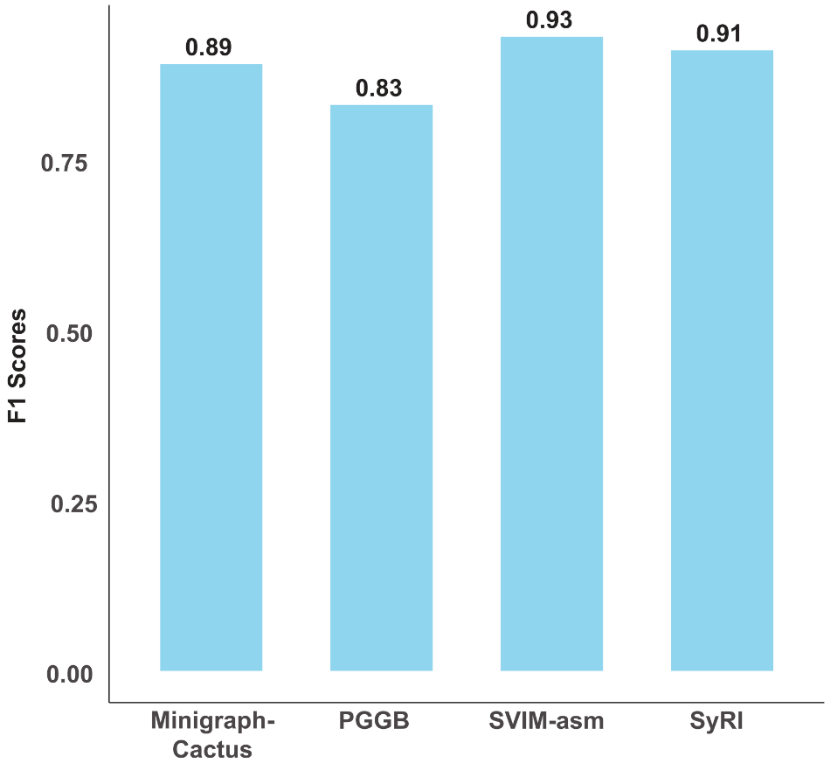

Figure 3: Comparison of F1 scores for non-redundant merged variant set of variants detected from all assemblies. SVIM-asm achieves the best performance with an F1 score of 0.93, followed closely by SyRI with 0.91. Minigraph-Cactus also performs well with a score of 0.89, while PGGB has the lowest score at 0.83.

The variants were also called using linear-based SVIM-asm and Syri (Figure 3) to compare the variant calling efficiency of graph-based PGGB, Minigraph-Cactus and Minigraph. The F1 score comparisons across all assemblies suggested that pairwise comparison methods outperformed pangenome graph pipelines in the SV calling task. SVIM-asm achieved the highest overall F1 score of 0.93, followed by SyRI at 0.91, both outperforming Minigraph-Cactus (0.89) and PGGB (0.83) (Figure 3). SVIM-asm and Syri also exhibited higher consistency and F1 scores across multiple genome assemblies (Figure 4).

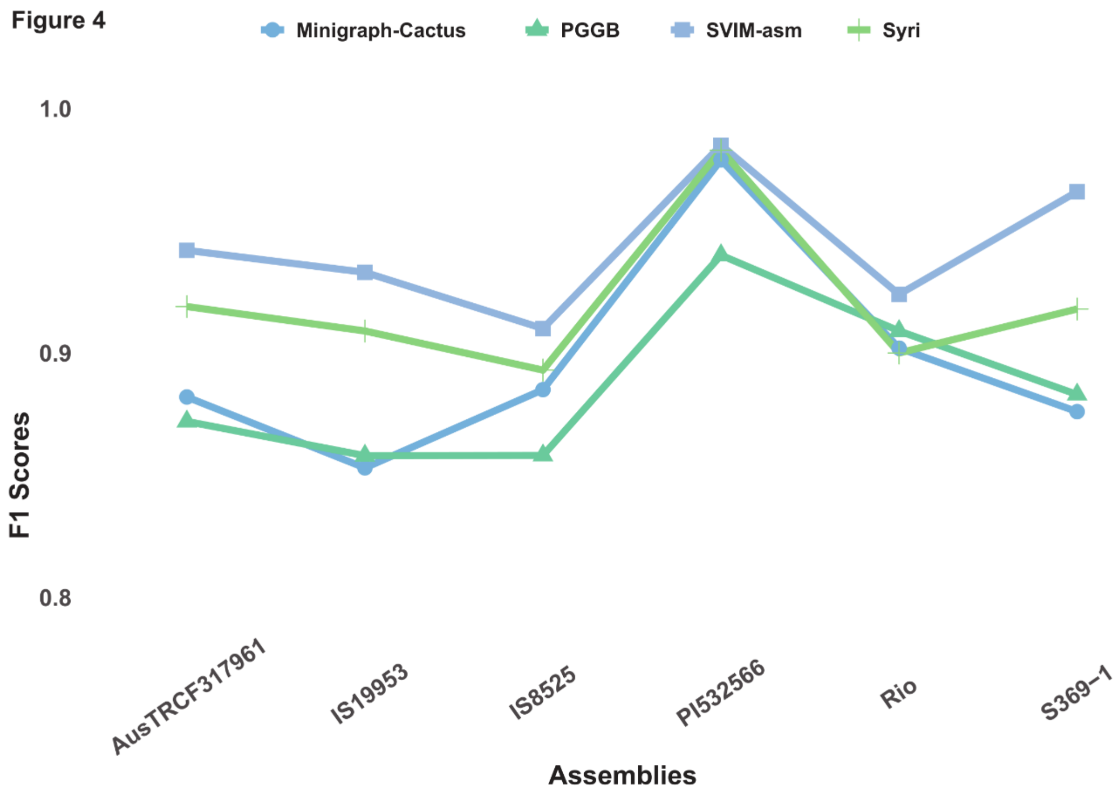

Figure 4: F1-score comparison for individual samples. The comparison of F1 scores highlights differences in F1 score achieved for different samples. All pipelines exhibit similar performance trends.

Pairwise variant callers offer slightly better variant detection accuracy and robustness across different genome assemblies compared to pangenome construction pipelines, as shown in Figure 5 in a simulated scenario. Nevertheless, the vast majority of SVs were detected across all methodologies, demonstrating the suitability of graph-based approaches while revealing differences in sensitivity and specificity across tools.

Figure 5

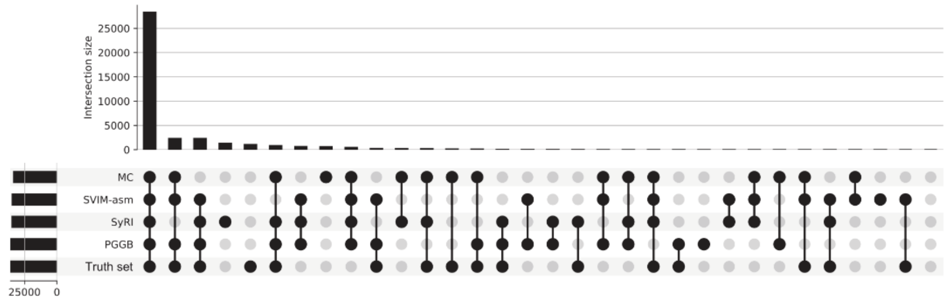

Figure 5: UpSet plot displaying the intersection of structural variants (SVs) called by SyRI, SVIM, PGGB, and Minigraph-Cactus with the true set for simulated datasets. Each row corresponds to the set of SVs identified by a specific tool, represented by SyRI, SVIM-asm, PGGB, and Minigraph-Cactus. The horizontal bars on the left indicate the total number of SVs detected by each tool. Connected dots across rows represent intersections between the tools, indicating shared SV calls. The vertical bars above the plot show the size of each intersection, with higher bars corresponding to larger overlaps in SVs between the respective tools. The largest intersection, displayed on the far left, represents the SVs shared by all the tools.

Real-World assemblies

Pangenome size and content

In addition to simulations, we also constructed pangenome graphs using six assemblies representing real-world data. Pangenome graph sizes varied between the three pipelines, with the PGGB pangenome reaching 4.7 GB, the Minigraph-Cactus pangenomes at 4.2 GB, and the Minigraph pangenomes being considerably smaller at 920 MB. For comparison, the size of the linear reference genome was 688 Mb, indicating that all pangenomes represented an increase in total genomic content relative to the linear reference, reflecting the added complexity and variation captured by the pangenome graph. The number of nodes and edges in the graph corresponds to its size, as shown in Figure 6. Size differences between Minigraph and the other two graphs is due to the inclusion of only variants > 50bp, while PGGB and Minigraph-Cactus do not have a size filter. When compared to the graph sizes of simulated assemblies, the size and content of real-world pangenomes increase for PGGB and Minigraph-Cactus, consistent with the presence of many additional short variants (SNPs and InDels) which were not part of simulations.

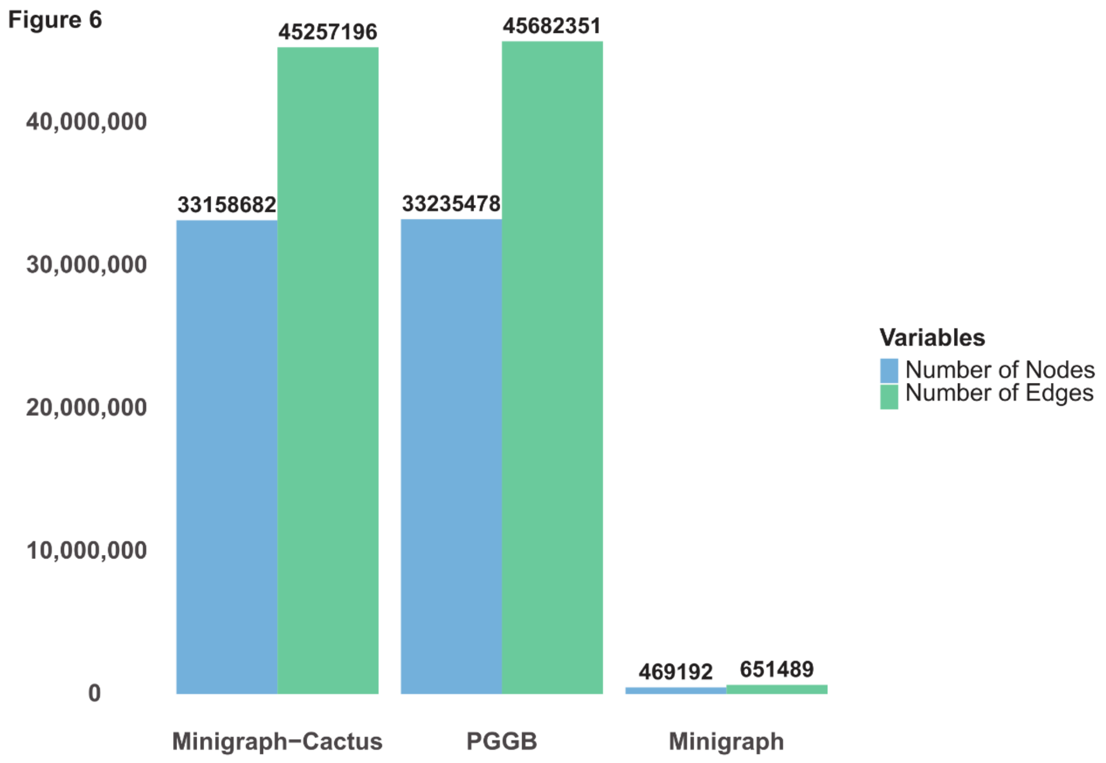

Figure 6: Comparison of the number of nodes and edges for Minigraph-Cactus, PGGB, and Minigraph pangenome graphs built with real-world assemblies.

The input real-world assemblies had a mean BUSCO completeness of 95.4% with 2% duplication (Supplementary Table 1b). Pangenomes built with Minigraph-Cactus, PGGB and Minigraph had BUSCO completeness of 81.9% with 9.9% duplication, 82.9% with 9.4% duplication and 78.2% with 2.5% duplication, respectively (Supplementary Table 2b). The reduction in completeness scores is expected, as in some cases genes will be broken up across multiple nodes. The duplication levels <10% points to a successful genomic data compression, which is one of the key tasks of pangenome graph construction.

We tracked the growth and coverage statistics of graphs using Panacus. Growth helps track the accumulation of genes and genomic features, while coverage statistics assess the phylogenetic diversity represented. Overall, all three pangenome pipelines show similar graph expansion dynamics, with less nodes added with each new sample.

Figure 7

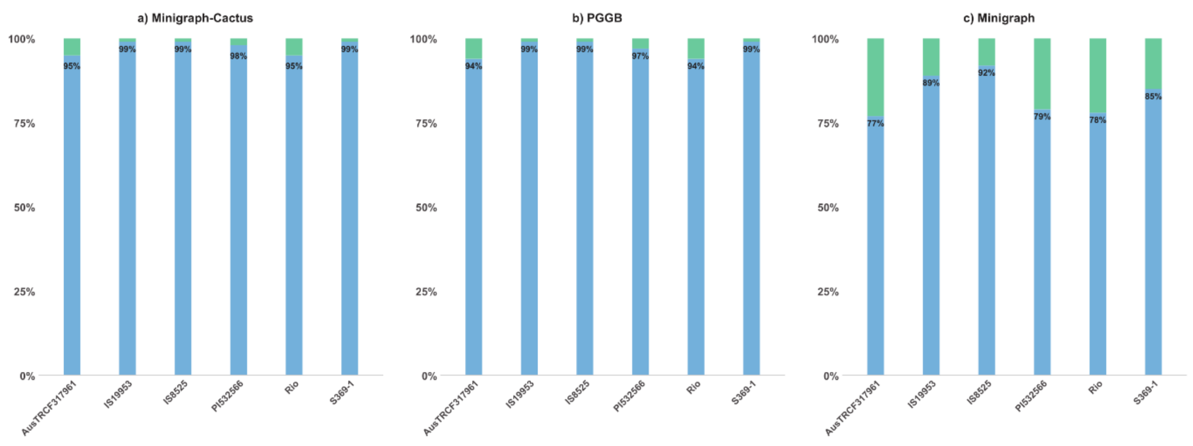

Figure 7: Comparison of the coverage percentages of each input assembly within the pangenome graph constructed using: (a) Minigraph-Cactus (b) PGGB (c) Minigraph. The percentage of each assembly included in the pangenome graph is represented by coloured bars, with light blue indicating the included portion and light green showing the excluded portion for each assembly.

Pangenome construction can lead to data loss, when sequences present in the input assemblies are not included in the pangenome graph. We evaluated the representation of each input assembly within the pangenome graph across all three pangenome pipelines. We observed that all assemblies were represented with a coverage above 94% in both the Minigraph-Cactus (Figure 7a) and PGGB (Figure 7b) pangenomes. When assessing gene presence in regions present in the assembly but missing from the graph for each assembly, we found that one gene from assembly AusTRCF317961, four genes each in IS19953 and IS8525, 14 genes in PI532566, three genes in Rio and 25 genes in S369-1 were not found in the assemblies recovered from the graph (Figure 8). These numbers were similar in both Minigraph-Cactus and PGGB pangenomes, but Minigraph showed comparatively lower proportion (averaging 83%) of assemblies included in the graph (Figure 7c) and consequently a higher number of missing sequence and genes (Figure 8).

Figure 8

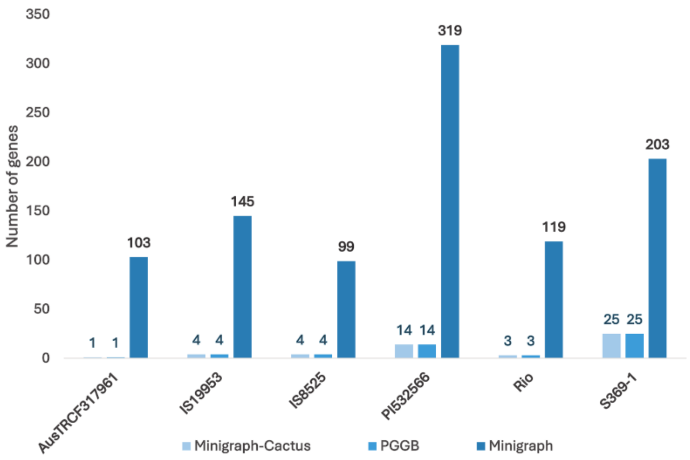

Figure 8: Comparison of missing gene counts across different assemblies in a pangenome graph built with Cactus, PGGB, and Minigraph. Minigraph has the most missing genes in all assemblies.

Variant Calling

We performed a permutation test for variants called from pangenomes (Minigraph-Cactus, PGGB and Minigraph) constructed with real-world assemblies and also and pairwise variant callers (SVIM-asm and SyRI) to compare them with previously reported SVs in sorghum[24]. This test, conducted using the regioneR package, involved generating 100 randomizations of genomic regions to assess the significance of the observed overlaps between pangenome variant sets and the previously reported variant set. All three pipelines, Minigraph-Cactus, PGGB and Minigraph, demonstrated statistically significant results with a permutation count of 100 iterations and randomization using randomizeRegions, yielding a p-value of 0.0099 and high Z-score (Supplementary Figure 2a ,b, c). These findings indicate that for real-world data, the SVs found using a pangenomic approach overlap previously reported variants much more than would be expected by chance alone. Pairwise variant callers, SVIM-asm and SyRI, also show significant overlap, though to a lesser degree than the pangenome pipelines (Supplementary Figure 2d, e). Together, with the results from simulations, this suggests that building a pangenome graph using Minigraph-Cactus and PGGB is a viable approach for variant calling.

We analysed the proportion of variant types identified by each tool and observed that Minigraph-Cactus, PGGB and SyRI identify variants of all types and have similar proportions of variant distribution. They also include an 'others' category for complex or less common

variant types, including duplications, inversions, and other structural rearrangements (Figure 9).

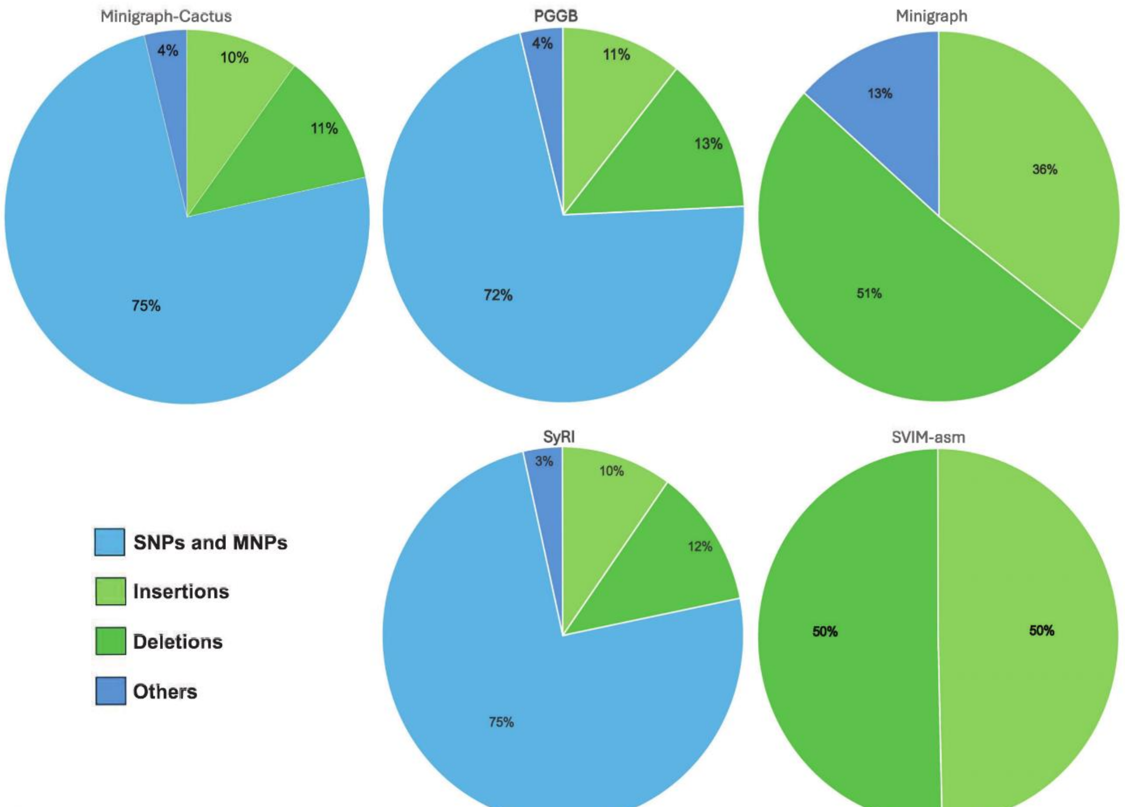

Figure 9

Figure 9: Pie charts comparing the proportion of different variant types called by Minigraph-Cactus, PGGB, Minigraph, SVIM-asm, and SyRI. Minigraph and SVIM-asm focus on structural variants and do not call SNPs or MNPs. The “Others" category includes complex variant types like duplications, inversions, and other structural rearrangements.

While these results confirm the quality of variants identified by pangenome methods, we also evaluated the overlap of variants between pangenome pipelines and pairwise variant callers (Figure 10a). Notably, PGGB and Minigraph-Cactus exhibit higher overlap with each other compared to pairwise callers SVIM-asm and Syri. We assessed the performance of all tools in detecting small variants in real-world assemblies, focusing on Indels under 50bp and SNPs (Figure 10b). Minigraph-Cactus detected approximately 7.7 million SNPs and 2.3 million small Indels, while PGGB identified around 7.6 million SNPs and 2.6 million small Indels. Pairwise variant caller SyRI detected 3.2 million SNPs and 0.93 million small Indels while SVIM-asm detected no SNPs and 11k small Indels which is relatively small, resulting in a reasonable overlap between PGGB, Minigraph-Cactus and SyRI but negligible overlap of variants across all four methods (Figure 10b). Minigraph-Cactus and PGGB identified 0.59 million and 0.56

million multiallelic sites, respectively, while Minigraph only found 46k multiallelic sites (Figure 10c).

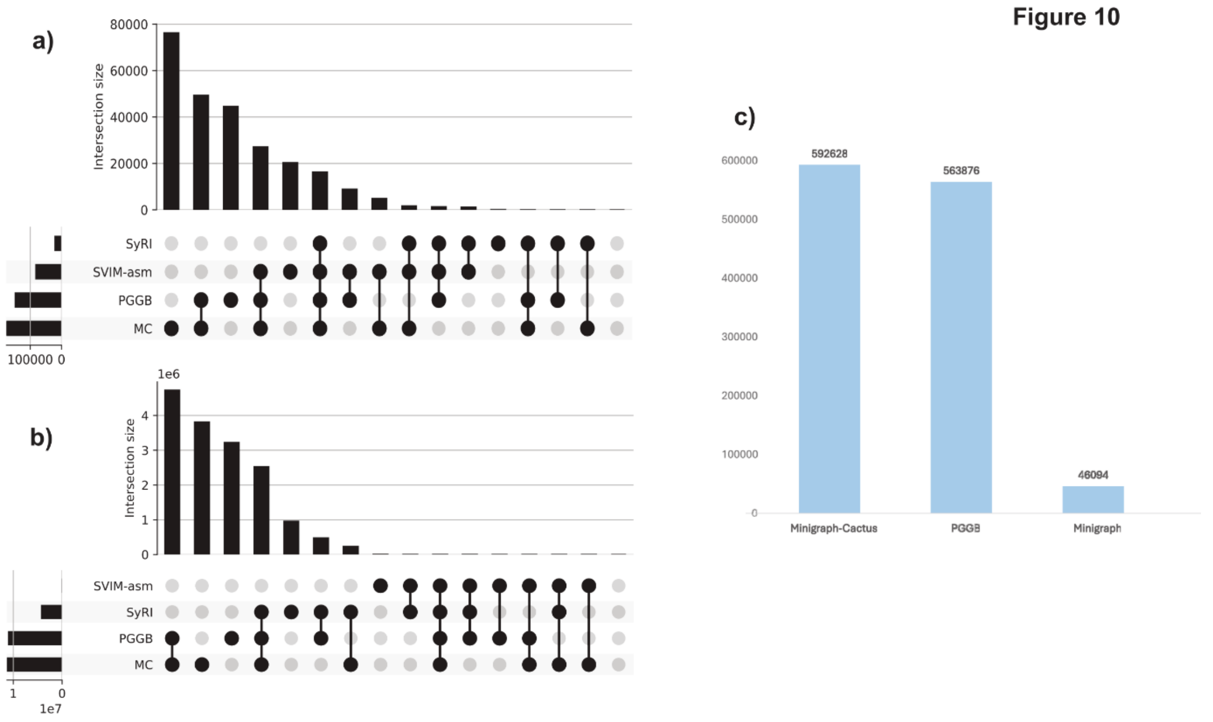

Figure 10: Overlap of variants in real-world assemblies. a) An UpSet plot showing the overlap of large variants (>50bp) between different methods, the bars represent the number of shared variants between dataset combinations, with the matrix below indicating which datasets are included. b) An UpSet plot showing the overlap of small variants (<50bp). c) Number of Multiallelic sites detected by each of the three pipelines.

We also analyzed the similarity of variants called by PGGB and Minigraph-Cactus to repeats by comparing them to the *S. bicolor* repeat library. We found that 28% of variants in both PGGB and Minigraph-Cactus matched repeats. When only matches with over 80% coverage were considered, these results were reduced to 7.5% for PGGB variants and 11% match for Minigraph-Cactus variants (Figure 11a). Both Minigraph-Cactus and PGGB call a high number of variants unique to each of the pipelines (Figure 11b). We also analysed repeat similarity of those unique variants and found that 48% of PGGB unique variants and 38% of Minigraph-Cactus unique variants matched with repeat locations (Figure 11b).

Figure 11

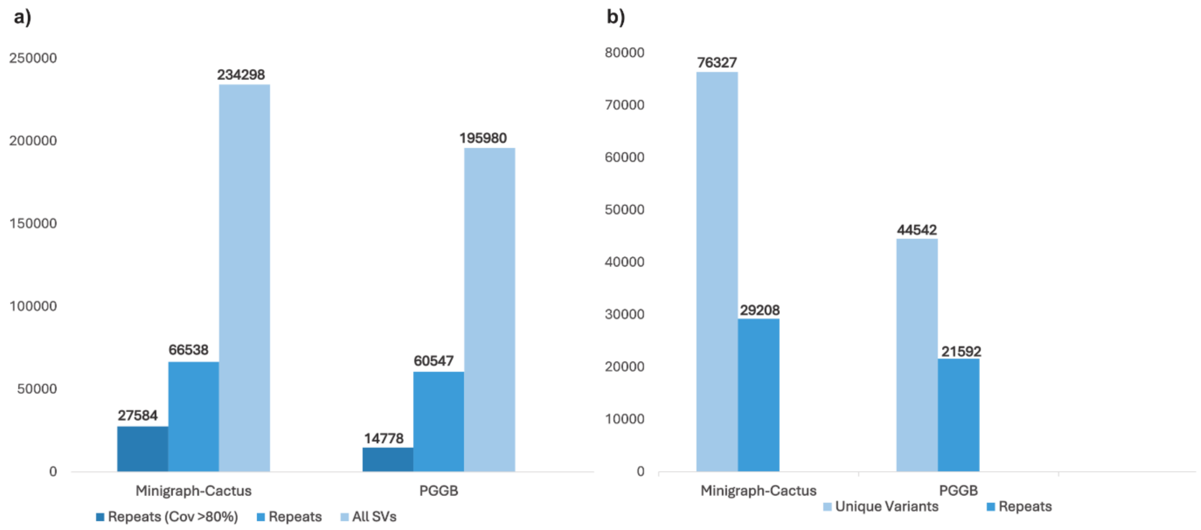

Figure 11: Comparison of SVs with similarity to repeats for Minigraph-Cactus and PGGB; a) Total counts of structural variations (All SVs) and SVs with similarity to repeats detected by each method, including the count of SVs with similarity to repeats after applying 80% coverage filter b) Count of unique variants identified by Minigraph-Cactus and PGGB and the count of SV with similarity to repeats in those unique variants.

Read Mapping

One of the key objectives of a pangenome graph is to act as a reference for read mapping. We therefore assessed mapping outcomes for graphs produced with different pipelines. We were unable to generate mapping indexes for the PGGB pangenome graph due to heavy computational and memory requirements and hence were unable to test it for read mapping. This issue has been previously reported during chicken pangenome analysis [39]. Following the approach of Rice et.al., 2023, we evaluated the performance of linear and graph-based mapping by simulating reads from the entire pangenome graph, which includes all seven assemblies (six input assemblies and the *Sorghum bicolor* v3.0.1 reference), rather than just from a single reference genome. The simulated reads were then mapped to both the graph using VG Giraffe and the *Sorghum bicolor* v3.0.1 linear reference using Minimap2. This allowed us to assess the mapping performance across the genetic diversity captured in the pangenome. For Minigraph-Cactus, the Giraffe results show better mapping compared to the corresponding Minimap2 alignments (Figure 12). Specifically, at a high minimum mapping quality (min_mapQ) threshold of 60, although Giraffe (Minigraph-Cactus) only maps 38.27% it achieves a correct read mapping rate of 99.68%, substantially better than Minimap2's, which maps 48.67% reads with only 58.18% reads mapped correctly (Figure 12a).

Figure 12

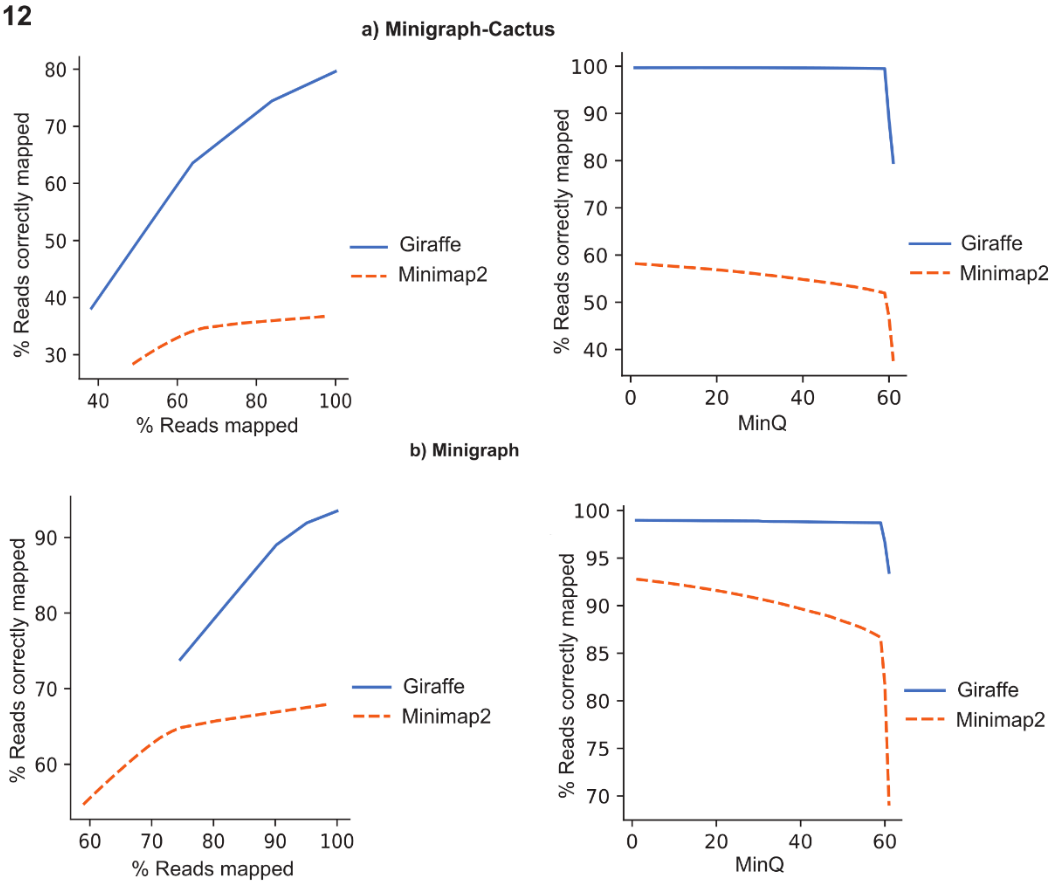

Figure 12: Comparison of alignment outcomes for reads simulated from Minigraph-Cactus and Minigraph graphs to corresponding graph and linear references. This figure compares the performance of VG Giraffe and Minimap2, on (a) Minigraph-Cactus (b) Minigraph. In the left panels, the x-axis shows the percentage of reads mapped by each aligner, with the y-axis representing the percentage of correctly mapped reads. Giraffe consistently outperforms Minimap2. The percent mapped starts from the percentage of reads mapped with MinQ 60 and progresses to MinQ 0. For instance, in (a) Minigraph-Cactus, Giraffe maps 38.27% of reads with MinQ 60, of which 38.15% are correct. In (b) Minigraph, Giraffe maps 74%, with 73.85% correct. In the right panels, the x-axis shows the minimum quality score (minQ), with the y-axis indicating the percentage of correct mappings. Giraffe maintains near-perfect excellent performance until a sharp drop at the highest thresholds, while Minimap's performance declines steadily.

Additionally, Giraffe (Minigraph-Cactus) maintains a high rate (over 98%) of correctly mapped reads, whereas Minimap2's has lower proportion of correctly mapped read at similar thresholds (Figure 12a). At mapQ 1, the percentage of total reads aligned correctly is 79.60%, when compared to Minimap2's 35.49% (Figure 12a). This demonstrates that Giraffe using the Minigraph-Cactus pangenome offers substantial improvements in both mapping accuracy and total alignment correctness compared to linear-based approaches. It is notable that despite simulating the reads from the same pangenome graph, some reads did not align to the graph at mapQ 1, which likely reflects alignment ambiguity in highly repetitive regions as the mapQ 1 threshold may exclude low-confidence alignments, contributing to the reduced alignment rate.

Giraffe performs well also with the Minigraph pangenome. At a minQ threshold of 60, Giraffe (Minigraph) maps 74.62% of reads with 95.08% correct read mapping rate, which is better than the corresponding Minimap2's mapping of 58.93% reads with 92.78% correct read mapping rate. The correctness rate for Giraffe (Minigraph) remains high at around 98%, similar to the results with Minigraph-Cactus. Furthermore, the percentage of total reads aligned correctly with Giraffe (Minigraph) reached 91.91% at minQ 1, when compared to Minimap2's 65.74% (Figure 12b), highlighting its superior mapping ability compared to both Minigraph-Cactus and Minimap2. The comparatively good read mapping performance of Minigraph-only graph can be attributed to much lower graph complexity due to the absence of small variants, which makes read simulation and mapping task much more straightforward. Minigraph-Cactus builds more complex graphs, capturing a wider range of genetic variations. While this detailed approach provides a richer genomic view, it also makes the read mapping process slower and more complex.

Figure 13

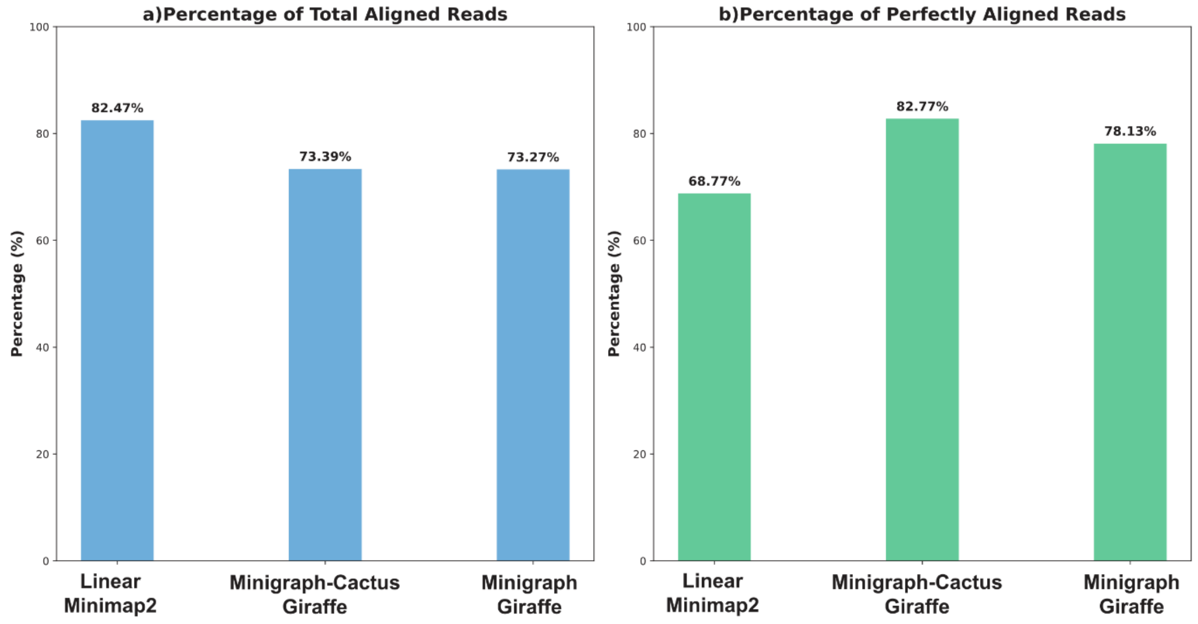

Figure 13: Comparison of read alignment metrics for Minigraph-Cactus, Minigraph and Minimap2 for real-world reads. (a) displays the percentage of total reads successfully aligned by each tool: Minimap2 achieved the highest alignment rate at 82.47%, followed by Minigraph-Cactus with 73.44% and Minigraph with 73.27%. (b) shows the percentage of perfectly aligned reads, where Minigraph-Cactus outperformed the others with an alignment rate of 82.77%, Minigraph had 78.13% and Minimap2 only achieved 68.77%, highlighting the lower quality of read alignments to the linear reference (Minimap2) in (a) when compared to pangenome graph references (Minigraph-Cactus and Minigraph).

We tested the read mapping ability of both pangenomes and a linear reference in the case of real-world reads as well and the results showed the excellent ability of pangenomes to map

real-world reads [24]. Minigraph-Cactus aligns 73.44% of total reads (Figure 13a) out of which, 82.77% of reads are aligned perfectly (Figure 13b). On the other hand, Minigraph aligns 73.27% of total reads (Figure 13a) out of which 78.13% are aligned perfectly (Figure 13b). While Minimap2 aligns 82.47% of total reads (Figure 13a) out of which only 68.77% are aligned perfectly (Figure 13b).

When comparing mapping ability of pangenomes to linear references, it is evident that pangenomes consistently offers superior alignment performance. Giraffe's alignment to both the Minigraph-Cactus and Minigraph pangenomes resulted in higher percentages of correctly mapped reads and total correct alignments compared to Minimap2's alignment to linear references.

Discussion

Previous benchmarking efforts focused mainly on animal genomes [30,39,44] which, compared to plants, can have very different properties including much higher levels of intra- and inter- species collinearity [45]. Assessing the methods' performance with crop data is therefore of high importance. This study offers a comparison of three key pangenome assembly pipelines (Minigraph-Cactus, PGGB and Minigraph) using sorghum as a model, highlighting how each approach handles the complexity of pangenome construction, content, and performance in variant calling and mapping. One of the key tasks of pangenome graph construction pipelines is an effective identification of regions of sequence correspondence and variation from whole genome alignments. We compared performance of the state-of-the-art pangenome pipelines (Minigraph-Cactus, PGGB, Minigraph) with established pipelines for SV identification from pairwise genome alignments (SVIM-asm and SyRI). To thoroughly evaluate the performance of the SV calling pipelines, we used both simulated and real-world datasets. The simulated data, based on a reference backbone and containing known SVs, provided a controlled environment where we could assess variant calling accuracy with a known true set of SVs. In contrast, the real-world data, which includes inherent biological complexities such as sequencing errors and structural variation, allowed us to test the pipelines under more challenging and realistic conditions. This dual approach ensured a comprehensive evaluation of each pipeline's performance across both ideal and complex scenarios.

While the pairwise variant callers (SVIM-asm and SyRI) had slightly higher F1 scores in the simulations compared to the top performing pangenome graph construction pipeline (Minigraph-Cactus), overall, pangenome graph-based methods showed strong performance in

simulations and good alignment with previously reported variation in real world datasets. We speculate that the performance of pairwise variant callers could be inflated in simulations due to over-simplified scenarios, without small or multi-allelelic variants. While the complexity of Minigraph-Cactus and PGGB generated graphs leads to substantial disparity in the number of variants detected by the two pipelines, they both capture a large number of variants which appear to have been missed by the pairwise variant callers in real-world scenarios. Pangenome graph approaches appear effective in identifying variants across multiple assemblies and capture both common and rare variants. Pangenome graph construction pipelines therefore emerge as a robust solution when dealing with complex, large datasets in a real-world scenario.

The study revealed that pangenome-based approaches generally offer better alignment accuracy compared to traditional linear references for mapping performance. Giraffe, used with both Minigraph-Cactus and Minigraph pangenomes, consistently outperformed Minimap2 in correctly mapping reads, underscoring the advantages of graph-based methods for managing complex genomic data. However, PGGB's computational demands were a significant challenge, especially in generating mapping indexes, indicating the need for further optimization.

The identification of genetic variation through pangenome methods becomes particularly challenging when dealing with highly complex variations, such as nested or tandemly repeated transposable element insertions [46] Recent analysis in rice demonstrates that the Minigraph-Cactus pipeline can accurately identify five different alleles of a complex locus consisting of nested and tandemly repeated transposon insertions within a pangenome of 20 different accessions of Oryza sativa. Compared to a linear pangenome analysis, pangenome graph approach allowed for improved characterization of multiple related alleles. However, correct genotyping of multiple alleles in a large population using short reads remained challenging.

In this study, we constructed pangenomes for each chromosome separately, as this approach offers several advantages, including reduced processing time, simplified data management, and easier identification of variations within individual chromosomes. However, it does not account for the connections between chromosomes. To investigate complex chromosomal changes, such as Robertsonian translocations or centromeric homology [44], building a whole genome pangenome may be essential.

Our findings emphasize the importance of selecting tools aligned with specific research objectives, whether prioritizing variant discovery, optimizing computational resources, or

ensuring high mapping accuracy. PGGB is designed as a completely reference free approach relaying on all-vs-all alignments, however this appears to result in a potential trade-off in precision and recall of SV identification. Minigraph-Cactus progressively builds a graph and appears to offer a balanced integration of new sequences with robust performance. Minigraph, though efficient for identifying large structural variants, will miss finer variant details, particularly in highly diverse regions. Despite the advantages of pangenome-based approaches in capturing both common and rare variants across multiple assemblies, this study also highlights that these methods still require improvements. Additionally, refining pangenome assembly tools to address the unique complexities of plant genomes, will yield more accurate and comprehensive genomic analyses.

Acknowledgements

We thank Prof. Emma Mace (University of Queensland) for assistance with generation of simulated benchmarking dataset. We thank Dr. Cassandra Wattenburger (Bayer) for critical reading of the manuscript and helpful suggestions. This project was supported by the LOEWE Start Professorship from the Hessian Ministry of Higher Education, Research, Science and the Arts to AAG. VK was supported by GRK 2843 from the German Research Foundation (DFG).

Competing interests

The authors declare no competing interests.

Author contributions

Venkataramana Kopalli: Formal analysis; Methodology; Writing – original draft; Writing – review & editing. **Kübra Arslan**: Methodology. **Noemia Morales-Díaz**: Writing – original draft; Writing – review & editing. **Silvia F. Zanini**: Writing – review & editing. **Agnieszka A. Golicz**: Conceptualization; Funding acquisition; Methodology; Project administration; Supervision; Writing – original draft; Writing – review & editing.

Data availability

Code is available at https://github.com/KopalliV/Benchmarking_graph_pipelines

References:

1. Wang J, Yang W, Zhang S, Hu H, Yuan Y, Dong J, et al.. A pangenome analysis pipeline provides insights into functional gene identification in rice. *Genome Biol*. 2023; doi: 10.1186/s13059-023-02861-9.

2. Li F, Hu H, Xiao Z, Wang J, Liu J, Zhao D, et al.. Visualization and review of reads alignment on the graphical pan-genome with VAG. *bioRxiv*. 2023;

3. Tay Fernandez CG, Nestor BJ, Danilevicz MF, Marsh JI, Petereit J, Bayer PE, et al.. Expanding Gene-Editing Potential in Crop Improvement with Pangenomes. Int J Mol Sci.

4. Outten J, Warren A. Methods and Developments in Graphical Pangenomics. J Indian Inst Sci.

5. Zia K, Rao MJ, Sadaqat M, Azeem F, Fatima K, Tahir ul Qamar M, et al.. Pangenome-wide analysis of cyclic nucleotide-gated channel (CNGC) gene family in citrus Spp. Revealed their intraspecies diversity and potential roles in abiotic stress tolerance. *Front Genet*. 2022; doi: 10.3389/fgene.2022.1034921.

6. Zanini SF, Bayer PE, Wells R, Snowdon RJ, Batley J, Varshney RK, et al.. Pangenomics in crop improvement—from coding structural variations to finding regulatory variants with pangenome graphs. Plant Genome.

7. Wang W, Mauleon R, Hu Z, Chebotarov D, Tai S, Wu Z, et al.. Genomic variation in 3,010 diverse accessions of Asian cultivated rice. *Nature*. 2018; doi: 10.1038/s41586-018-0063-9.

8. Hirsch CN, Foerster JM, Johnson JM, Sekhon RS, Muttoni G, Vaillancourt B, et al.. Insights into the maize pan-genome and pan-transcriptome. *Plant Cell*. 2014; doi: 10.1105/tpc.113.119982.

9. Song JM, Guan Z, Hu J, Guo C, Yang Z, Wang S, et al.. Eight high-quality genomes reveal pan-genome architecture and ecotype differentiation of Brassica napus. *Nat Plants*. 2020; doi: 10.1038/s41477-019-0577-7.

10. Paterson AH, Bowers JE, Bruggmann R, Dubchak I, Grimwood J, Gundlach H, et al.. The Sorghum bicolor genome and the diversification of grasses. *Nature*. 2009; doi: 10.1038/nature07723.

11. Zhang LM, Luo H, Liu ZQ, Zhao Y, Luo JC, Hao DY, et al.. Genome-wide patterns of large-size presence/absence variants in sorghum. *J Integr Plant Biol*. 2014; doi: 10.1111/jipb.12121.

12. Wang B, Jiao Y, Chougule K, Olson A, Huang J, Llaca V, et al.. Pan-genome analysis in sorghum highlights the extent of genomic variation and sugarcane aphid resistance genes. bioRxiv.

13. Satish L, Shilpha J, Pandian S, Rency AS, Rathinapriya P, Ceasar SA, et al.. Analysis of genetic variation in sorghum (Sorghum bicolor (L.) Moench) genotypes with various agronomical traits using SPAR methods. *Gene*. 2016; doi: 10.1016/j.gene.2015.10.056.

14. Li H, Feng X, Chu C. The design and construction of reference pangenome graphs with minigraph. *Genome Biol*. 2020; doi: 10.1186/s13059-020-02168-z.

15. Bayer PE, Petereit J, Durant É, Monat C, Rouard M, Hu H, et al.. Wheat Panache: A pangenome graph database representing presence–absence variation across sixteen bread wheat genomes. *Plant Genome*. 2022; doi: 10.1002/tpg2.20221.

16. Garrison E, Guarracino A, Heumos S, Villani F, Bao Z, Tattini L, et al.. Building pangenome graphs. *bioRxiv*. 2023;

17. Yang Z, Guarracino A, Biggs PJ, Black MA, Ismail N, Wold JR, et al.. Pangenome graphs in infectious disease: a comprehensive genetic variation analysis of Neisseria meningitidis leveraging Oxford Nanopore long reads. *Front Genet*. 2023; doi: 10.3389/fgene.2023.1225248.

18. Hickey G, Monlong J, Ebler J, Novak AM, Eizenga JM, Gao Y, et al.. Pangenome graph construction from genome alignments with Minigraph-Cactus. *Nat Biotechnol*. 2023; doi: 10.1038/s41587-023-01793-w.

19. Skiadas P, Riera Vidal S, Dommisse J, Mendel MN, Elberse J, Van den Ackerveken G, et al.. Pangenome graph analysis reveals extensive effector copy-number variation in spinach downy mildew. Barber AE, editor. *PLoS Genet*. 2024; doi: 10.1371/journal.pgen.1011452.

20. Heller D, Vingron M. SVIM-asm: Structural variant detection from haploid and diploid genome assemblies. *Bioinformatics*. 2020; doi: 10.1093/bioinformatics/btaa1034.

21. Goel M, Sun H, Jiao WB, Schneeberger K. SyRI: finding genomic rearrangements and local sequence differences from whole-genome assemblies. *Genome Biol*. 2019; doi: 10.1186/s13059-019-1911-0.

22. Song B, Buckler ES, Stitzer MC. New whole-genome alignment tools are needed for tapping into plant diversity. Trends Plant Sci.

23. Yildiz G, Zanini SF, Afsharyan NP, Obermeier C, Snowdon RJ, Golicz AA. Benchmarking Oxford Nanopore read alignment-based insertion and deletion detection in crop plant genomes. *Plant Genome*. 2023; doi: 10.1002/tpg2.20314.

24. Tao Y, Luo H, Xu J, Cruickshank A, Zhao X, Teng F, et al.. Extensive variation within the pan-genome of cultivated and wild sorghum. *Nat Plants*. 2021; doi: 10.1038/s41477-021-00925-x.

25. Bolognini D, Sanders A, Korbel JO, Magi A, Benes V, Rausch T. VISOR: A versatile haplotype-aware structural variant simulator for short-and long-read sequencing. *Bioinformatics*. 2020; doi: 10.1093/bioinformatics/btz719.

26. Danecek P, Bonfield JK, Liddle J, Marshall J, Ohan V, Pollard MO, et al.. Twelve years of SAMtools and BCFtools. *Gigascience*. 2021; doi: 10.1093/gigascience/giab008.

27. Manni M, Berkeley MR, Seppey M, Zdobnov EM. BUSCO: Assessing Genomic Data Quality and Beyond. *Curr Protoc*. 2021; doi: 10.1002/cpz1.323.

28. Guarracino A, Heumos S, Nahnsen S, Prins P, Garrison E. ODGI: Understanding pangenome graphs. *Bioinformatics*. 2022; doi: 10.1093/bioinformatics/btac308.

29. Parmigiani L, Garrison E, Stoye J, Marschall T, Doerr D. Panacus: fast and exact pangenome growth and core size estimation. *bioRxiv*. 2024; doi: 10.1101/2024.06.11.598418.

30. Liao WW, Asri M, Ebler J, Doerr D, Haukness M, Hickey G, et al.. A draft human pangenome reference. *Nature*. 2023; doi: 10.1038/s41586-023-05896-x.

31. Li H. Minimap2: Pairwise alignment for nucleotide sequences. *Bioinformatics*. 2018; doi: 10.1093/bioinformatics/bty191.

32. Kirsche M, Prabhu G, Sherman R, Ni B, Battle A, Aganezov S, et al.. Jasmine and Iris: population-scale structural variant comparison and analysis. *Nat Methods*. 2023; doi: 10.1038/s41592-022-01753-3.

33. English AC, Menon VK, Gibbs RA, Metcalf GA, Sedlazeck FJ. Truvari: refined structural variant comparison preserves allelic diversity. *Genome Biol*. 2022; doi: 10.1186/s13059-022-02840-6.

34. Gel B, Díez-Villanueva A, Serra E, Buschbeck M, Peinado MA, Malinverni R. RegioneR: An R/Bioconductor package for the association analysis of genomic regions based on permutation tests. *Bioinformatics*. 2016; doi: 10.1093/bioinformatics/btv562.

35. De Coster W, De Rijk P, De Roeck A, De Pooter T, D'Hert S, Strazisar M, et al.. Structural variants identified by Oxford Nanopore PromethION sequencing of the human genome. *Genome Res*. 2019; doi: 10.1101/gr.244939.118.

36. Ou S, Su W, Liao Y, Chougule K, Agda JRA, Hellinga AJ, et al.. Benchmarking transposable element annotation methods for creation of a streamlined, comprehensive pipeline. *Genome Biol*. 2019; doi: 10.1186/s13059-019-1905-y.

37. Camacho C, Coulouris G, Avagyan V, Ma N, Papadopoulos J, Bealer K, et al.. BLAST+: Architecture and applications. *BMC Bioinformatics*. 2009; doi: 10.1186/1471-2105-10-421.

38. Shumate A, Salzberg SL. Liftoff: Accurate mapping of gene annotations. *Bioinformatics*. 2021; doi: 10.1093/bioinformatics/btaa1016.

39. Rice ES, Alberdi A, Alfieri J, Athrey G, Balacco JR, Bardou P, et al.. A pangenome graph reference of 30 chicken genomes allows genotyping of large and complex structural variants. *BMC Biol*. 2023; doi: 10.1186/s12915-023-01758-0.

40. Sirén J, Monlong J, Chang X, Novak AM, Eizenga JM, Markello C, et al.. Pangenomics enables genotyping of known structural variants in 5202 diverse genomes. *Science (1979)*. 2021; doi: 10.1126/science.abg8871.

41. Garrison E, Sirén J, Novak AM, Hickey G, Eizenga JM, Dawson ET, et al.. Variation graph toolkit improves read mapping by representing genetic variation in the reference. *Nat Biotechnol*. 2018; doi: 10.1038/nbt.4227.

42. : Simulating with a small sample. Issue #118 · lh3/minigraph · GitHub. https://github.com/lh3/minigraph/issues/118 Accessed 2024 Dec 4.

43. : Minigraph does not find variants in simulated data· Issue #119 · lh3/minigraph · GitHub. https://github.com/lh3/minigraph/issues/119 Accessed 2024 Dec 4.

44. Leonard AS, Crysnanto D, Mapel XM, Bhati M, Pausch H. Graph construction method impacts variation representation and analyses in a bovine super-pangenome. *Genome Biol*. 2023; doi: 10.1186/s13059-023-02969-y.

45. Zhao T, Eric Schranz M. Network-based microsynteny analysis identifies major differences and genomic outliers in mammalian and angiosperm genomes. *Proc Natl Acad Sci U S A*. 2019; doi: 10.1073/pnas.1801757116.

46. Morales-Díaz N, Sushko S, Campos-Domínguez L, Kopalli V, Golicz A, Castanera R, et al.. Tandem LTR-retrotransposon structures are common and highly polymorphic in plant genomes. 2024; doi: 10.21203/RS.3.RS-5356060/V1.

Figures

Figure 1: This flowchart illustrates the workflow used in our study to evaluate pangenome graph-building pipelines. Public sorghum data was used to generate simulated assemblies, which, along with real-world assemblies, were analyzed using pangenome graph-building pipelines and pairwise comparison tools. The resulting outputs were then assessed to evaluate their performance and accuracy.

Figure 2: Comparison of the number of nodes and edges for Minigraph-Cactus, PGGB, and Minigraph pangenome graphs constructed from simulated assemblies.

Figure 3: Comparison of F1 scores for non-redundant merged variant set of variants detected from all assemblies. SVIM-asm achieves the best performance with an F1 score of 0.93, followed closely by SyRI with 0.91. Minigraph-Cactus also performs well with a score of 0.89, while PGGB has the lowest score at 0.83.

Figure 4: F1-score comparison for individual samples. The comparison of F1 scores highlights differences in F1 score achieved for different samples. All pipelines exhibit similar performance trends.

Figure 5: UpSet plot displaying the intersection of structural variants (SVs) called by SyRI, SVIM, PGGB, and Minigraph-Cactus with the true set for simulated datasets. Each row corresponds to the set of SVs identified by a specific tool, represented by SyRI, SVIM-asm, PGGB, and Minigraph-Cactus. The horizontal bars on the left indicate the total number of SVs detected by each tool. Connected dots across rows represent intersections between the tools, indicating shared SV calls. The vertical bars above the plot show the size of each intersection, with higher bars corresponding to larger overlaps in SVs between the respective tools. The largest intersection, displayed on the far left, represents the SVs shared by all the tools.

Figure 6: Comparison of the number of nodes and edges for Minigraph-Cactus, PGGB, and Minigraph pangenome graphs built with real-world assemblies.

Figure 7: Comparison of the coverage percentages of each input assembly within the pangenome graph constructed using: (a) Minigraph-Cactus (b) PGGB (c) Minigraph. The percentage of each assembly included in the pangenome graph is represented by coloured bars, with light blue indicating the included portion and light green showing the excluded portion for each assembly.

Figure 8: Comparison of missing gene counts across different assemblies in a pangenome graph built with Cactus, PGGB, and Minigraph. Minigraph has the most missing genes in all assemblies.

Figure 9: Pie charts comparing the proportion of different variant types called by Minigraph-Cactus, PGGB, Minigraph, SVIM-asm, and SyRI. Minigraph and SVIM-asm focus on

structural variants and do not call SNPs or MNPs. The “Others" category includes complex variant types like duplications, inversions, and other structural rearrangements.

Figure 10: Overlap of variants in real-world assemblies. a) An UpSet plot showing the overlap of large variants (>50bp) between different methods, the bars represent the number of shared variants between dataset combinations, with the matrix below indicating which datasets are included. b) An UpSet plot showing the overlap of small variants (<50bp). c) Number of Multiallelic sites detected by each of the three pipelines.

Figure 11: Comparison of SVs with similarity to repeats for Minigraph-Cactus and PGGB; a) Total counts of structural variations (All SVs) and SVs with similarity to repeats detected by each method, including the count of SVs with similarity to repeats after applying 80% coverage filter b) Count of unique variants identified by Minigraph-Cactus and PGGB and the count of SV with similarity to repeats in those unique variants.

Figure 12: Comparison of alignment outcomes for reads simulated from Minigraph-Cactus and Minigraph graphs to corresponding graph and linear references. This figure compares the performance of VG Giraffe and Minimap2, on (a) Minigraph-Cactus (b) Minigraph. In the left panels, the x-axis shows the percentage of reads mapped by each aligner, with the y-axis representing the percentage of correctly mapped reads. Giraffe consistently outperforms Minimap2. The percent mapped starts from the percentage of reads mapped with MinQ 60 and progresses to MinQ 0. For instance, in (a) Minigraph-Cactus, Giraffe maps 38.27% of reads with MinQ 60, of which 38.15% are correct. In (b) Minigraph, Giraffe maps 74%, with 73.85% correct. In the right panels, the x-axis shows the minimum quality score (minQ), with the y-axis indicating the percentage of correct mappings. Giraffe maintains near-perfect excellent performance until a sharp drop at the highest thresholds, while Minimap's performance declines steadily.

Figure 13: Comparison of read alignment metrics for Minigraph-Cactus, Minigraph and Minimap2 for real-world reads. (a) displays the percentage of total reads successfully aligned by each tool: Minimap2 achieved the highest alignment rate at 82.47%, followed by Minigraph-Cactus with 73.44% and Minigraph with 73.27%. (b) shows the percentage of perfectly aligned reads, where Minigraph-Cactus outperformed the others with an alignment rate of 82.77%, Minigraph had 78.13% and Minimap2 only achieved 68.77%, highlighting the lower quality of read alignments to the linear reference (Minimap2) in (a) when compared to pangenome graph references (Minigraph-Cactus and Minigraph).

Click here to access/download
Supplementary Material
Supplementary_data.pdf
