# Supplementary material for: Toward a standardized framework for pangenome graph evaluation: assessing crop plant pangenome variation graph construction from multiple assemblies
Source: Gigascience. 2025 Dec 4;14:giaf121. doi: 10.1093/gigascience/giaf121 (PMC12676463; doi:10.1093/gigascience/giaf121)
Supplement: giaf121_GIGA-D-25-00090_Revision_1 [file giaf121_giga-d-25-00090_revision_1.pdf]

GigaScience

Towards a Standardized Framework for Pangenome Graph Evaluation: Assessing Crop Plant Pangenome Variation Graph Construction from Multiple Assemblies

--Manuscript Draft--

**Manuscript Number:**	GIGA-D-25-00090R1
**Full Title:**	Towards a Standardized Framework for Pangenome Graph Evaluation: Assessing Crop Plant Pangenome Variation Graph Construction from Multiple Assemblies
**Article Type:**	Research
**Funding Information:**	Deutsche Forschungsgemeinschaft (GRK 2843) — Dr. Agnieszka Golicz<br>Hessisches Ministerium für Wissenschaft und Kunst (LOEWE Start Professorship) — Dr. Agnieszka Golicz<br>Deutsche Forschungsgemeinschaft (497667402) — Dr. Agnieszka Golicz<br>European Social Fund (PRE2020-095111) — Ms Noemia Morales-Díaz
**Abstract:**	Pangenomes are crucial for understanding species-wide genetic diversity, delineating core and variable genes. This study compares three key pangenome graph assembly pipelines: Minigraph, PGGB, and Minigraph-Cactus, using publicly available Sorghum data. We introduce tailored metrics for comprehensive pangenome graph evaluation, including completeness, duplication levels and fidelity of structural variants.<br>By assessing the tools on Sorghum datasets, we gauge their efficacy in handling diverse genomic features. The analysis provides detailed insights into the strengths and limitations of Minigraph, PGGB, and Minigraph-Cactus, aiding researchers in informed tool selection. The metrics developed contribute to standardizing pangenome graph assessments, enabling robust and objective tool comparisons. We further demonstrate the utility of the metrics by applying them to pangenome graphs of three crops: soybean, barley and oilseed rape.<br>In conclusion, this benchmarking study advances our understanding of pangenome assembly tools and establishes a foundation for standardized evaluation metrics. We plan to further use these insights to optimize tool selection for specific applications, such as genome wide association studies, improving the accuracy of downstream analyses.
**Corresponding Author:**	Agnieszka Golicz<br>Justus Liebig University Giessen: Justus-Liebig-Universitat Giessen<br>Giessen, Hesse GERMANY
**Corresponding Author Secondary Information:**	
**Corresponding Author's Institution:**	Justus Liebig University Giessen: Justus-Liebig-Universitat Giessen
**Corresponding Author's Secondary Institution:**	
**First Author:**	Agnieszka Golicz
**First Author Secondary Information:**	
**Order of Authors:**	Agnieszka Golicz<br>Venkataramana Kopalli<br>Kübra Arslan<br>Noemia Morales-Díaz<br>Silvia Zanini
**Order of Authors Secondary Information:**	
**Response to Reviewers:**	Reviewer reports:

Powered by Editorial Manager® and ProduXion Manager® from Aries Systems Corporation

Reviewer #1: The authors provide a good summary of pan genome Graph evaluations based on Sorghum pan genome assemblies. They provide details insights into strengths and limitation for 3 pan-genome graph construction tools Mingraph, PGGN and Monograph-Cactus. They propose metrics to evaluate comprehensive pan genome graph evaluation, which could be applied or standardized of other pan genome graph construction methods.

Major comments:

* Introduction; the authors although highlight the importance of Pan-genomes and what value they offer. Could the authors also comment on why pan genome variation graph are important; specifically what advantages they offer over traditional linear based pan genomes.

Ans: Thank you for your feedback, we have now added a paragraph (Lines 84-94) in the Introduction highlighting the importance of variation graphs.

* Methods:
- Genome assembly and chromosome splitting-
- Although the authors have chosen accession that are diverse. Could they comment also on the quality of the assembly. For building graph-based pan genomes having high quality assemblies are crucial to avoid artificial bubbles or paths in the graphs that are built with the tools used for benchmarking. How much does the quality of assembly inflate the complexity of the pan-genome graphs built. Some background of the quality of the assembly would help in here to understand the benchmarking metrics that authors are testing

Ans: All selected genomes were assembled using both Illumina short reads and PacBio long reads, resulting in high-quality assemblies with contig N50 values up to 3.5 Mb and BUSCO completeness scores exceeded 95%, indicating strong representation of the gene space. (Added in lines 123-124)

- Simulated Genomes Assemblies-
- Can you clarify if the SV's used with VISOR for generating simulated assemblies are real or are the SV's provided simulated too?

Ans: Thank you, we have now clarified in the text that the SV’s used with VISOR are real SVs. (Line 135)

* Read Mapping
- Line 404 : Generating mapping indexes with PGGB pan genome graph was mentioned to be difficult due to the graph complexity and heavy computational demand. Could authors try read mapping with smaller subset graph and generate index for the smaller graph. Maybe reduce the graph to chr level?

Ans: Thank you for the suggestion. We revisited the task of generating mapping indexes for the PGGB pangenome graph. We were able to successfully build whole-genome mapping indexes, but the process required approximately 2.2TB of memory. The resulting index files were extremely large, and attempting to map reads back to them would be computationally expensive in most practical scenarios.
We also tested building indexes at the chromosome level, which was feasible with much lower resource requirements. However, the utility of individually indexed chromosomes for genome-wide analyses is limited (https://github.com/vgteam/vg/issues/3841).

- Line 417: what is correctly mapped reads? min_mapQ > 60 ?

Ans: Correctly mapped reads are simulated reads that align back to their original genomic position from where they were simulated from. ‘min_mapQ 60’ stands for a minimum mapping quality score of 60. This has now been clarified in the manuscript.

- Fig12: It was really hard to read and follow the figure and text in the main section. Suggest Figure 12 split as a,b,c, & d and refer them in the main text. Maybe also uses lines in the figure to show boundaries.

Powered by Editorial Manager® and ProduXion Manager® from Aries Systems Corporation

Ans: Thank you for the suggestion. We revisited Figure 12 and agree that the original layout was difficult to follow. To improve clarity and readability, we replaced it with a table (now Supplementary Table 1), which presents the data in a more accessible and organized format.

Minor comments

* Line 294: It should be mentioned as Figure 3 instead of Figure 2

Ans: Thank you, this has now been corrected.

* Suggest combining Fig 3, 4, & 5 as a facet plot for better readability and comparison

Ans: We combined Figures 3, 4, and 5 into a single facet plot, this updated figure is now presented as Figure 3

* Suggest combining Fig 6, 7, & 8 as a facet plot for better readability and comparison

Ans: We combined Figures 6, 7, and 8 into a single facet plot, this updated figure is now presented as Figure 4

* Figure 9 : Suggest using different choice for legends. Hard to distinguish blue and green categories. Also what is MNP ?

Ans: We have updated the figure with more distinguishable colours for the legend categories. MNP refers to Multiple Nucleotide Polymorphism, which involves two or more adjacent base substitutions occurring together.

* Line 435: Authors meant this as Mingraph instead of Minmap2

Ans: Thank you, this has now been corrected.

Reviewer #2: In this study, the authors compared three pangenomic methods Minigraph, PGGB, and Minigraph-Cactus using sorghum genome dataset. In general, I think this study was well-designed and the results could be extended in pangenomic studies of other plants. However, I also have the following comments for the authors to consider:

1.The authors only used sorghum as an example, which is biased, as the genome structure and data quality have significant impacts on pangenomic and downstream genetic analyses. I recommend the authors to use several representative plant species to compare the pangenomic pipelines and the generated evaluation metrics.

Ans: Thank you for this valuable suggestion. We have expanded our study to include three additional plant species Glycine max (soybean), Brassica napus (canola), and Hordeum vulgare (barley) which represent a diverse range of genome sizes, ploidy levels, and assembly qualities. By applying the same pangenome construction and variant calling pipelines across these species, we were able to evaluate the generalizability and robustness of the methods beyond Sorghum bicolor. The comparative analyses of graph sizes, completeness, duplication rates, and variant detection metrics reinforced the trends observed in sorghum. We have incorporated these results and related discussion into the manuscript to better reflect the broader applicability of the pangenome pipelines across diverse crop genomes.

2. The quality of the figures presented in this manuscript are low. Please use standard tools to regenerate the figures.

Ans: We have regenerated all figures using standard tools to ensure high resolution and clarity. The updated figures now have a resolution of 600 dpi, which significantly improves their quality.

3. Pangenome has been extensively studied in many plants, the authors should compare their conclusions with published ones.

Ans: Thank you for the suggestion. We added a section discussing published pangenome studies where assessment of pangenome graph-based approaches for variant discovery/genotyping was performed (Lines 548-567).

Powered by Editorial Manager® and ProduXion Manager® from Aries Systems Corporation

	We also added a section to the introduction discussing advantages of graphs and referring to studies, which used pangenome graphs (Lines 84-94).
**Additional Information:**	
**Question**	**Response**
Are you submitting this manuscript to a special series or article collection?	No
**Experimental design and statistics**<br><br>Full details of the experimental design and statistical methods used should be given in the Methods section, as detailed in our Minimum Standards Reporting Checklist. Information essential to interpreting the data presented should be made available in the figure legends.<br><br>Have you included all the information requested in your manuscript?	Yes
**Resources**<br><br>A description of all resources used, including antibodies, cell lines, animals and software tools, with enough information to allow them to be uniquely identified, should be included in the Methods section. Authors are strongly encouraged to cite Research Resource Identifiers (RRIDs) for antibodies, model organisms and tools, where possible.<br><br>Have you included the information requested as detailed in our Minimum Standards Reporting Checklist?	Yes
**Availability of data and materials**<br><br>All datasets and code on which the conclusions of the paper rely must be either included in your submission or deposited in publicly available repositories (where available and ethically appropriate), referencing such data using a unique identifier in the references and in the “Availability of Data and Materials”	Yes

Powered by Editorial Manager® and ProduXion Manager® from Aries Systems Corporation

section of your manuscript.<br><br>Have you have met the above requirement as detailed in our Minimum Standards Reporting Checklist?	
GigaScience has policies and guidelines in place for the use of generative AI-writing tools such as ChatGPT. If you have used such writing tools to assist with writing the manuscript this must be declared and cited in the text. Authors should not list AI-writing tools and other AI-assisted technologies as an author or co-author and should acknowledge that they are fully responsible for text generated or refined by AI-writing tools.<p><br><br>A summary of use (particularly in the introduction or among methods) needs to be included at the end of the paper, and the outputs should also be included as a supplementary file hosted in GigaDB or other open repositories. Please read our guidelines for more information. <p><br><br>By submitting to GigaScience, you are aware of the journal's AI-writing tools policy, and if you have declared use of such tools below, you have acknowledged this where appropriate in your manuscript and have made a summary of use and outputs available. <p><br><b>AI-assisted writing tools have been used in the preparation of this manuscript?	No

Powered by Editorial Manager® and ProduXion Manager® from Aries Systems Corporation

Manuscript
Click here to access/download;Manuscript;Ram.paper_postreview_final.doc

Towards a Standardized Framework for Pangenome Graph Evaluation: Assessing Crop Plant Pangenome Variation Graph Construction from Multiple Assemblies

Venkataramana Kopalli[1], Kübra Arslan[1], Noemia Morales-Díaz[2], Silvia F. Zanini[1], Agnieszka A. Golicz[1]

1. Department of Agrobioinformatics, IFZ Research Centre for Biosystems, Land Use and Nutrition, Justus Liebig University Gießen, Germany
2. Centre for Research in Agricultural Genomics, CRAG (CSIC-IRTA-UAB-UB) Campus UAB, Cerdanyola del Vallès, Barcelona, Spain

Agnieszka Golicz [0000-0002-9711-4826]; Venkataramana Kopall [0009-0000-1614-623X]i; Kübra Arslan [0000-0001-7975-518X]; Noemia Morales-Díaz [0000-0001-5258-486X]; Silvia Zanini [0000-0002-9137-8783];

Abstract

Pangenomes are crucial for understanding species-wide genetic diversity, delineating core and variable genes. This study compares three key pangenome graph assembly pipelines: Minigraph, PGGB, and Minigraph-Cactus, using publicly available Sorghum data. We introduce tailored metrics for comprehensive pangenome graph evaluation, including completeness, duplication levels and fidelity of structural variants.

By assessing the tools on Sorghum datasets, we gauge their efficacy in handling diverse genomic features. The analysis provides detailed insights into the strengths and limitations of Minigraph, PGGB, and Minigraph-Cactus, aiding researchers in informed tool selection. The metrics developed contribute to standardizing pangenome graph assessments, enabling robust and objective tool comparisons. We further demonstrate the utility of the metrics by applying them to pangenome graphs of three crops: soybean, barley and oilseed rape.

In conclusion, this benchmarking study advances our understanding of pangenome assembly tools and establishes a foundation for standardized evaluation metrics. We plan to further use these insights to optimize tool selection for specific applications, such as genome wide association studies, improving the accuracy of downstream analyses.

Introduction

Pangenomics, a field focused on capturing the complete genetic diversity within a species, has gained significant attention in genomics, agriculture, and microbial studies [1]. Pangenomes offer a more comprehensive view of genetic variation within populations, species, or genera by moving beyond the limitations of a single reference genome, such as its failure to represent the full genetic diversity, introducing reference bias, and missing structural variants [2]. Pangenomes are structured collections of genomic data that enable the study of genetic variant relationships while preserving the continuity of sequences and structural variations across individuals, allowing for the identification of complex DNA polymorphisms like structural variations (SVs), copy number variations (CNVs), and presence/absence variants (PAVs) [3,4].

Pangenomes have been applied in plant research to understand genetic mechanisms underlying trait variation, accelerate breeding processes, and improve crop performance [5]. Pangenome allows for the capture of genetic diversity essential for enhancing agronomical traits by incorporating wild species and multiple varieties and have been instrumental in studying transposable elements and accessory genomes, shedding light on their impact on genetic variation [6]. Pangenome analysis has been particularly valuable in crops like rice (*Oryza sativa*), maize (*Zea mays*), and rapeseed (*Brassica napus*), where it has revealed previously unknown genetic variations, aiding in accelerated genetic improvement [7–9].

Sorghum's significance for pangenome research lies in its genomic characteristics in addition to its applications in food, feed, or biofuel. Sorghum has a relatively small (~730 Mb) diploid genome (2n=20) which simplifies genome assembly. Repeat content constitutes approximately 61% of its genome, significantly influencing its structural variation (SV) [10]. Studies have identified extensive single nucleotide polymorphisms (SNPs), indels, and large-size presence/absence variants (PAVs) that contribute to sorghum's genetic diversity, an important factor for breeding [11]. Additionally, the pangenome approach in sorghum has revealed crucial structural variations, especially in genes related to stress responses, resistance to diseases, and adaptation to various environments. Known structural variants include numerous large-size PAVs and mobile elements, which further diversify sorghum's genome. This genetic variability is valuable for understanding the evolutionary dynamics and guiding crop improvement strategies [12,13].

There are several tools and pipelines that enable the creation of pangenome variation graphs, which are a graph-based representation of multiple genomes. These graphs model genetic diversity by encoding genomic sequences as nodes and their relationships, such as continuity

or variation (e.g., SNPs, insertions, deletions, and structural variations), as edges. This allows for the efficient comparison, alignment, and analysis of genetic variation within a population. Minigraph, PGGB and Minigraph-Cactus are among these tools and pipelines used for these purposes. Minigraph, PGGB, and Minigraph-Cactus were selected as they are among the most widely used and well-regarded tools for constructing pangenome graphs, offering robust and complementary capabilities. Minigraph is a versatile tool designed to construct and manipulate sequence graphs, is particularly effective in handling large structural variations (SVs) and is optimized for speed and scalability. It constructs pangenomes by aligning multiple genome assemblies against a reference, facilitating the detection of large insertions, deletions, and complex rearrangements [14] and was used to build the pangenome graph database representing presence–absence variation across sixteen bread wheat genomes [15]. The PanGenome Graph Builder (PGGB) is an innovative, reference-free pipeline designed to construct unbiased pangenome graphs. By utilizing all-vs-all whole-genome alignments and advanced graph embeddings, PGGB builds and continuously refines a model that allows for the identification of genetic variation, assessment of conservation, detection of recombination events and inference of phylogenetic relationships [16]. It was utilized to build a pangenome graph for a comprehensive genetic variation analysis of *Neisseria meningitidis* [17]. Minigraph-Cactus pipeline combines two methodologies: Minigraph and Cactus. Minigraph excels in building compact and accurate graphs from multiple genomes by aligning reads to a reference graph, while Cactus enhances this by integrating these graphs into a comprehensive pangenome structure. This combination facilitates both visualization and exploration of genetic variation, structural differences, and evolutionary relationships across diverse genomes, offering valuable insights into genomic diversity and complexity [18]. This pipeline was successfully utilized to build a pangenome graph and reveal extensive effector copy-number variation in spinach downy mildew [19].

Unlike linear pangenomes, which aim to encode all variation in a linear data structure built around a reference genome [1,20], pangenome variation graphs encode multiple haplotypes, alleles, and structural variations directly into the graph topology[15,21,22]. As a result, these graph-based models preserve complex variant relationships and allow for improved alignment and variant discovery across divergent genotypes. This is particularly advantageous for crops like sorghum, where large structural variations, presence/absence variants, and repetitive content are prevalent. Graph-based approaches can reduce false negatives in SV detection, improve read mapping accuracy in structurally complex regions, and enable downstream

analyses such as graph-based genotyping and structural variant and haplotype-aware association studies[23–25] As such, pangenome graphs offer a powerful alternative to linear [22]approaches for capturing true biological diversity in plant genomes [26,27].

SV detection and comparative genomics have traditionally relied on linear reference-based approaches to identify genomic differences between individual assemblies. To fully understand the benefits of a pangenome based approach, we compared Minigraph, PGGB and Minigraph-Cactus with two linear SV callers, SVIM-asm and SyRI. SVIM-asm is a linear variant caller designed specifically for long-read assemblies, enabling the accurate detection of complex structural variants, such as insertions, deletions, duplications, and translocations, by leveraging the detailed structural information encoded in such assemblies [28]. SyRI is a comprehensive tool for predicting genomic differences between related genomes using whole-genome assemblies (WGA). The assemblies are aligned using whole-genome alignment tools, and these alignments are then used as input to SyRI. SyRI identifies syntenic path (longest set of co-linear regions), structural rearrangements (inversions, translocations, and duplications), local variations (SNPs, indels, CNVs, etc) within syntenic and structural rearrangements, and un-aligned regions [29]. In this manuscript we report extensive comparisons of the three leading pangenome variation graph construction pipelines using simulated and real-world data representing a sorghum diversity set. We assess the properties of the resulting pangenome graphs in terms of their completeness, encoded variation, but also suitability for downstream applications including read mapping. Our work expands the knowledge base and toolbox necessary to build high quality pangenome graphs in plants and especially crops, which often boast genomes affected by high levels of duplication, sequence diversity and transposable element activity [30,31].

Methods and Tools

This study employed a comprehensive workflow encompassing genome assembly, chromosome splitting, simulated genome generation, pangenome construction, variant calling, and downstream analyses. The workflow, as shown in Figure 1, provides an overview of the key steps, tools, and methods used across the study, linking each stage of the analysis pipeline.

Genome Assembly and Chromosome Splitting

Six assemblies were chosen based on the phylogenetic relationships published in a study by Tao et al [32]. We chose the accessions to be as diverse as possible to capture extensive

variation. All six sorghum assemblies used were generated using a hybrid sequencing approach and exhibit high contiguity (contig N50 up to 3.5 Mb) and completeness (>95% BUSCO). We included two *S. bicolor* (IS8525 and Rio), one *S. bicolor verticilliforum* (AusTRCF317961), one *S. bicolor drummondii* (PI532566), one *S. bicolor margaritiferum* (IS19953), and an outlier *S. propinquum* (S369-1). Publicly available sorghum genome assemblies were downloaded. [32]. Similarly, publicly available assemblies (Supplementary Table 7) for Rapeseed [9] , Barley [33] and Soybean [24] included in this study were obtained. The reference genomes used were Darmor v10 [34] for Rapeseed, MorexV3 [35] for Barley and Glycine max v4.0 [36] for Soybean. The assemblies were then split into individual chromosome files using a shell script.

Simulated Genome Assemblies

Simulated genomes for all six accessions were generated using VISOR (Version 1.1.2.1) [37], with *Sorghum bicolor* v3.0.1 as the reference backbone, incorporating real-world structural variants (SVs) identified in the six accessions. A filtering step was applied to retain only those SVs present in at least two of the six assemblies due to a large number of variants unique to one genotype prior to genome simulation. After filtering, 36,358 SVs remained, and all those exceeding 100 bp in size were used to simulate assemblies.

The headers in the FASTA files of the genome assemblies were modified to include the genome name as a suffix to the chromosome name. This adjustment simplified tracking individual assemblies and ensured efficient downstream analysis.

Pangenome Construction

Pangenomes were constructed per chromosome for the 10 chromosomes of *Sorghum bicolor* using PGGB, Minigraph-Cactus, and Minigraph for real-world and simulated datasets separately. The methods of construction were the same for both datasets.

PGGB (Version 0.5.4)

For each of the chromosomes, we created a FASTA file containing the corresponding seven sequences, six from the selected accessions and one from the reference. We compressed the resulting FASTA and created an index (.fai) file using samtools (Version 1.18) [38] faidx. FASTA and index files were used as input for graph construction, running PGGB with default parameters (k=19, p=90, s=5000). The order of the input samples was deemed insignificant as PGGB performs an all-vs-all alignment.

Minigraph-cactus (Version 2.7.2)

The Minigraph-Cactus tool was used with default parameters, using the *Sorghum bicolor* v3.0.1 as the reference along with the six genome assemblies. The command was run with –gfa, –viz, –draw, –og, and –gbz parameters. Minigraph-Cactus calculates the mash distance, which is a metric used to estimate the genetic distance or similarity between two genome assemblies and reorders the input assemblies by placing the reference assembly first, followed by the remaining assemblies in decreasing order of mash distance to the reference.

Minigraph (Version 0.21)

The Minigraph pangenome was constructed for each chromosome of the assemblies with the *Sorghum bicolor* v3.0.1 reference as a backbone. Default parameters were used. Since Minigraph does not provide instructions for determining the order of input samples, a random order was used, assuming that the order is insignificant.

BUSCO (Version 6.0.0)

We assessed the completeness of the newly generated graphs with BUSCO, a bioinformatics tool that scores genome assemblies, gene sets, and transcriptomes by comparing them against a set of highly conserved orthologs. BUSCO evaluates the presence and quality of expected single-copy orthologs in the given dataset, providing insights into the completeness and potential gaps in the assembly [39].

We used the odgi flatten command from odgi tools (Version 0.8.6-0-ge647844f) [40] to convert the OG files of pangenomes of each chromosome into FASTA format and merged all the resulting FASTA files to reconstitute full assemblies. We ran BUSCO with the poales_odb10 lineage dataset and genome mode to assess the completeness of the pangenome.

Panacus (Version 0.2.3)

Panacus is a tool designed for quantifying the core size and estimating growth curves in pangenome graphs. It calculated several key statistics, including pangenome growth and core curves, by counting nodes, edges, and base pairs, and tracking how they were covered across paths. This tool enabled the grouping of paths, focusing on specific regions, and generated clear, interactive reports [41].

To analyze pangenome graphs, we first selected the paths corresponding to haplotypes by filtering and excluding specific references. We then used Panacus to calculate the coverage and

pangenome growth for nodes with varying coverage and quorum thresholds (1/0, 2/0, 1/1, 1/0.5, and 1/0.1) using up to 4 threads. The resulting data was output as a TSV file. Finally, coverage histograms and pangenome growth curves were visualized, including estimated growth parameters (Supplementary Figure 2).

Variant Calling and filtering

PGGB and Minigraph-cactus

PGGB and Minigraph-Cactus variants were called with VG deconstruct (Version 1.54.0)[42]. Variants were called with respect to the path of the reference genome, along with parameters to process all snarls (-a) and to only consider traversals that corresponded to paths in the graph (-e). The input file was the GFA file output of PGGB, and this process was repeated for each chromosome-graph. The variants were filtered to include only variants with alternative alleles, and the multiallelic variants were split using bcftools norm (Version 1.16-23) [38] to facilitate downstream analysis.

Minigraph

We added path information (P-lines) to Minigraph's GFA by manually curating the output of minigraph -call, which retraced the assembly's path. We then called the variants with VG deconstruct.

SVIM-asm (Version 1.0.3)

SVIM-asm is a structural variant caller for haploid or diploid genome-genome alignments. We ran SVIM-asm separately for each assembly against the *Sorghum bicolor* v3.0.1 reference as it calls SVs from pairwise assemblies aligned with Minimap2 (Version 2.26-r1175) [43]. SAM outputs were converted to BAM using samtools, then BAM files were sorted and indexed to be used as inputs to SVIM-asm in haploid mode to call the variants.

SyRI (Version 1.6.3)

We used SyRI to compare chromosome-level assemblies and identify structural rearrangements and synteny. The same pairwise alignments generated with Minimap2 for SVIM-asm were used as input for SyRI variant calling. The initial VCF file produced by SyRI was missing genotype information and flagged 'N's as Highly Diverged Regions (HDRs). We resolved this by filtering out the 'N's, manually adding FORMAT and SAMPLE columns, and updating the VCF header for compatibility with bcftools. After merging the individual VCF files for each method with

Jasmine (v1.1.5) [44], we refined the results by sorting using bcftools sort and splitting multiallelic variants using bcftools norm.

Truvari Bench (Version 4.1.0)

To assess the accuracy of both variant callers, we calculated and compared the precision, recall, and F1 scores of called variants from the pangenomes against the true set of variants from Tao et al., 2021 which was used to simulate the genome assemblies. Truvari bench was run with default parameters [32,45].

regioneR (Version 1.36.0)

We used permutation testing with the regioneR package to evaluate the performance of PGGB and Minigraph-Cactus variant calling with real-world assemblies [46]. It enabled for the assessment of overlaps between genomic regions by comparing observed data against a distribution generated from randomized genomic regions to determine statistical significance. We filtered out pangenome variants longer than the longest variant in the true set, as their inclusion disproportionately increased the expected overlap during the regioneR permutation analysis. Longer variants had a higher likelihood of overlapping by chance due to their size, which affected the randomization process and inflated the baseline expected overlap. The permutation test was conducted using the permTest function, with 100 permutations of genomic regions. The randomizeRegions function was employed to generate these randomized regions, and the numOverlaps function was used to evaluate the overlap between the variant sets and the true variants. We set count.once=TRUE to ensure that each true-set variant was counted only once, avoiding overestimation from multiple overlaps with the same pangenome variant.

Surpyvor (Version 0.5)

Surpyvor is a Python package designed for analyzing structural variants (SVs) enabling for the visualization and quantification of SV relationships within genomic data [47]. We utilized the venn function for creating Venn diagrams and the upset function for generating UpSet plots. For large variant comparisons, we used the default parameters; however, for small variant comparisons, we used the -snv option to override the default SURVIVOR method and opted for bcftools instead, as the SURVIVOR method was primarily tailored for large SV analyses.

Repeat content Analysis

To assess repeat content in the pangenome variants, we first generated a transposable element (TE) library using the EDTA (Extensive de novo TE Annotator) pipeline (Version 2.1.1) [48] EDTA was applied to generate a non-redundant high-quality TE library. This library was then formatted as a BLAST database with makeblastdb to allow for efficient sequence comparison. Variant sequences from the VCF file were extracted and formatted in FASTA, with each sequence labelled by its genomic coordinates for unique identification. Sequence headers were standardized to remove any non-compatible characters to facilitate downstream analysis. We then used BLASTN (Version 2.12.0) [49] to align these variant sequences against the TE database, retrieving only the top hit for each variant. The results were sorted by query coverage, and only the best alignment for each variant was retained. We further filtered the results to include only those matches with a query coverage of 80% or higher to ensure high confidence in the repeat classification. This approach enabled us to reliably identify and characterize repeat-associated variants within the pangenome.

Missing genes and assembly coverage statistics

To detect missing genes across assemblies in the pangenome graph, we first generated FASTA sequences for each assembly's path in the graph using odgi paths. Gene annotations from the reference genome were mapped to each pangenome assembly FASTA with Liftoff (Version 1.6.3) [50], identifying unmapped genes as potentially missing. Each assembly was also aligned to its path in the pangenome graph using Minimap2. The alignments were processed with samtools to generate coverage statistics.

Read Simulation and Mapping

We evaluated the performance of linear and graph mapping by using simulated reads, which were generated using the code developed by Rice et al., 2023 [51]. This process involved three main steps: first, simulating reads from the graph; second, aligning those reads to both the graph using VG Giraffe (Version 1.58.0) [52] and to the *Sorghum bicolor* v3.0.1 linear reference using Minimap2, and finally, comparing the results from both mapping methods based on the alignment quality and correctness rates.

Individual OG files generated for each chromosome were merged with odgi squeeze. The merged OG file was converted back to GFA format with odgi view.

Read Simulation: We simulated one million reads from the corresponding pangenome graphs (which were then used as reference for read mapping) using VG sim [53]. The length of each

simulated read was set to 150 bps, the error rate was set at 0.24%, the indel rate was set to 0.029%, the mean insert size was set to 570 bps, and the standard deviation of the insert size was set to 165. The resultant GAM file was converted to FASTQ format with VG view.

Mapping to VG Giraffe: The simulated reads were mapped back to the graph using VG Giraffe. To enable VG Giraffe to map reads to the graph, indexes were generated using VG autoindex with the workflow parameter set to Giraffe, which produced the necessary files for mapping (.gbz, .min, and .dist). We annotated the GAM file and compared it to the simulated reads using VG gamcompare, generating cumulative alignment and correctness rates.

Mapping to Linear reference: We extracted reference paths from the graph using VG paths, mapped the simulated reads with Minimap2, and injected the alignment back into the graph. We annotated the GAM file and compared it to the simulated reads using VG gamcompare, generating cumulative alignment and correctness rates.

Real-world reads: We downloaded paired-end reads datasets of sorghum [32] to test mapping efficiency in a real-world scenario. Reads were mapped to the graph using VG Giraffe and to the linear reference using Minimap2, with the same parameters as the simulated reads.

Additional Species Benchmarking

To evaluate the generalizability of our benchmarking results and minimize species-specific bias, we extended our pangenome construction and evaluation pipeline to three additional plant species: *Glycine max* (soybean), *Brassica napus* (Rapeseed), and *Hordeum vulgare* (barley). These species were selected to represent a diversity of genome sizes, ploidy levels, and assembly qualities. For each species, we applied the same pangenome construction methods (PGGB, Minigraph-Cactus, and Minigraph) using publicly available real-world genome assemblies. All steps, including chromosome-wise graph construction and downstream analysis, were performed as described for *Sorghum bicolor*, but only using real-world assemblies.

K-mer-based analysis of sequence duplication in graphs

Along with evaluating BUSCO scores for these crops, we also implemented an additional method to estimate duplication levels in the pangenomes, which was designed to overcome limitation of fragmentation of BUSCO genes by the graph structure. For each crop, we first performed BUSCO analysis on the reference genomes (Supplementary Table 3) and extracted the coding sequences (CDS) corresponding to 'Complete' BUSCOs. We then generated 21-mers

from these CDS regions and aligned them using bwa aln [54] to both the input assemblies used for pangenome construction. We retained only those k-mers, that uniquely mapped to all input genomes by filtering the alignments for the 'X0:i:1' tag in the SAM files. These k-mers were then mapped to the reference genome and the corresponding flattened graph FASTA. By analysing the proportion of uniquely (X0:i:1) and multiply mapped k-mers in these alignments, we assess the extent of duplication across the pangenome graphs.

Results

Simulated Assemblies

Pangenome size and content

The size of the pangenomes was similar between pipelines, while the number of nodes greatly varied across methodologies. The PGGB pangenome consisted of 851 Mb with ~190,000 nodes, the Minigraph-Cactus pangenome was 795 Mb with ~220.000 nodes, and the Minigraph pangenome was 878 Mb with only 950 nodes (Figure 2, Supplementary Table 5b). The linear reference genome had a size of 688 Mb, demonstrating that all pangenomes exhibited an increase in total genomic content compared to the linear reference (Supplementary Table 5b). This highlighted the additional complexity and variation encompassed by the pangenome graph. Minigraph stood out in our simulation, as it failed to detect variants which resulted in a smaller number of very long nodes. The average node length for Minigraph was 968,806 bp, which was very high compared to PGGB's 4,517 bp and Minigraph-Cactus' 3,714 bp. The mean BUSCO completeness for the six input simulated assemblies was 98.4% with 1.6% duplication comparable to BUSCO completeness of 98.4% with 1.7% duplication seen in the three pangenomes. (Supplementary Table 3b).

Variant calling

To assess the accuracy of variant calling we calculated F1 scores for variants identified from pangenomes constructed with simulated assemblies of Minigraph, PGGB, and Minigraph-Cactus. This comparison was made against a set of true variants from a published dataset using the Truvari benchmarking tool. The average F1 score for PGGB was 0.83, while Minigraph-Cactus achieved a higher average F1 score of 0.89 (Figure 3a). Minigraph detected a considerably low number of variants in a simulated scenario, an unexpected behaviour also reported by other users [55,56]. The results were consistent when cross-checking the Minigraph

output with the Minigraph output from the Minigraph-Cactus pipeline. The limited number of variants detected by Minigraph prevented the calculation of a meaningful F1 score and the output did not provide sufficient data for reliable evaluation.

The variants were also called using linear-based SVIM-asm and Syri (Figure 3a) to compare the variant calling efficiency of graph-based PGGB, Minigraph-Cactus and Minigraph. The F1 score comparisons across all assemblies suggested that pairwise comparison methods outperformed pangenome graph pipelines in the SV calling task. SVIM-asm achieved the highest overall F1 score of 0.93, followed by SyRI at 0.91, both outperforming Minigraph-Cactus (0.89) and PGGB (0.83) (Figure 3a). SVIM-asm and Syri also exhibited higher consistency and F1 scores across multiple genome assemblies (Figure 3b).

Pairwise variant callers offer slightly better variant detection accuracy and robustness across different genome assemblies compared to pangenome construction pipelines, as shown in Figure 3b in a simulated scenario. Nevertheless, the vast majority of SVs were detected across all methodologies (Figure 3c), demonstrating the suitability of graph-based approaches while revealing differences in sensitivity and specificity across tools.

Real-World assemblies

Pangenome size and content

In addition to simulations, we also constructed pangenome graphs using six assemblies representing real-world data. Pangenome graph sizes varied between the three pipelines, with the PGGB pangenome reaching 4.7 Gb, the Minigraph-Cactus pangenomes at 4.2 Gb, and the Minigraph pangenomes being considerably smaller at 920 MB. For comparison, the size of the linear reference genome was 688 Mb, indicating that all pangenomes represented an increase in total genomic content relative to the linear reference, reflecting the added complexity and variation captured by the pangenome graph. The number of nodes and edges in the graph corresponds to its size, as shown in Figure 4b (Supplementary Table 5a). Size differences between Minigraph and the other two graphs is due to the inclusion of only variants > 50bp, while PGGB and Minigraph-Cactus do not have a size filter. When compared to the graph sizes of simulated assemblies, the size and content of real-world pangenomes increase for PGGB and Minigraph-Cactus, consistent with the presence of many additional short variants (SNPs and InDels) which were not part of simulations.

The input real-world assemblies had a mean BUSCO completeness of 95.4% with 2% duplication. Pangenomes built with Minigraph-Cactus, PGGB and Minigraph had BUSCO completeness of 94.5% with 16.5% duplication, 98.7% with 16% duplication and 99.4%% with 2.7%% duplication, respectively (Supplementary Table 3a). The reduction in completeness scores is expected, as in some cases genes will be broken up across multiple nodes. The duplication levels point to a successful genomic data compression, which is one of the key tasks of pangenome graph construction. To address the issue of genes broken up across nodes, which became more pronounced as other species were included in the analysis, we devised a new, k-mer based metric, where k-mers derived from BUSCO genes identified in the reference genome were subsequently mapped to the flattened graph FASTA to assess the completeness and duplication levels. The k-mer based analysis painted a similar picture of slightly elevated duplication rates in Minigraph-Cactus and PGGB graphs (Supplementary Table 4).

We tracked the growth and coverage statistics of graphs using Panacus. Growth helps track the accumulation of genes and genomic features, while coverage statistics assess the phylogenetic diversity represented. Overall, all three pangenome pipelines show similar graph expansion dynamics, with less nodes added with each new sample (Supplementary Figure 2-5).

Pangenome construction can lead to data loss, when sequences present in the input assemblies are not included in the pangenome graph. We evaluated the representation of each input assembly within the pangenome graph across all three pangenome pipelines. We observed that all assemblies were represented with a coverage above 94% in both the Minigraph-Cactus (Figure 4a,c) and PGGB (Figure 4b,c) pangenomes. When assessing gene presence in regions present in the assembly but missing from the graph for each assembly, we found that one gene from assembly AusTRCF317961, four genes each in IS19953 and IS8525, 14 genes in PI532566, three genes in Rio and 25 genes in S369-1 were not found in the assemblies recovered from the graph (Figure 4a). These numbers were similar in both Minigraph-Cactus and PGGB pangenomes, but Minigraph showed comparatively lower proportion (averaging 83%) of assemblies included in the graph (Figure 4c) and consequently a higher number of missing sequence and genes (Figure 4a) (Supplementary Table 6).

A similar trend was observed in the additional species included in this study. For soybean, rapeseed, and barley, pangenome graphs constructed using PGGB and Minigraph-Cactus were substantially larger than those built with Minigraph, consistent with patterns observed in sorghum. For instance, in soybean, graph sizes were approximately 5.1 Gb (PGGB), 4.2 Gb

(Minigraph-Cactus), and 1.1 Gb (Minigraph), with similar pattern observed for rapeseed and barley (Figure 5a; Supplementary Table 5). Node count distributions mirrored these differences across species (Figure 5b–d). Minigraph graph had the highest proportion of sequence and genes missing (Supplementary Table 6). The BUSCO k-mer duplication analysis using 21-mers derived from reference BUSCO genes revealed similar patterns as observed in Sorghum. The Minigraph-Cactus and PGGB had higher duplication rate than the Minigraph graph, but overall, showed good ability to reduce the level of sequence redundancy (Supplementary Table 4).

Variant Calling

We performed a permutation test for variants called from pangenomes (Minigraph-Cactus, PGGB and Minigraph) constructed with real-world assemblies and also and pairwise variant callers (SVIM-asm and SyRI) to compare them with previously reported SVs in sorghum [32]. This test, conducted using the regioneR package, involved generating 100 randomizations of genomic regions to assess the significance of the observed overlaps between pangenome variant sets and the previously reported variant set. All three pipelines, Minigraph-Cactus, PGGB and Minigraph, demonstrated statistically significant results with a permutation count of 100 iterations and randomization using randomizeRegions, yielding a p-value of 0.0099 and high Z-score (Supplementary Figure 1a-c). These findings indicate that for real-world data, the SVs found using a pangenomic approach overlap previously reported variants much more than would be expected by chance alone. Pairwise variant callers, SVIM-asm and SyRI, also show significant overlap, though to a lesser degree than the pangenome pipelines (Supplementary Figure 1d,e). Together, with the results from simulations, this suggests that building a pangenome graph using Minigraph-Cactus and PGGB is a viable approach for variant calling.

We analysed the proportion of variant types identified by each tool and observed that Minigraph-Cactus, PGGB and SyRI identify variants of all types and have similar proportions of variant distribution. They also include an 'others' category for complex or less common variant types, including duplications, inversions, and other structural rearrangements (Figure 6).

While these results confirm the quality of variants identified by pangenome methods, we also evaluated the overlap of variants between pangenome pipelines and pairwise variant callers (Figure 7a). Notably, PGGB and Minigraph-Cactus exhibit higher overlap with each other compared to pairwise callers SVIM-asm and Syri. We assessed the performance of all tools in

detecting small variants in real-world assemblies, focusing on Indels under 50bp including MNPs (Multiple Nucleotide Polymorphism) and SNPs (Figure 7b). Minigraph-Cactus detected approximately 7.7 million SNPs and 2.3 million small Indels, while PGGB identified around 7.6 million SNPs and 2.6 million small Indels. Pairwise variant caller SyRI detected 3.2 million SNPs and 0.93 million small Indels while SVIM-asm detected no SNPs and 11k small Indels which is relatively small, resulting in a reasonable overlap between PGGB, Minigraph-Cactus and SyRI but negligible overlap of variants across all four methods (Figure 7b) (Supplementary Table 2). Minigraph-Cactus and PGGB identified 0.59 million and 0.56 million multiallelic sites, respectively, while Minigraph only found 46k multiallelic sites (Figure 7c).

We also analyzed the similarity of variants called by PGGB and Minigraph-Cactus to repeats by comparing them to the *S. bicolor* repeat library. We found that 28% of variants in both PGGB and Minigraph-Cactus matched repeats. When only matches with over 80% coverage were considered, these results were reduced to 7.5% for PGGB variants and 11% match for Minigraph-Cactus variants (Figure 8a). Both Minigraph-Cactus and PGGB call a high number of variants unique to each of the pipelines (Figure 8b). We also analysed repeat similarity of those unique variants and found that 48% of PGGB unique variants and 38% of Minigraph-Cactus unique variants matched with repeat locations (Figure 8b).

For the additional species, Soybean, Rapeseed and Barley, we performed variant calling for the same pangenome pipelines (Minigraph-Cactus, PGGB, and Minigraph) on real-world assemblies, along with SVIM-asm and SyRI. We quantified the total number and types of variants identified by each pipeline to provide a comparative overview of variant detection across species (Supplementary Table 2, Supplementary Figures 6-8). Variant counts ranged, with a similar distribution of SNPs, small indels, and multiallelic sites observed as in *Sorghum bicolor* (Supplementary Table 2).

Read Mapping

One of the key objectives of a pangenome graph is to act as a reference for read mapping. We therefore assessed mapping outcomes for graphs produced with different pipelines. We were unable to generate mapping indexes for the PGGB pangenome graph due to heavy computational and memory requirements and hence were unable to test it for read mapping. This issue has been previously reported during chicken pangenome analysis [51]. Following the approach of Rice et.al., 2023, we evaluated the performance of linear and graph-based mapping by simulating reads from the entire pangenome graph, which includes all seven

assemblies (six input assemblies and the *Sorghum bicolor* v3.0.1 reference), rather than just from a single reference genome. The simulated reads were then mapped to both the graph using VG Giraffe and the *Sorghum bicolor* v3.0.1 linear reference using Minimap2. This allowed us to assess the mapping performance across the genetic diversity captured in the pangenome. For Minigraph-Cactus, the Giraffe results show better mapping compared to the corresponding Minimap2 alignments (Supplementary Table 1a). Specifically, at a high minimum mapping quality (min_mapQ) threshold of 60, although Giraffe (Minigraph-Cactus) only maps 38.27% it achieves a correct read mapping rate of 99.68%, substantially better than Minimap2's, which maps 48.67% reads with only 58.18% reads mapped correctly (Supplementary Table 1a). Correctly mapped reads are simulated reads that align back to their original genomic position from where they were simulated from. Giraffe (Minigraph-Cactus) maintains a high rate (over 98%) of correctly mapped reads, whereas Minimap2's has lower proportion of correctly mapped read at similar thresholds (Supplementary Table 1a). At min_mapQ 1, the percentage of total reads aligned correctly is 79.60%, when compared to Minimap2's 35.49% (Supplementary Table 1a). This demonstrates that Giraffe using the Minigraph-Cactus pangenome offers substantial improvements in both mapping accuracy and total alignment correctness compared to linear-based approaches. It is notable that despite simulating the reads from the same pangenome graph, some reads did not align to the graph at mapQ 1, which likely reflects alignment ambiguity in highly repetitive regions as the mapQ 1 threshold may exclude low-confidence alignments, contributing to the reduced alignment rate.

Giraffe performs well also with the Minigraph pangenome. At a min_mapQ threshold of 60, Giraffe (Minigraph) maps 74.62% of reads with 95.08% correct read mapping rate, which is better than the corresponding Minimap2's mapping of 58.93% reads with 92.78% correct read mapping rate. The correctness rate for Giraffe (Minigraph) remains high at around 98%, similar to the results with Minigraph-Cactus. Furthermore, the percentage of total reads aligned correctly with Giraffe (Minigraph) reached 91.91% at min_mapQ 1, when compared to Minimap2's 65.74% (Supplementary Table 1a), highlighting its superior mapping ability compared to both Minigraph-Cactus and the linear reference. The comparatively good read mapping performance of Minigraph-only graph can be attributed to much lower graph complexity due to the absence of small variants, which makes read simulation and mapping task much more straightforward. Minigraph-Cactus builds more complex graphs, capturing a wider range of genetic variations. While this detailed approach provides a richer genomic view, it also makes the read mapping process slower and more complex.

We tested the read mapping ability of both pangenomes and a linear reference in the case of real-world reads as well and the results showed the excellent ability of pangenomes to map real-world reads [32]. Minigraph-Cactus aligns 73.44% of total reads (Figure 9a) out of which, 82.77% of reads are aligned perfectly (Figure 9b). On the other hand, Minigraph aligns 73.27% of total reads (Figure 9a) out of which 78.13% are aligned perfectly (Figure 9b). While Minimap2 aligns 82.47% of total reads (Figure 9a) out of which only 68.77% are aligned perfectly (Figure 9b).

When comparing mapping ability of pangenomes to linear references, it is evident that pangenomes consistently offers superior alignment performance. Giraffe's alignment to both the Minigraph-Cactus and Minigraph pangenomes resulted in higher percentages of correctly mapped reads and total correct alignments compared to Minimap2's alignment to linear references.

We applied the same read simulation and mapping evaluation strategy to additional crop species, Soybean, Rapeseed and Barley, and their pangenome graphs constructed with Minigraph-Cactus and Minigraph. As observed in Sorghum, mapping with VG Giraffe to the pangenome graphs consistently outperformed alignment with Minimap2 to the linear reference genomes, both in terms of total mapped reads and correctly mapped reads across all species (Supplementary Table 1a). In addition to simulated reads, we also mapped publicly available real-world reads. Across all datasets, graph-based alignment consistently yielded a higher proportion of perfectly mapped reads than linear reference alignment. (Supplementary Table 1b).

Discussion

Previous benchmarking efforts focused mainly on animal genomes [42,51,57] which, compared to plants, can have very different properties including much higher levels of intra- and inter- species collinearity [58]. Assessing the methods' performance with crop data is therefore of high importance. This study offers a comparison of three key pangenome assembly pipelines (Minigraph-Cactus, PGGB and Minigraph) using sorghum as a model, highlighting how each approach handles the complexity of pangenome construction, content, and performance in variant calling and mapping. One of the key tasks of pangenome graph construction pipelines is an effective identification of regions of sequence correspondence and variation from whole genome alignments. We compared performance of the state-of-the-art pangenome pipelines (Minigraph-Cactus, PGGB, Minigraph) with established pipelines for SV

identification from pairwise genome alignments (SVIM-asm and SyRI). To thoroughly evaluate the performance of the SV calling pipelines, we used both simulated and real-world datasets. The simulated data, based on a reference backbone and containing known SVs, provided a controlled environment where we could assess variant calling accuracy with a known true set of SVs. In contrast, the real-world data, which includes inherent biological complexities such as sequencing errors and structural variation, allowed us to test the pipelines under more challenging and realistic conditions. This dual approach ensured a comprehensive evaluation of each pipeline's performance across both ideal and complex scenarios.

While the pairwise variant callers (SVIM-asm and SyRI) had slightly higher F1 scores in the simulations compared to the top performing pangenome graph construction pipeline (Minigraph-Cactus), overall, pangenome graph-based methods showed strong performance in simulations and good alignment with previously reported variation in real world datasets. We speculate that the performance of pairwise variant callers could be inflated in simulations due to over-simplified scenarios, without small or multi-allelelic variants. While the complexity of Minigraph-Cactus and PGGB generated graphs leads to substantial disparity in the number of variants detected by the two pipelines, they both capture a large number of variants which appear to have been missed by the pairwise variant callers in real-world scenarios. Pangenome graph approaches appear effective in identifying variants across multiple assemblies and capture both common and rare variants. Pangenome graph construction pipelines therefore emerge as a robust solution when dealing with complex, large datasets in a real-world scenario.

Graph-based pangenomes have been increasingly recognized as a superior approach compared to de novo or iterative methods, as they provide a unified framework to represent complex variation and multiple haplotypes simultaneously. Graph pangenomes have the capacity to improve variant discovery and read mapping accuracy by reducing reference bias and better capturing structural diversity [51][59]. For example, Lemay et al. [60] and Vaughn et al. [61] demonstrated that graph-based approaches enable accurate genotyping of variants across large populations. By capturing complex allelic variation and sequence context often missed by linear-reference-based methods, allowing for more comprehensive representation of population diversity. Our results align with these findings, reinforcing the value of graph-based approaches for comprehensive and scalable pangenome construction in diverse plant species.

To evaluate whether the trends observed in *Sorghum bicolor* are consistent across diverse plant genomes, we extended key analyses to three additional crops: *Glycine max* (soybean), *Brassica*

napus (canola), and *Hordeum vulgare* (barley). Using real-world assemblies, we applied the same pangenome construction and variant calling pipelines, focusing on graph sizes, completeness, duplication levels, variant detection. Pangenome graph sizes and duplication rates followed similar patterns across methods, and the low duplication levels observed in k-mer based analyses indicate good compressibility of the pangenomes despite genome complexity. These cross-species analyses highlight the general applicability and robustness of the pipelines for plant pangenome construction and variant discovery, underscoring their potential utility across crops with varying genome architectures.

The study revealed that pangenome-based approaches generally offer better alignment accuracy compared to traditional linear references for mapping performance. Giraffe, used with both Minigraph-Cactus and Minigraph pangenomes, consistently outperformed Minimap2 in correctly mapping reads, underscoring the advantages of graph-based methods for managing complex genomic data. However, PGGB's computational demands were a significant challenge, especially in generating mapping indexes, indicating the need for further optimization.

The identification of genetic variation through pangenome methods becomes particularly challenging when dealing with highly complex variations, such as nested or tandemly repeated transposable element insertions [62] Recent analysis in rice demonstrates that the Minigraph-Cactus pipeline can accurately identify five different alleles of a complex locus consisting of nested and tandemly repeated transposon insertions within a pangenome of 20 different accessions of Oryza sativa. Compared to a linear pangenome analysis, pangenome graph approach allowed for improved characterization of multiple related alleles. However, correct genotyping of multiple alleles in a large population using short reads remained challenging.

In this study, we constructed pangenomes for each chromosome separately, as this approach offers several advantages, including reduced processing time, simplified data management, and easier identification of variations within individual chromosomes. However, it does not account for the connections between chromosomes. To investigate complex chromosomal changes, such as Robertsonian translocations or centromeric homology [57], building a whole genome pangenome may be essential.

Our findings emphasize the importance of selecting tools aligned with specific research objectives, whether prioritizing variant discovery, optimizing computational resources, or

ensuring high mapping accuracy. PGGB is designed as a completely reference free approach relaying on all-vs-all alignments, however this appears to result in a potential trade-off in precision and recall of SV identification. Minigraph-Cactus progressively builds a graph and appears to offer a balanced integration of new sequences with robust performance. Minigraph, though efficient for identifying large structural variants, will miss finer variant details, particularly in highly diverse regions. Despite the advantages of pangenome-based approaches in capturing both common and rare variants across multiple assemblies, this study also highlights that these methods still require improvements. Additionally, refining pangenome assembly tools to address the unique complexities of plant genomes, will yield more accurate and comprehensive genomic analyses.

Acknowledgements

We thank Prof. Emma Mace (University of Queensland) for assistance with generation of simulated benchmarking dataset. We thank Dr. Cassandra Wattenburger (Bayer) for critical reading of the manuscript and helpful suggestions. This project was supported by the LOEWE Start Professorship from the Hessian Ministry of Higher Education, Research, Science and the Arts to AAG. VK was supported by GRK 2843 from the German Research Foundation (DFG). NM was supported by MCIU/AEI /10.13039/501100011033 and "ESF Investing in your future", Grant PRE2020-095111. KA was supported by Deutsche Forschungsgemeinschaft (German Research Foundation) project no. 497667402. This work was supported by the de.NBI Cloud within the German Network for Bioinformatics Infrastructure (de.NBI) and ELIXIR-DE (Forschungszentrum Jülich and W-de.NBI-001, W-de.NBI-004, W-de.NBI-008, W-de.NBI-010, W-de.NBI-013, W-de.NBI-014, W-de.NBI-016, W-de.NBI-022) and Justus Liebig University Bioinformatics Core Facility (BCF).

Competing interests

The authors declare no competing interests.

Author contributions

Venkataramana Kopalli: Formal analysis; Methodology; Writing – original draft; Writing – review & editing. **Kübra Arslan**: Methodology. **Noemia Morales-Díaz**: Writing – original draft; Writing – review & editing. **Silvia F. Zanini**: Writing – review & editing. **Agnieszka**

A. Golicz: Conceptualization; Funding acquisition; Methodology; Project administration; Supervision; Writing – original draft; Writing – review & editing.

Funding

This project was supported by the Hessian Ministry of Higher Education, Research, Science and the Arts, LOEWE Start Professorship, German Research Foundation (DFG), GRK 2843, German Research Foundation(DFG), project no. 497667402, and MCIU/AEI /10.13039/501100011033 and "ESF Investing in your future", Grant PRE2020-095111.

Data availability

The sorghum genome assemblies used in this study were re-used from the CNGB Nucleotide Sequence Archive project [63]. Other data further supporting this work are openly available in the *GigaScience* repository, GigaDB [64].

Availability of Source Code and Requirements

Project name: Benchmarking_graph_pipelines
Project homepage: https://github.com/KopalliV/Benchmarking_graph_pipelines
License: GPL-3.0 license
SciCrunch RRID: SCR_026567
bio.tools ID: benchmarking_graph_pipelines

System requirements
Operating system: Linux
Programming language: Bash, R and Python
Package management: Conda/bioconda, pip
Hardware requirements: HPC environment with ≥32 CPU cores, ≥128 GB RAM, and ~2 TB storage

References:

1. Wang J, Yang W, Zhang S, Hu H, Yuan Y, Dong J, et al.. A pangenome analysis pipeline provides insights into functional gene identification in rice. *Genome Biol*. 2023; doi: 10.1186/s13059-023-02861-9.

2. Li F, Hu H, Xiao Z, Wang J, Liu J, Zhao D, et al.. Visualization and review of reads alignment on the graphical pan-genome with VAG. *bioRxiv*. 2023;

3. Tay Fernandez CG, Nestor BJ, Danilevicz MF, Marsh JI, Petereit J, Bayer PE, et al.. Expanding Gene-Editing Potential in Crop Improvement with Pangenomes. Int J Mol Sci.

4. Outten J, Warren A. Methods and Developments in Graphical Pangenomics. J Indian Inst Sci.

5. Zia K, Rao MJ, Sadaqat M, Azeem F, Fatima K, Tahir ul Qamar M, et al.. Pangenome-wide analysis of cyclic nucleotide-gated channel (CNGC) gene family in citrus Spp. Revealed their

intraspecies diversity and potential roles in abiotic stress tolerance. *Front Genet*. 2022; doi: 10.3389/fgene.2022.1034921.

6. Zanini SF, Bayer PE, Wells R, Snowdon RJ, Batley J, Varshney RK, et al.. Pangenomics in crop improvement—from coding structural variations to finding regulatory variants with pangenome graphs. Plant Genome.

7. Wang W, Mauleon R, Hu Z, Chebotarov D, Tai S, Wu Z, et al.. Genomic variation in 3,010 diverse accessions of Asian cultivated rice. *Nature*. 2018; doi: 10.1038/s41586-018-0063-9.

8. Hirsch CN, Foerster JM, Johnson JM, Sekhon RS, Muttoni G, Vaillancourt B, et al.. Insights into the maize pan-genome and pan-transcriptome. *Plant Cell*. 2014; doi: 10.1105/tpc.113.119982.

9. Song JM, Guan Z, Hu J, Guo C, Yang Z, Wang S, et al.. Eight high-quality genomes reveal pan-genome architecture and ecotype differentiation of Brassica napus. *Nat Plants*. 2020; doi: 10.1038/s41477-019-0577-7.

10. Paterson AH, Bowers JE, Bruggmann R, Dubchak I, Grimwood J, Gundlach H, et al.. The Sorghum bicolor genome and the diversification of grasses. *Nature*. 2009; doi: 10.1038/nature07723.

11. Zhang LM, Luo H, Liu ZQ, Zhao Y, Luo JC, Hao DY, et al.. Genome-wide patterns of large-size presence/absence variants in sorghum. *J Integr Plant Biol*. 2014; doi: 10.1111/jipb.12121.

12. Wang B, Jiao Y, Chougule K, Olson A, Huang J, Llaca V, et al.. Pan-genome analysis in sorghum highlights the extent of genomic variation and sugarcane aphid resistance genes. bioRxiv.

13. Satish L, Shilpha J, Pandian S, Rency AS, Rathinapriya P, Ceasar SA, et al.. Analysis of genetic variation in sorghum (Sorghum bicolor (L.) Moench) genotypes with various agronomical traits using SPAR methods. *Gene*. 2016; doi: 10.1016/j.gene.2015.10.056.

14. Li H, Feng X, Chu C. The design and construction of reference pangenome graphs with minigraph. *Genome Biol*. 2020; doi: 10.1186/s13059-020-02168-z.

15. Bayer PE, Petereit J, Durant É, Monat C, Rouard M, Hu H, et al.. Wheat Panache: A pangenome graph database representing presence–absence variation across sixteen bread wheat genomes. *Plant Genome*. 2022; doi: 10.1002/tpg2.20221.

16. Garrison E, Guarracino A, Heumos S, Villani F, Bao Z, Tattini L, et al.. Building pangenome graphs. *bioRxiv*. 2023;

17. Yang Z, Guarracino A, Biggs PJ, Black MA, Ismail N, Wold JR, et al.. Pangenome graphs in infectious disease: a comprehensive genetic variation analysis of Neisseria meningitidis leveraging Oxford Nanopore long reads. *Front Genet*. 2023; doi: 10.3389/fgene.2023.1225248.

18. Hickey G, Monlong J, Ebler J, Novak AM, Eizenga JM, Gao Y, et al.. Pangenome graph construction from genome alignments with Minigraph-Cactus. *Nat Biotechnol*. 2023; doi: 10.1038/s41587-023-01793-w.

19. Skiadas P, Riera Vidal S, Dommisse J, Mendel MN, Elberse J, Van den Ackerveken G, et al.. Pangenome graph analysis reveals extensive effector copy-number variation in spinach downy mildew. Barber AE, editor. *PLoS Genet*. 2024; doi: 10.1371/journal.pgen.1011452.

20. Golicz AA, Bayer PE, Barker GC, Edger PP, Kim HR, Martinez PA, et al.. The pangenome of an agronomically important crop plant Brassica oleracea. *Nat Commun*. 2016; doi: 10.1038/ncomms13390.

21. Eizenga JM, Novak AM, Sibbesen JA, Heumos S, Ghaffaari A, Hickey G, et al.. Pangenome Graphs. *Annu Rev Genomics Hum Genet*. Annual Reviews Inc.; 2020; doi: 10.1146/ANNUREV-GENOM-120219-080406/CITE/REFWORKS.

22. MacNish TR, Al-Mamun HA, Bayer PE, McPhan C, Fernandez CGT, Upadhyaya SR, et al.. Brassica Panache: A multi-species graph pangenome representing presence absence variation across forty-one Brassica genomes. *Plant Genome*. John Wiley and Sons Inc; 2025; doi: 10.1002/tpg2.20535.

23. Zhou Y, Zhang Z, Bao Z, Li H, Lyu Y, Zan Y, et al.. Graph pangenome captures missing heritability and empowers tomato breeding. *Nature*. 2022; doi: 10.1038/s41586-022-04808-9.

24. Liu Y, Du H, Li P, Shen Y, Peng H, Liu S, et al.. Pan-Genome of Wild and Cultivated Soybeans. *Cell*. 2020; doi: 10.1016/j.cell.2020.05.023.

25. Liu Z, Wang N, Su Y, Long Q, Peng Y, Shangguan L, et al.. Grapevine pangenome facilitates trait genetics and genomic breeding. *Nat Genet*. Nature Research; 2024; doi: 10.1038/s41588-024-01967-5.

26. Danilevicz MF, Tay Fernandez CG, Marsh JI, Bayer PE, Edwards D. Plant pangenomics: approaches, applications and advancements. Curr Opin Plant Biol.

27. Golicz AA, Batley J, Edwards D. Towards plant pangenomics. Plant Biotechnol J.

28. Heller D, Vingron M. SVIM-asm: Structural variant detection from haploid and diploid genome assemblies. *Bioinformatics*. 2020; doi: 10.1093/bioinformatics/btaa1034.

29. Goel M, Sun H, Jiao WB, Schneeberger K. SyRI: finding genomic rearrangements and local sequence differences from whole-genome assemblies. *Genome Biol*. 2019; doi: 10.1186/s13059-019-1911-0.

30. Song B, Buckler ES, Stitzer MC. New whole-genome alignment tools are needed for tapping into plant diversity. Trends Plant Sci.

31. Yildiz G, Zanini SF, Afsharyan NP, Obermeier C, Snowdon RJ, Golicz AA. Benchmarking Oxford Nanopore read alignment-based insertion and deletion detection in crop plant genomes. *Plant Genome*. 2023; doi: 10.1002/tpg2.20314.

32. Tao Y, Luo H, Xu J, Cruickshank A, Zhao X, Teng F, et al.. Extensive variation within the pan-genome of cultivated and wild sorghum. *Nat Plants*. 2021; doi: 10.1038/s41477-021-00925-x.

33. Jayakodi M, Lu Q, Pidon H, Rabanus-Wallace MT, Bayer M, Lux T, et al.. Adaptive diversification through structural variation in barley. *bioRxiv*. 2024;

34. Rousseau-Gueutin M, Belser C, Silva C Da, Richard G, Istace B, Cruaud C, et al.. Long-read assembly of the Brassica napus reference genome Darmor-bzh. *Gigascience*. 2021; doi: 10.1093/gigascience/giaa137.

35. Mascher M, Wicker T, Jenkins J, Plott C, Lux T, Koh CS, et al.. Long-read sequence assembly: A technical evaluation in barley. *Plant Cell*. 2021; doi: 10.1093/plcell/koab077.

36. Valliyodan B, Cannon SB, Bayer PE, Shu S, Brown A V., Ren L, et al.. Construction and comparison of three reference-quality genome assemblies for soybean. *Plant Journal*. 2019; doi: 10.1111/tpj.14500.

37. Bolognini D, Sanders A, Korbel JO, Magi A, Benes V, Rausch T. VISOR: A versatile haplotype-aware structural variant simulator for short-and long-read sequencing. *Bioinformatics*. 2020; doi: 10.1093/bioinformatics/btz719.

38. Danecek P, Bonfield JK, Liddle J, Marshall J, Ohan V, Pollard MO, et al.. Twelve years of SAMtools and BCFtools. *Gigascience*. 2021; doi: 10.1093/gigascience/giab008.

39. Manni M, Berkeley MR, Seppey M, Zdobnov EM. BUSCO: Assessing Genomic Data Quality and Beyond. *Curr Protoc*. 2021; doi: 10.1002/cpz1.323.

40. Guarracino A, Heumos S, Nahnsen S, Prins P, Garrison E. ODGI: Understanding pangenome graphs. *Bioinformatics*. 2022; doi: 10.1093/bioinformatics/btac308.

41. Parmigiani L, Garrison E, Stoye J, Marschall T, Doerr D. Panacus: fast and exact pangenome growth and core size estimation. *bioRxiv*. 2024; doi: 10.1101/2024.06.11.598418.

42. Liao WW, Asri M, Ebler J, Doerr D, Haukness M, Hickey G, et al.. A draft human pangenome reference. *Nature*. 2023; doi: 10.1038/s41586-023-05896-x.

43. Li H. Minimap2: Pairwise alignment for nucleotide sequences. *Bioinformatics*. 2018; doi: 10.1093/bioinformatics/bty191.

44. Kirsche M, Prabhu G, Sherman R, Ni B, Battle A, Aganezov S, et al.. Jasmine and Iris: population-scale structural variant comparison and analysis. *Nat Methods*. 2023; doi: 10.1038/s41592-022-01753-3.

45. English AC, Menon VK, Gibbs RA, Metcalf GA, Sedlazeck FJ. Truvari: refined structural variant comparison preserves allelic diversity. *Genome Biol*. 2022; doi: 10.1186/s13059-022-02840-6.

46. Gel B, Díez-Villanueva A, Serra E, Buschbeck M, Peinado MA, Malinverni R. RegioneR: An R/Bioconductor package for the association analysis of genomic regions based on permutation tests. *Bioinformatics*. 2016; doi: 10.1093/bioinformatics/btv562.

47. De Coster W, De Rijk P, De Roeck A, De Pooter T, D'Hert S, Strazisar M, et al.. Structural variants identified by Oxford Nanopore PromethION sequencing of the human genome. *Genome Res*. 2019; doi: 10.1101/gr.244939.118.

48. Ou S, Su W, Liao Y, Chougule K, Agda JRA, Hellinga AJ, et al.. Benchmarking transposable element annotation methods for creation of a streamlined, comprehensive pipeline. *Genome Biol*. 2019; doi: 10.1186/s13059-019-1905-y.

49. Camacho C, Coulouris G, Avagyan V, Ma N, Papadopoulos J, Bealer K, et al.. BLAST+: Architecture and applications. *BMC Bioinformatics*. 2009; doi: 10.1186/1471-2105-10-421.

50. Shumate A, Salzberg SL. Liftoff: Accurate mapping of gene annotations. *Bioinformatics*. 2021; doi: 10.1093/bioinformatics/btaa1016.

51. Rice ES, Alberdi A, Alfieri J, Athrey G, Balacco JR, Bardou P, et al.. A pangenome graph reference of 30 chicken genomes allows genotyping of large and complex structural variants. *BMC Biol*. 2023; doi: 10.1186/s12915-023-01758-0.

52. Sirén J, Monlong J, Chang X, Novak AM, Eizenga JM, Markello C, et al.. Pangenomics enables genotyping of known structural variants in 5202 diverse genomes. *Science (1979)*. 2021; doi: 10.1126/science.abg8871.

53. Garrison E, Sirén J, Novak AM, Hickey G, Eizenga JM, Dawson ET, et al.. Variation graph toolkit improves read mapping by representing genetic variation in the reference. *Nat Biotechnol*. 2018; doi: 10.1038/nbt.4227.

54. Li H. [Heng Li - Compares BWA to other long read aligners like CUSHAW2] Aligning sequence reads, clone sequences and assembly contigs with BWA-MEM. *arXiv preprint arXiv*. 2013;

55. : Simulating with a small sample. Issue #118 · lh3/minigraph · GitHub. https://github.com/lh3/minigraph/issues/118 Accessed 2024 Dec 4.

56. : Minigraph does not find variants in simulated data· Issue #119 · lh3/minigraph · GitHub. https://github.com/lh3/minigraph/issues/119 Accessed 2024 Dec 4.

57. Leonard AS, Crysnanto D, Mapel XM, Bhati M, Pausch H. Graph construction method impacts variation representation and analyses in a bovine super-pangenome. *Genome Biol*. 2023; doi: 10.1186/s13059-023-02969-y.

58. Zhao T, Eric Schranz M. Network-based microsynteny analysis identifies major differences and genomic outliers in mammalian and angiosperm genomes. *Proc Natl Acad Sci U S A*. 2019; doi: 10.1073/pnas.1801757116.

59. Yildiz G, Zanini SF, Weber S, Kopalli V, Kox T, Abbadi A, et al.. Graphical pangenomics-enabled characterization of structural variant impact on gene expression in Brassica napus. *Theoretical and Applied Genetics*. 2025; doi: 10.1007/s00122-025-04867-2.

60. Vaughn JN, Branham SE, Abernathy B, Hulse-Kemp AM, Rivers AR, Levi A, et al.. Graph-based pangenomics maximizes genotyping density and reveals structural impacts on fungal resistance in melon. *Nat Commun*. 2022; doi: 10.1038/s41467-022-35621-7.

61. Lemay MA, Sibbesen JA, Torkamaneh D, Hamel J, Levesque RC, Belzile F. Combined use of Oxford Nanopore and Illumina sequencing yields insights into soybean structural variation biology. *BMC Biol*. 2022; doi: 10.1186/s12915-022-01255-w.

62. Morales-Díaz N, Sushko S, Campos-Domínguez L, Kopalli V, Golicz A, Castanera R, et al.. Tandem LTR-retrotransposon structures are common and highly polymorphic in plant genomes. 2024; doi: 10.21203/RS.3.RS-5356060/V1.

63. Teng F. pan-genome of cultivated and wild sorghum. CNGBdb. 2021; DOI: 10.26036/CNP0001440.

64. Kopalli V; Arslan K; Morales-Díaz N; Zanini SF; Golicz AA. Supporting data for "Towards a Standardized Framework for Pangenome Graph Evaluation: Assessing Crop Plant Pangenome Variation Graph Construction from Multiple Assemblies" GigaScience Database. 2025; https://doi.org/10.5524/102758

Figures

Figure 1: This flowchart illustrates the workflow used in our study to evaluate pangenome graph-building pipelines. Public sorghum data was used to generate simulated assemblies, which, along with real-world assemblies, were analyzed using pangenome graph-building pipelines and pairwise comparison tools. The resulting outputs were then assessed to evaluate their performance and accuracy.

Figure 2: Comparison of the number of nodes and edges for Minigraph-Cactus, PGGB, and Minigraph pangenome graphs constructed from simulated assemblies.

Figure 3: Benchmarking structural variant detection across tools using simulated data. (a) F1 score comparison for the non-redundant merged variant set across all assemblies shows SVIM-asm achieving the best performance (0.93), followed closely by SyRI (0.91) and Minigraph-Cactus (0.89), while PGGB has the lowest score (0.83). (b) Per-sample F1 score distribution reveals consistent performance trends across samples for all methods, with SVIM-asm and SyRI generally outperforming graph-based approaches. (c) UpSet plot showing overlap in SV calls among SyRI, SVIM-asm, PGGB, and Minigraph-Cactus with the ground truth. The largest shared intersection is among all four tools.

Figure 4. Comparison of key graph characteristics and gene representation across pangenome graphs constructed using Minigraph-Cactus, PGGB, and Minigraph for real-world sorghum assemblies. (a) Number of missing genes across assemblies in each pangenome graph. Minigraph consistently shows the highest number of missing genes, indicating reduced gene representation compared to PGGB and Minigraph-Cactus. (b) Comparison of the number of nodes and edges in the pangenome graphs. PGGB and Minigraph-Cactus have substantially more nodes and edges than Minigraph, reflecting finer-scale graph resolution. (c) Coverage of each input assembly within the pangenome graph. Light blue indicates the portion of the assembly included in the graph, while light green shows the excluded portion.

Figure 5: Comparison of a) Graph file sizes and number of nodes and edges for Minigraph-Cactus, PGGB, and Minigraph pangenome graphs constructed from b) Soybean, c) Barley, d) Rapeseed.

Figure 6: Pie charts comparing the proportion of different variant types called by Minigraph-Cactus, PGGB, Minigraph, SVIM-asm, and SyRI. Minigraph and SVIM-asm focus on structural variants and do not call SNPs or MNPs. The “Others" category includes complex variant types like duplications, inversions, and other structural rearrangements.

Figure 7: Overlap of variants in real-world assemblies. a) An UpSet plot showing the overlap of large variants (>50bp) between different methods, the bars represent the number of shared variants between dataset combinations, with the matrix below indicating which datasets are included. b) An UpSet plot showing the overlap of small variants (<50bp). c) Number of Multiallelic sites detected by each of the three pipelines.

Figure 8: Comparison of SVs with similarity to repeats for Minigraph-Cactus and PGGB; a) Total counts of structural variations (All SVs) and SVs with similarity to repeats detected by each method, including the count of SVs with similarity to repeats after applying 80% coverage

filter b) Count of unique variants identified by Minigraph-Cactus and PGGB and the count of SV with similarity to repeats in those unique variants.

Figure9: Comparison of read alignment metrics for Minigraph-Cactus, Minigraph and Minimap2 for real-world reads. (a) displays the percentage of total reads successfully aligned by each tool: Minimap2 achieved the highest alignment rate at 82.47%, followed by Minigraph-Cactus with 73.44% and Minigraph with 73.27%. (b) shows the percentage of perfectly aligned reads, where Minigraph-Cactus outperformed the others with an alignment rate of 82.77%, Minigraph had 78.13% and Minimap2 only achieved 68.77%, highlighting the lower quality of read alignments to the linear reference (Minimap2) in (a) when compared to pangenome graph references (Minigraph-Cactus and Minigraph).

Figure
Click here to access/download;Figure;figs_revised_22092025_highres.pdf

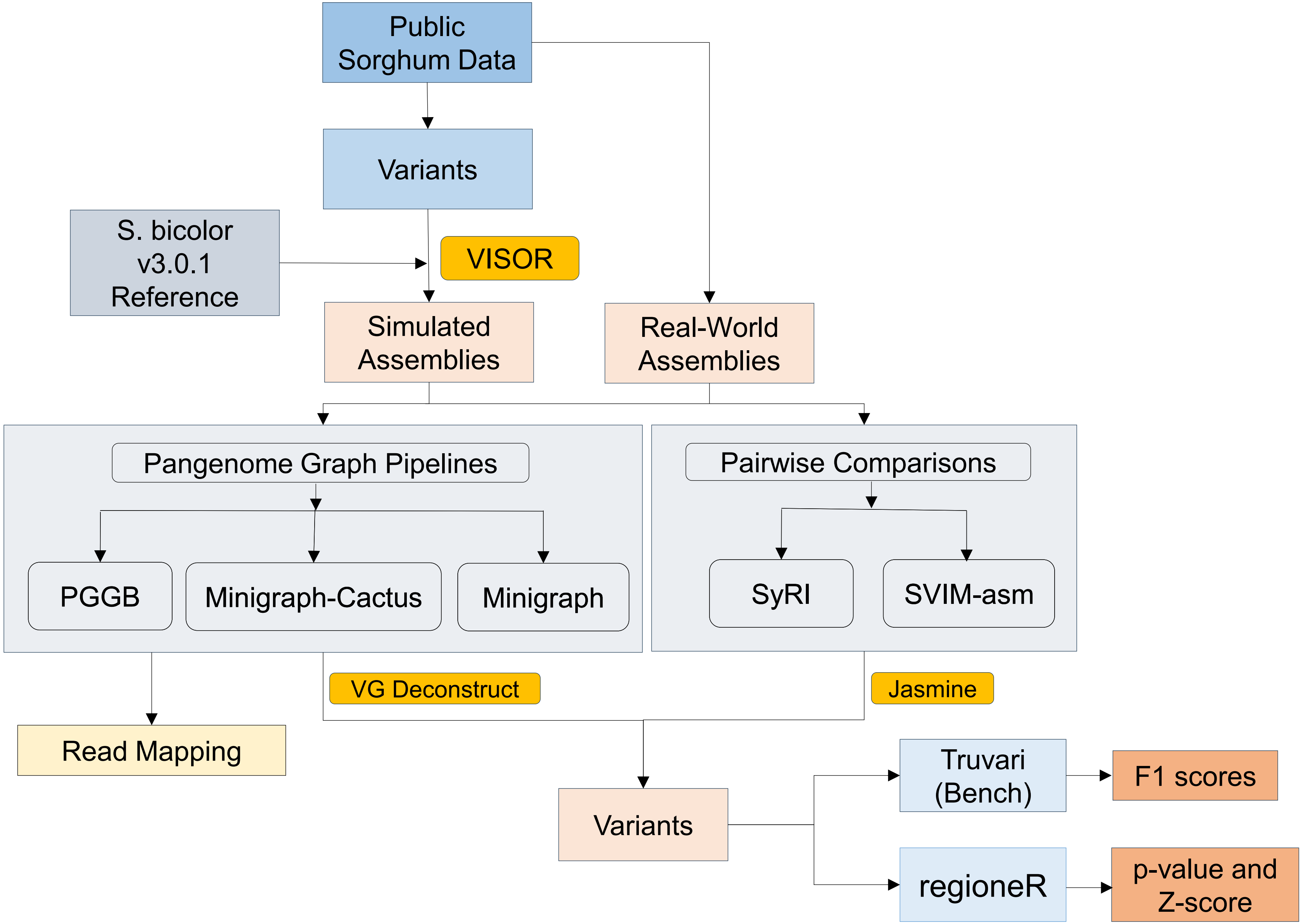

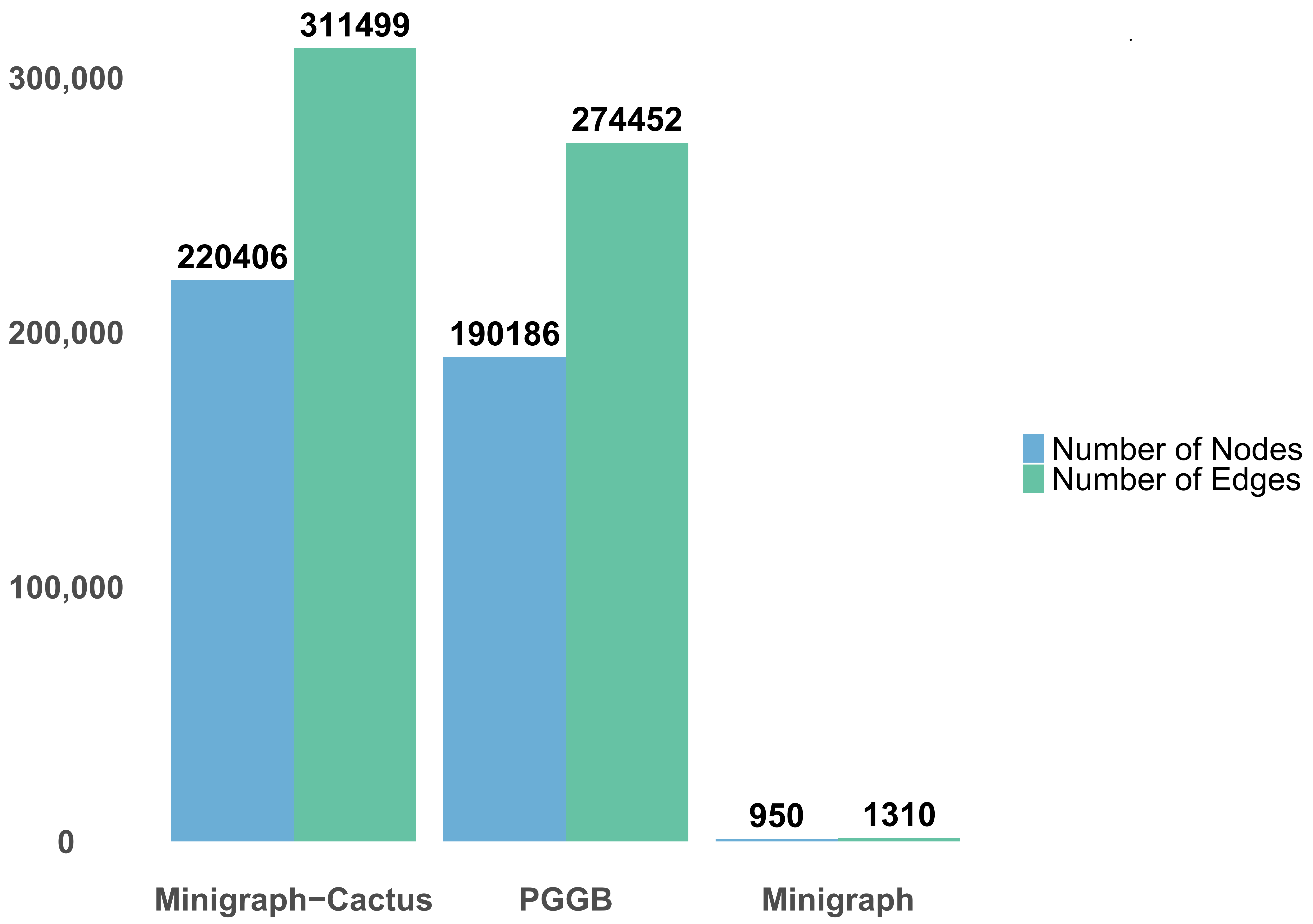
311499
300,000
274452
220406
200,000
190186
Number of Nodes
Number of Edges
100,000
950
1310
0
Minigraph−Cactus
PGGB
Minigraph

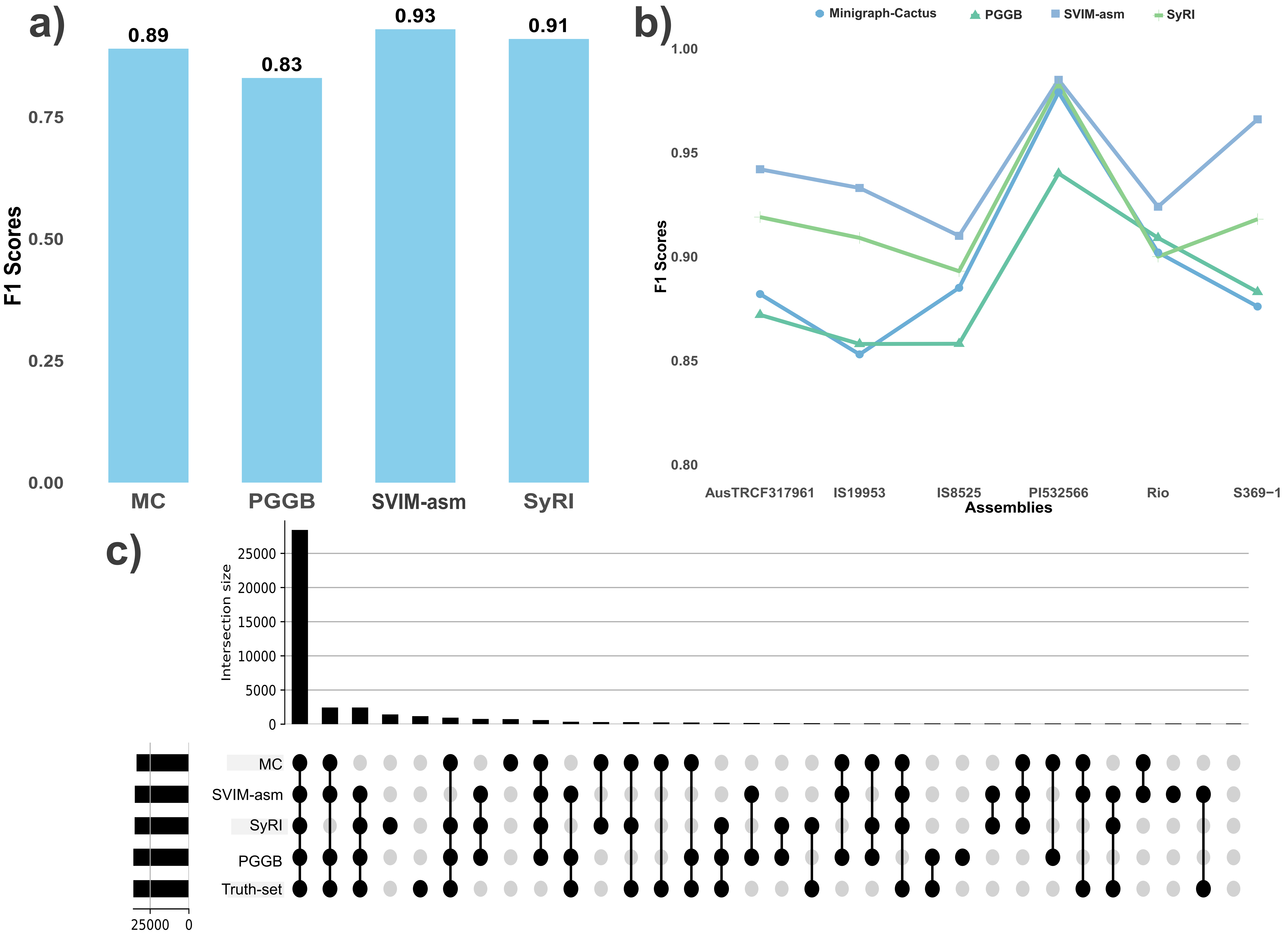
a)
0.89
0.83
0.93
0.91
F1 Scores
0.00
0.25
0.50
0.75
MC
PGGB
SVIM-asm
SyRI
b)
Minigraph-Cactus
PGGB
SVIM-asm
SyRI
F1 Scores
0.80
0.85
0.90
0.95
1.00
AusTRCF317961
IS19953
IS8525
PI532566
Rio
S369−1
Assemblies
c)
Intersection size
0
5000
10000
15000
20000
25000
MC
SVIM-asm
SyRI
PGGB
Truth-set
25000
0

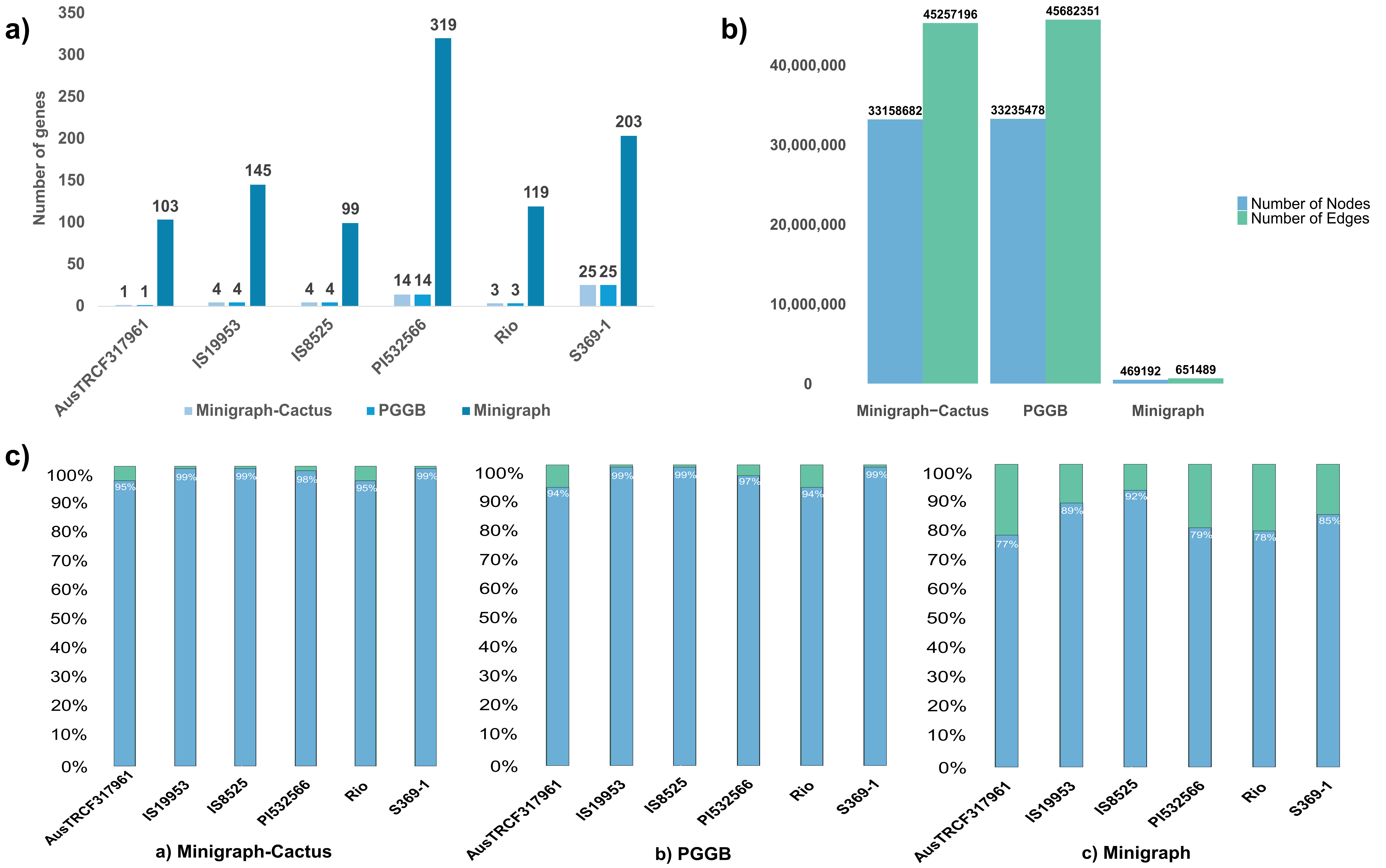

a)
Number of genes
350
300
250
200
150
100
50
0
1 1 103
4 4 145
4 4 99
14 14 319
3 3 119
25 25 203
AusTRCF317961
IS19953
IS8525
PI532566
Rio
S369-1
Minigraph-Cactus
PGGB
Minigraph
b)
40,000,000
30,000,000
20,000,000
10,000,000
0
33158682
45257196
33235478
45682351
469192
651489
Minigraph−Cactus
PGGB
Minigraph
Number of Nodes
Number of Edges
c)
100%
90%
80%
70%
60%
50%
40%
30%
20%
10%
0%
95%
99%
99%
98%
95%
99%
a) Minigraph-Cactus
94%
99%
99%
97%
94%
99%
b) PGGB
77%
89%
92%
79%
78%
85%
c) Minigraph

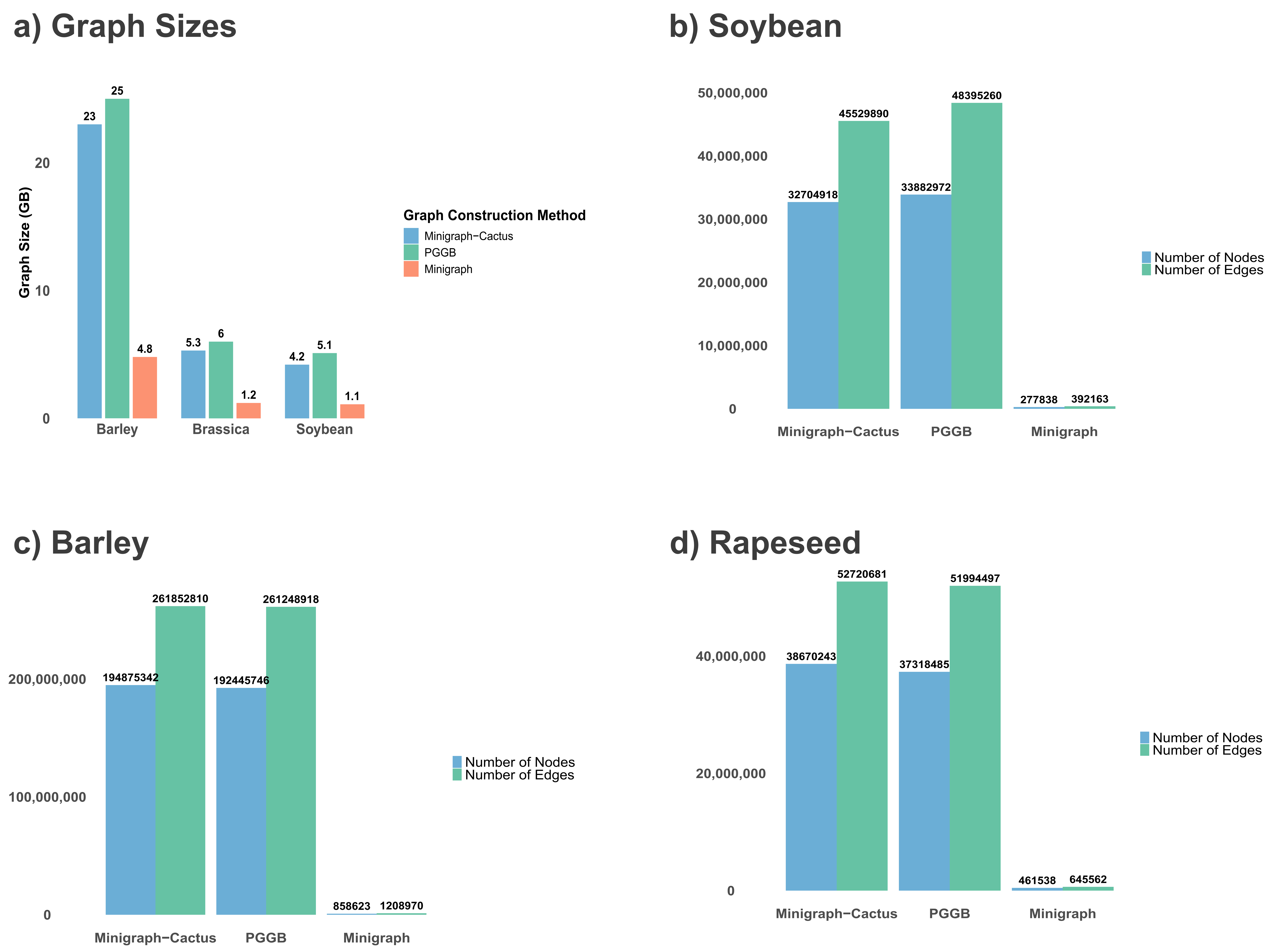

a) Graph Sizes
Graph Size (GB)
0
10
20
Barley
Brassica
Soybean
23
25
4.8
5.3
6
1.2
4.2
5.1
1.1
Graph Construction Method
Minigraph−Cactus
PGGB
Minigraph
b) Soybean
0
10,000,000
20,000,000
30,000,000
40,000,000
50,000,000
32704918
45529890
33882972
48395260
277838
392163
Minigraph−Cactus
PGGB
Minigraph
Number of Nodes
Number of Edges
c) Barley
0
100,000,000
200,000,000
194875342
261852810
192445746
261248918
858623
1208970
Minigraph−Cactus
PGGB
Minigraph
Number of Nodes
Number of Edges
d) Rapeseed
0
20,000,000
40,000,000
38670243
52720681
37318485
51994497
461538
645562
Minigraph−Cactus
PGGB
Minigraph
Number of Nodes
Number of Edges

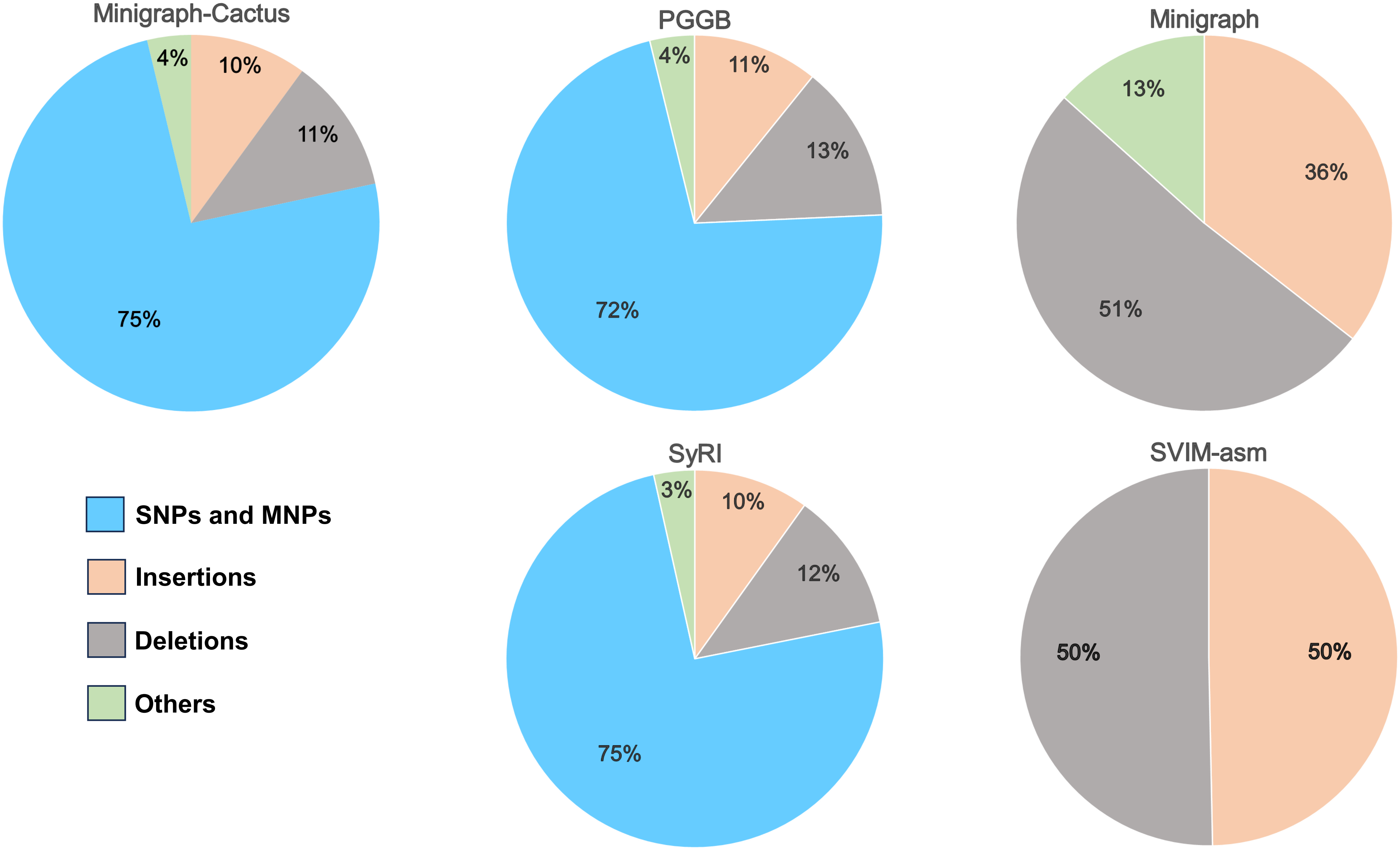
Minigraph-Cactus
4%
10%
11%
75%
PGGB
4%
11%
13%
72%
Minigraph
13%
36%
51%
SyRI
3%
10%
12%
75%
SVIM-asm
50%
50%
SNPs and MNPs
Insertions
Deletions
Others

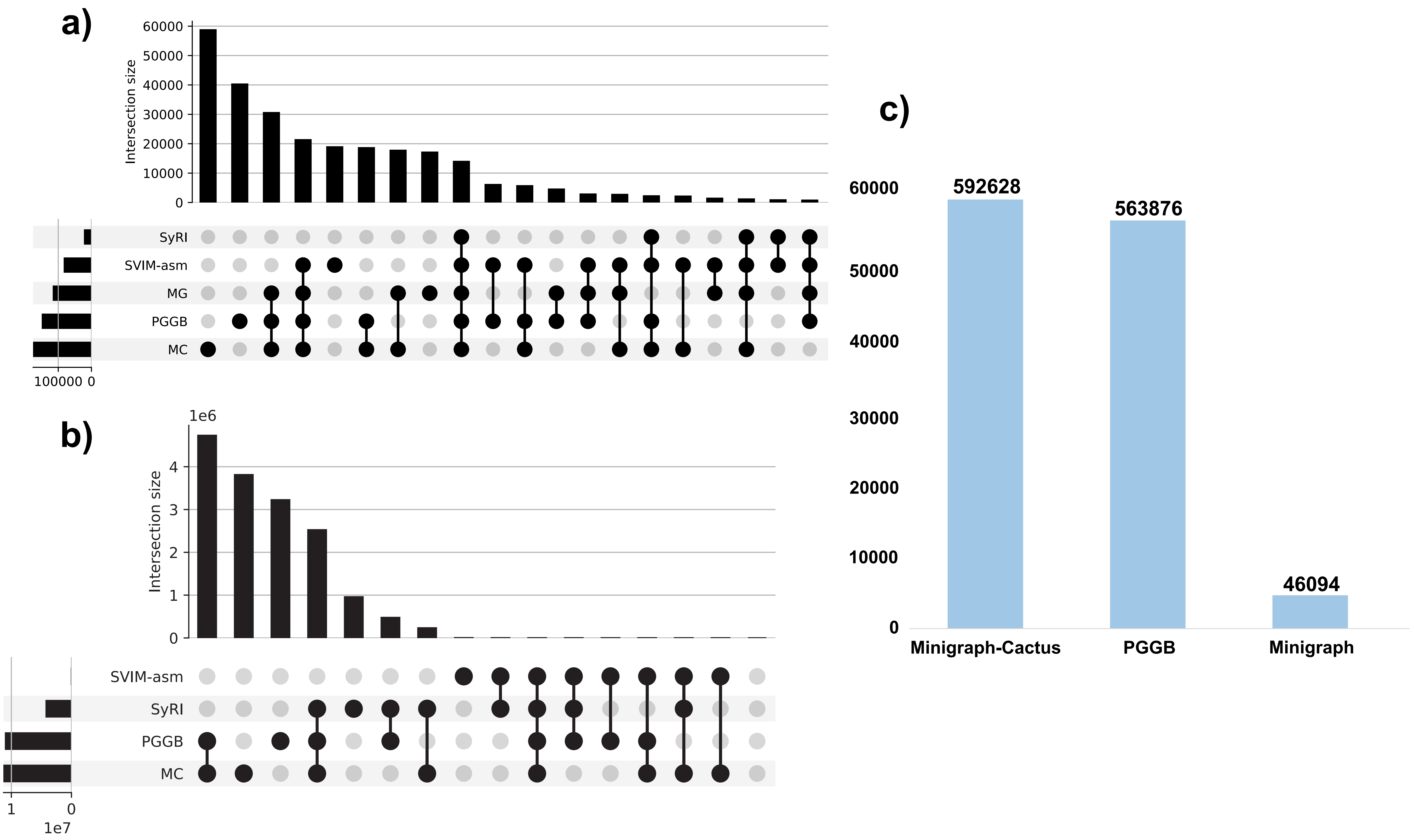

a)
Intersection size
60000
50000
40000
30000
20000
10000
0
SyRI
SVIM-asm
MG
PGGB
MC
100000 0
b)
1e6
Intersection size
4
3
2
1
0
SVIM-asm
SyRI
PGGB
MC
1 0
1e7
c)
60000
50000
40000
30000
20000
10000
0
592628
563876
46094
Minigraph-Cactus
PGGB
Minigraph

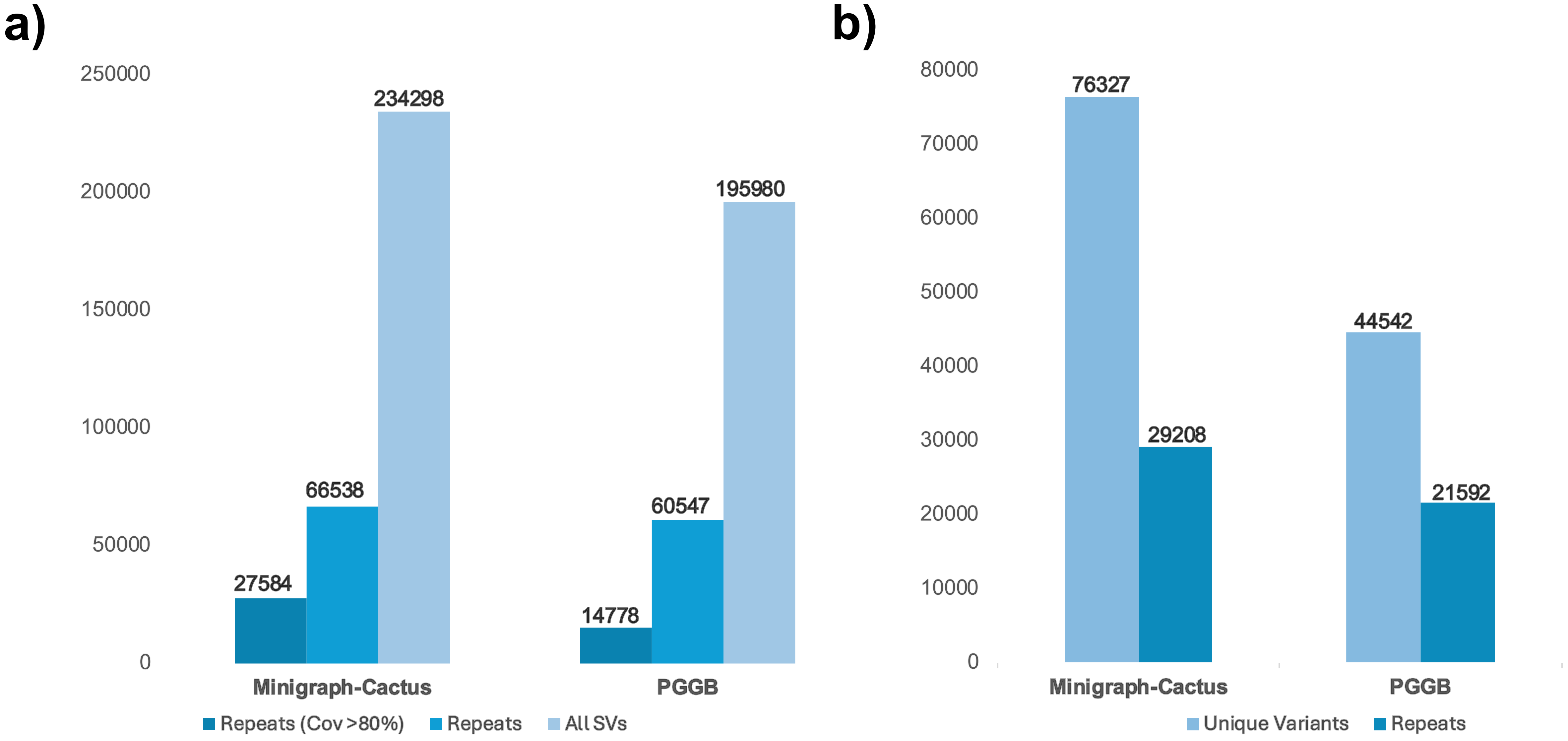

a)
250000
200000
150000
100000
50000
0
234298
66538
27584
195980
60547
14778
Minigraph-Cactus
PGGB
Repeats (Cov >80%)
Repeats
All SVs
b)
80000
70000
60000
50000
40000
30000
20000
10000
0
76327
29208
44542
21592
Minigraph-Cactus
PGGB
Unique Variants
Repeats

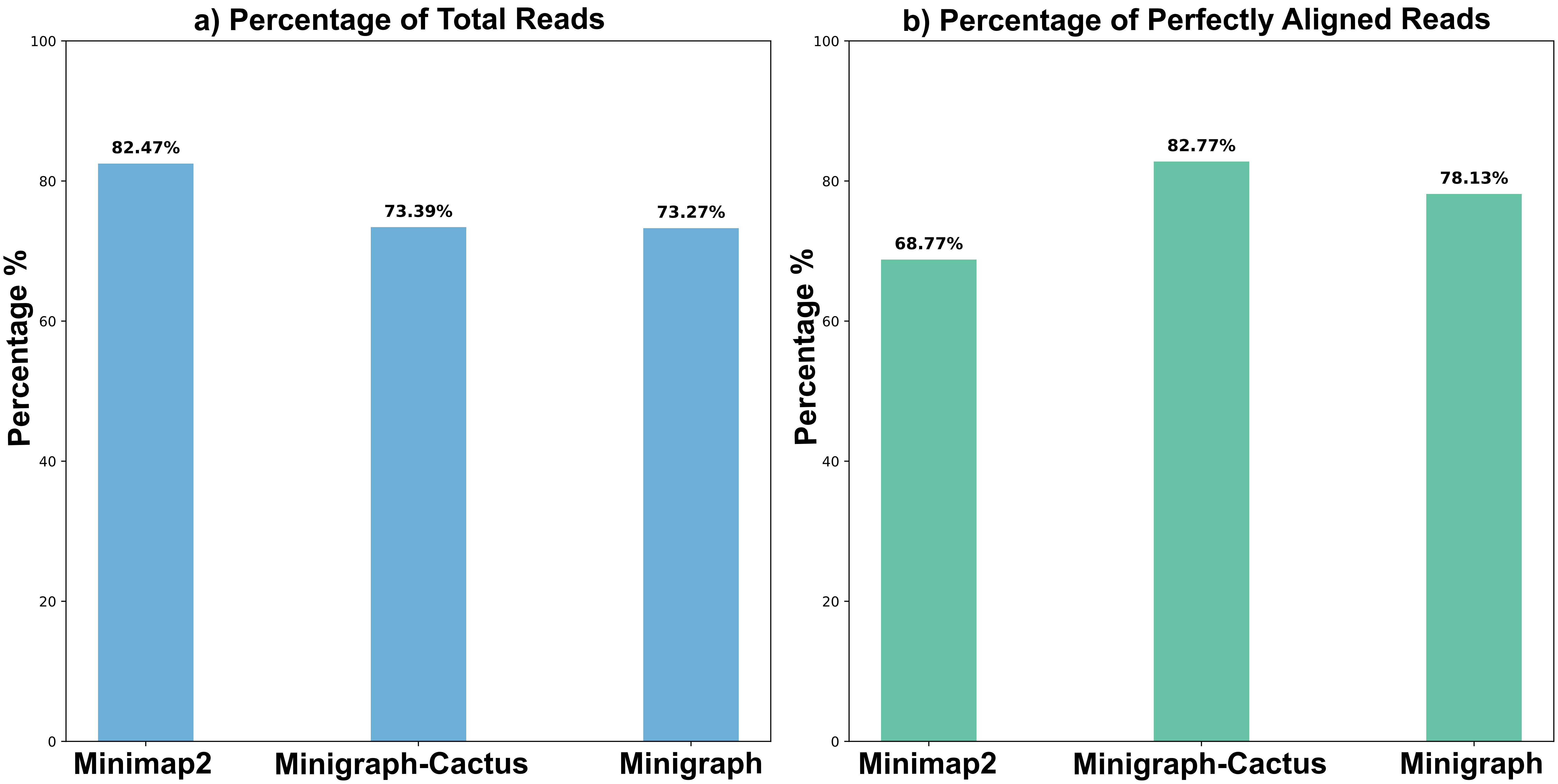
a) Percentage of Total Reads
Percentage %
82.47%
73.39%
73.27%
Minimap2
Minigraph-Cactus
Minigraph
b) Percentage of Perfectly Aligned Reads
Percentage %
68.77%
82.77%
78.13%
Minimap2
Minigraph-Cactus
Minigraph

Click here to access/download
Supplementary Material
Supplementary_data_08082025.docx

Click here to access/download
Supplementary Material
supplementary_tables_08082025.xlsx

Click here to access/download
Supplementary Material
Reviewer reports_answers_08082025.docx

Personal Cover Click here to access/download;Personal Cover;Coverletter_GigaScience_08.08.2025.docx

To

The Editor

08.08.2025

Dear Editor,

Please find attached the revised version of the manuscript of Kopalli et al. titled **"Towards a Standardized Framework for Pangenome Graph Evaluation: Assessing Sorghum Pangenome Variation Graph Construction from Multiple Assemblies"** for consideration for publication in *GigaScience*.

We hope that the revised version of our manuscript will be acceptable for publication in *GigaScience.* Please find detailed responses to reviewer comments below.

Thank you for considering our submission.

Best regards,

Prof. Dr. Agnieszka A. Golicz

Sofja Kovalevskaja Award Group Leader

Department of Agrobioinformatics

Justus Liebig University Gießen

IFZ Research Centre for Biosystems, Land Use and Nutrition

Heinrich-Buff-Ring 26-32, 35392 Gießen, Germany

Agnieszka.Golicz@agrar.uni-giessen.de

Reviewer reports:

Reviewer #1: The authors provide a good summary of pan genome Graph evaluations based on Sorghum pan genome assemblies. They provide details insights into strengths and limitation for 3 pan-genome graph construction tools Mingraph, PGGN and Monograph-Cactus. They propose metrics to evaluate comprehensive pan genome graph evaluation, which could be applied or standardized of other pan genome graph construction methods.

Major comments:

* Introduction; the authors although highlight the importance of Pan-genomes and what value they offer. Could the authors also comment on why pan genome variation graph are important; specifically what advantages they offer over traditional linear based pan genomes.

Ans: Thank you for your feedback, we have now added a paragraph (Lines 84-94) in the Introduction highlighting the importance of variation graphs.

* Methods:

- Genome assembly and chromosome splitting-

- Although the authors have chosen accession that are diverse. Could they comment also on the quality of the assembly. For building graph-based pan genomes having high quality assemblies are crucial to avoid artificial bubbles or paths in the graphs that are built with the tools used for benchmarking. How much does the quality of assembly inflate the complexity of the pan-genome graphs built. Some background of the quality of the assembly would help in here to understand the benchmarking metrics that authors are testing

Ans: All selected genomes were assembled using both Illumina short reads and PacBio long reads, resulting in high-quality assemblies with contig N50 values up to 3.5 Mb and BUSCO completeness scores exceeded 95%, indicating strong representation of the gene space. (Added in lines 123-124)

- Simulated Genomes Assemblies-

- Can you clarify if the SV's used with VISOR for generating simulated assemblies are real or are the SV's provided simulated too?

Thank you, we have now clarified in the text that the SV’s used with VISOR are real SVs. (Line 135)

* Read Mapping

- Line 404 : Generating mapping indexes with PGGB pan genome graph was mentioned to be difficult due to the graph complexity and heavy computational demand. Could authors try read mapping with smaller subset graph and generate index for the smaller graph. Maybe reduce the graph to chr level?

Ans: Thank you for the suggestion. We revisited the task of generating mapping indexes for the PGGB pangenome graph. We were able to successfully build whole-genome mapping indexes, but the process required approximately 2.2 TB of memory. The resulting index files were extremely large, and attempting to map reads back to them would be computationally expensive in most practical scenarios.

We also tested building indexes at the chromosome level, which was feasible with much lower resource requirements. However, the utility of individually indexed chromosomes for genome-wide analyses is limited (https://github.com/vgteam/vg/issues/3841).

- Line 417: what is correctly mapped reads? min_mapQ > 60 ?

Ans: Correctly mapped reads are simulated reads that align back to their original genomic position from where they were simulated from. 'min_mapQ 60' stands for a minimum mapping quality score of 60. This has now been clarified in the manuscript.

- Fig12: It was really hard to read and follow the figure and text in the main section. Suggest Figure 12 split as a,b,c, & d and refer them in the main text. Maybe also uses lines in the figure to show boundaries.

Ans: Thank you for the suggestion. We revisited Figure 12 and agree that the original layout was difficult to follow. To improve clarity and readability, we replaced it with a table (now Supplementary Table 1), which presents the data in a more accessible and organized format.

Minor comments

* Line 294: It should be mentioned as Figure 3 instead of Figure 2

Ans: Thank you, this has now been corrected.

* Suggest combining Fig 3, 4, & 5 as a facet plot for better readability and comparison

Ans: We combined Figures 3, 4, and 5 into a single facet plot, this updated figure is now presented as Figure 3

* Suggest combining Fig 6, 7, & 8 as a facet plot for better readability and comparison

Ans: We combined Figures 6, 7, and 8 into a single facet plot, this updated figure is now presented as Figure 4

* Figure 9 : Suggest using different choice for legends. Hard to distinguish blue and green categories. Also what is MNP ?

Ans: We have updated the figure with more distinguishable colours for the legend categories. MNP refers to Multiple Nucleotide Polymorphism, which involves two or more adjacent base substitutions occurring together.

* Line 435: Authors meant this as Mingraph instead of Minmap2

Ans: Thank you, this has now been corrected.

Reviewer #2: In this study, the authors compared three pangenomic methods Minigraph, PGGB, and Minigraph-Cactus using sorghum genome dataset. In general, I think this study was well-designed and the results could be extended in pangenomic studies of other plants. However, I also have the following comments for the authors to consider:

1. The authors only used sorghum as an example, which is biased, as the genome structure and data quality have significant impacts on pangenomic and downstream genetic analyses. I recommend the authors to use several representative plant species to compare the pangenomic pipelines and the generated evaluation metrics.

Ans: Thank you for this valuable suggestion. We have expanded our study to include three additional plant species *Glycine max* (soybean), *Brassica napus* (canola), and *Hordeum vulgare* (barley) which represent a diverse range of genome sizes, ploidy levels, and assembly qualities. By applying the same pangenome construction and variant calling pipelines across these species, we were able to evaluate the generalizability and robustness of the methods beyond *Sorghum bicolor*. The comparative analyses of graph sizes, completeness, duplication rates, and variant detection metrics reinforced the trends observed in sorghum. We have incorporated these results and related discussion into the manuscript to better reflect the broader applicability of the pangenome pipelines across diverse crop genomes.

2. The quality of the figures presented in this manuscript are low. Please use standard tools to regenerate the figures.

Ans: We have regenerated all figures using standard tools to ensure high resolution and clarity. The updated figures now have a resolution of 600 dpi, which significantly improves their quality.

3. Pangenome has been extensively studied in many plants, the authors should compare their conclusions with published ones.

Ans: Thank you for the suggestion. We added a section discussing published pangenome studies where assessment of pangenome graph-based approaches for variant discovery/genotyping was performed (Lines 548-567).

We also added a section to the introduction discussing advantages of graphs and referring to studies, which used pangenome graphs (Lines 84-94).
