# Supplementary material for: Toward a standardized framework for pangenome graph evaluation: assessing crop plant pangenome variation graph construction from multiple assemblies
Source: Gigascience. 2025 Dec 4;14:giaf121. doi: 10.1093/gigascience/giaf121 (PMC12676463; doi:10.1093/gigascience/giaf121)
Supplement: giaf121_Authors_Response_To_Reviewer_Comments_Original_Submission [file giaf121_authors_response_to_reviewer_comments_original_submission.pdf]

Author's Response To Reviewer Comments

Reviewer reports:

Reviewer #1: The authors provide a good summary of pan genome Graph evaluations based on Sorghum pan genome assemblies. They provide details insights into strengths and limitation for 3 pan-genome graph construction tools Mingraph, PGGN and Monograph-Cactus. They propose metrics to evaluate comprehensive pan genome graph evaluation, which could be applied or standardized of other pan genome graph construction methods.

Major comments:
* Introduction; the authors although highlight the importance of Pan-genomes and what value they offer. Could the authors also comment on why pan genome variation graph are important; specifically what advantages they offer over traditional linear based pan genomes.

Ans: Thank you for your feedback, we have now added a paragraph (Lines 84-94) in the Introduction highlighting the importance of variation graphs.

* Methods:
- Genome assembly and chromosome splitting-
- Although the authors have chosen accession that are diverse. Could they comment also on the quality of the assembly. For building graph-based pan genomes having high quality assemblies are crucial to avoid artificial bubbles or paths in the graphs that are built with the tools used for benchmarking. How much does the quality of assembly inflate the complexity of the pan-genome graphs built. Some background of the quality of the assembly would help in here to understand the benchmarking metrics that authors are testing

Ans: All selected genomes were assembled using both Illumina short reads and PacBio long reads, resulting in high-quality assemblies with contig N50 values up to 3.5 Mb and BUSCO completeness scores exceeded 95%, indicating strong representation of the gene space. (Added in lines 123-124)

- Simulated Genomes Assemblies-
- Can you clarify if the SV's used with VISOR for generating simulated assemblies are real or are the SV's provided simulated too?

Ans: Thank you, we have now clarified in the text that the SV's used with VISOR are real SVs. (Line 135)

* Read Mapping
- Line 404 : Generating mapping indexes with PGGB pan genome graph was mentioned to be difficult due to the graph complexity and heavy computational demand. Could authors try read mapping with smaller subset graph and generate index for the smaller graph. Maybe reduce the graph to chr level?

Ans: Thank you for the suggestion. We revisited the task of generating mapping indexes for the PGGB pangenome graph. We were able to successfully build whole-genome mapping indexes, but the process required approximately 2.2 TB of memory. The resulting index files were extremely large, and attempting to map reads back to them would be computationally expensive in most practical scenarios.
We also tested building indexes at the chromosome level, which was feasible with much lower resource requirements. However, the utility of individually indexed chromosomes for genome-wide analyses is limited (https://github.com/vgteam/vg/issues/3841).

- Line 417: what is correctly mapped reads? min_mapQ > 60 ?

Ans: Correctly mapped reads are simulated reads that align back to their original genomic position from where they were simulated from. 'min_mapQ 60' stands for a minimum mapping quality score of 60. This has now been clarified in the manuscript.

- Fig12: It was really hard to read and follow the figure and text in the main section. Suggest Figure 12 split as a,b,c, & d and refer them in the main text. Maybe also uses lines in the figure to show boundaries.

Ans: Thank you for the suggestion. We revisited Figure 12 and agree that the original layout was difficult to follow. To improve clarity and readability, we replaced it with a table (now Supplementary Table 1), which presents the data in a more accessible and organized format.

Minor comments
* Line 294: It should be mentioned as Figure 3 instead of Figure 2
Ans: Thank you, this has now been corrected.

* Suggest combining Fig 3, 4, & 5 as a facet plot for better readability and comparison
Ans: We combined Figures 3, 4, and 5 into a single facet plot, this updated figure is now presented as Figure 3

* Suggest combining Fig 6, 7, & 8 as a facet plot for better readability and comparison
Ans: We combined Figures 6, 7, and 8 into a single facet plot, this updated figure is now presented as Figure 4

* Figure 9 : Suggest using different choice for legends. Hard to distinguish blue and green categories. Also what is MNP ?
Ans: We have updated the figure with more distinguishable colours for the legend categories. MNP refers to Multiple Nucleotide Polymorphism, which involves two or more adjacent base substitutions occurring together.

* Line 435: Authors meant this as Mingraph instead of Minmap2
Ans: Thank you, this has now been corrected.

Reviewer #2: In this study, the authors compared three pangenomic methods Minigraph, PGGB, and Minigraph-Cactus using sorghum genome dataset. In general, I think this study was well-designed and the results could be extended in pangenomic studies of other plants. However, I also have the following comments for the authors to consider:

1. The authors only used sorghum as an example, which is biased, as the genome structure and data quality have significant impacts on pangenomic and downstream genetic analyses. I recommend the authors to use several representative plant species to compare the pangenomic pipelines and the generated evaluation metrics.

Ans: Thank you for this valuable suggestion. We have expanded our study to include three additional plant species Glycine max (soybean), Brassica napus (canola), and Hordeum vulgare (barley) which represent a diverse range of genome sizes, ploidy levels, and assembly qualities. By applying the same pangenome construction and variant calling pipelines across these species, we were able to evaluate the generalizability and robustness of the methods beyond Sorghum bicolor. The comparative analyses of graph sizes, completeness, duplication rates, and variant detection metrics reinforced the trends observed in sorghum. We have incorporated these results and related discussion into the manuscript to better reflect the broader applicability of the pangenome pipelines across diverse crop genomes.

2. The quality of the figures presented in this manuscript are low. Please use standard tools to regenerate the figures.

Ans: We have regenerated all figures using standard tools to ensure high resolution and clarity. The updated figures now have a resolution of 600 dpi, which significantly improves their quality.

3. Pangenome has been extensively studied in many plants, the authors should compare their conclusions with published ones.

Ans: Thank you for the suggestion. We added a section discussing published pangenome studies where assessment of pangenome graph-based approaches for variant discovery/genotyping was performed (Lines 548-567).
We also added a section to the introduction discussing advantages of graphs and referring to studies, which used pangenome graphs (Lines 84-94).

Editorial Manager®

Cookie Preference Center

We use cookies which are necessary to make our site work. We may also use additional cookies to analyze, improve and personalize our content and your digital experience. For more information, see our Cookie Policy.

You may choose not to allow some types of cookies. However, blocking some types may impact your experience of our site and the services we are able to offer. See the different category headings below to find out more or change your settings.

Allow all

Manage Consent Preferences

Strictly Necessary Cookies

Always active

These cookies are necessary for the website to function and cannot be switched off in our systems. They are usually only set in response to actions made by you which amount to a request for services, such as setting your privacy preferences, logging in or filling in forms. You can set your browser to block or alert you about these cookies, but some parts of the site will not then work.

Functional Cookies

These cookies enable the website to provide enhanced functionality and personalisation. They may be set by us or by third party providers whose services we

have added to our pages. If you do not allow these cookies then some or all of these services may not function properly.

Performance Cookies

These cookies allow us to count visits and traffic sources so we can measure and improve the performance of our site. They help us to know which pages are the most and least popular and see how visitors move around the site.

Targeting Cookies

These cookies may be set through our site by our advertising partners. They may be used by those companies to build a profile of your interests and show you relevant adverts on other sites. If you do not allow these cookies, you will experience less targeted advertising.

Cookie List

Clear

Apply Cancel
Consent Leg.Interest

Confirm my choices

Powered by onetrust
