# Supplementary material for: Toward a standardized framework for pangenome graph evaluation: assessing crop plant pangenome variation graph construction from multiple assemblies
Source: Gigascience. 2025 Dec 4;14:giaf121. doi: 10.1093/gigascience/giaf121 (PMC12676463; doi:10.1093/gigascience/giaf121)
Supplement: giaf121_Reviewer_1_Report_Original_Submission — Kapeel M. Chougule -- 4/14/2025 [file giaf121_reviewer_1_report_original_submission.pdf]

Reviewer Report

Title: Towards a Standardized Framework for Pangenome Graph Evaluation: Assessing Crop Plant Pangenome Variation Graph Construction from Multiple Assemblies

Version: Original Submission **Date:** 4/14/2025

Reviewer name: Kapeel M. Chougule

Reviewer Comments to Author:

The authors provide a good summary of pan genome Graph evaluations based on Sorghum pan genome assemblies. They provide details insights into strengths and limitation for 3 pan-genome graph construction tools Mingraph, PGGN and Monograph-Cactus. They propose metrics to evaluate comprehensive pan genome graph evaluation, which could be applied or standardized of other pan genome graph construction methods.
Major comments:
* Introduction; the authors although highlight the importance of Pan-genomes and what value they offer. Could the authors also comment on why pan genome variation graph are important ; specifically what advantages they offer over traditional linear based pan genomes.
* Methods:
â▯ƒ Genome assembly and chromosome splitting-
â▯ƒ Although the authors have chosen accession that are diverse. Could they comment also on the quality of the assembly. For building graph-based pan genomes having high quality assemblies are crucial to avoid artificial bubbles or paths in the graphs that are built with the tools used for benchmarking. How much does the quality of assembly inflate the complexity of the pan-genome graphs built. Some background of the quality of the assembly would help in here to understand the benchmarking metrics that authors are testing
â▯ƒ Simulated Genomes Assemblies-
â▯ƒ Can you clarify if the SV's used with VISOR for generating simulated assemblies are real or are the SV's provided simulated too?
* Read Mapping
â▯ƒ Line 404 : Generating mapping indexes with PGGB pan genome graph was mentioned to be difficult due to the graph complexity and heavy computational demand. Could authors try read mapping with smaller subset graph and generate index for the smaller graph. Maybe reduce the graph to chr level?
â▯ƒ Line 417: what is correctly mapped reads? min_mapQ > 60 ?
â▯ƒ Fig12: It was really hard to read and follow the figure and text in the main section. Suggest Figure 12 split as a,b,c, & d and refer them in the main text. Maybe also uses lines in the figure to show boundaries.
Minor comments
* Line 294: It should be mentioned as Figure 3 instead of Figure 2
* Suggest combining Fig 3, 4, & 5 as a facet plot for better readability and comparison
* Suggest combining Fig 6, 7, & 8 as a facet plot for better readability and comparison

* Figure 9 : Suggest using different choice for legends. Hard to distinguish blue and green categories. Also what is MNP ?
* Line 435: Authors meant this as Mingraph instead of Minmap2

Methods

Are the methods appropriate to the aims of the study, are they well described, and are necessary controls included? Choose an item.

Conclusions

Are the conclusions adequately supported by the data shown? Choose an item.

Reporting Standards

Does the manuscript adhere to the journal's guidelines on [minimum standards of reporting?]() Choose an item.

Choose an item.

Statistics

Are you able to assess all statistics in the manuscript, including the appropriateness of statistical tests used? Choose an item.

Quality of Written English

Please indicate the quality of language in the manuscript: Choose an item.

Declaration of Competing Interests

Please complete a declaration of competing interests, considering the following questions:

- Have you in the past five years received reimbursements, fees, funding, or salary from an organisation that may in any way gain or lose financially from the publication of this manuscript, either now or in the future?
- Do you hold any stocks or shares in an organisation that may in any way gain or lose financially from the publication of this manuscript, either now or in the future?
- Do you hold or are you currently applying for any patents relating to the content of the manuscript?
- Have you received reimbursements, fees, funding, or salary from an organization that holds or has applied for patents relating to the content of the manuscript?
- Do you have any other financial competing interests?
- Do you have any non-financial competing interests in relation to this paper?

If you can answer no to all of the above, write 'I declare that I have no competing interests' below. If your reply is yes to any, please give details below.

I declare that I have no competing interests

I agree to the open peer review policy of the journal. I understand that my name will be included on my report to the authors and, if the manuscript is accepted for publication, my named report including any attachments I upload will be posted on the website along with the authors' responses. I agree for my report to be made available under an Open Access Creative Commons CC-BY license (http://creativecommons.org/licenses/by/4.0/). I understand that any comments which I do not wish to be included in my named report can be included as confidential comments to the editors, which will not be published.

Choose an item.

To further support our reviewers, we have joined with Publons, where you can gain additional credit to further highlight your hard work (see: https://publons.com/journal/530/gigascience). On publication of this paper, your review will be automatically added to Publons, you can then choose whether or not to claim your Publons credit. I understand this statement.

Yes Choose an item.
